# Supplementary material for: Healing The Past By Nurturing The Future: A qualitative systematic review and meta-synthesis of pregnancy, birth and early postpartum experiences and views of parents with a history of childhood maltreatment
Source: PLoS One. 2019 Dec 13;14(12):e0225441. doi: 10.1371/journal.pone.0225441 (PMC6910698; doi:10.1371/journal.pone.0225441)
Supplement: S2 Appendix — A sample of the search strategy used by this review with the PsychInfo database. (DOCX) [file pone.0225441.s002.docx]

## **S2 Appendix: Sample search strategy**

Database: PsycINFO <1806 to June Week 1 2018>

**Concept 1: Prenatal to postnatal care, transition to parenting, parenting or parents (**lines 1-12, combined by line 14)

1 (Parenting or maternity or midwife* or fatherhood or motherhood or mothering or fathering or (women adj2 (birth or labour or labor)) or (Pregnant adj3 women) or (Pregnant adj3 adolescent*) or "maternal care*" or "maternal behavio?r" or obstetric or obstetrics or "maternal health" or "maternal healthcare" or ((conception or preconception or antenatal or antepartum* or "ante natal" or "ante partum" or prenatal or prenatal or postnatal or parturition or pregnancy or primipara* or primigravid* or perinatal or postpartum or "post partum" or postpartum or childbirth or childbearing) adj2 (care or healthcare or health)) or ((conception or preconception or antenatal or antepartum* or "ante natal" or "ante partum" or prenatal or prenatal or postnatal or parturition or pregnancy or primipara* or primigravid* or perinatal or postpartum or "post partum" or postpartum or childbirth or childbearing) and (women or mother?))).ti,ab. (82808)

2 (parenthood or motherhood or fatherhood or parentificat*).ti,ab. (11959)

3 ((parental or maternal or paternal) adj (care* or relations* or behavio* or interact* or bonding)).ti,ab. (12484)

4 ((new or young or "first time") adj (Parents or Mothers or Fathers)).ti,ab. (3217)

5 ((mother? or father? or parent?) adj15 ((raise or raising) adj2 child*)).ti,ab. (1045)

6 ((Parents or Mothers or Fathers) and (breastfeed* or "young children" or "small children" or toddlers or infants or preschool* or baby or babies or newborn* or "new born?" or "new-born?" or caregiving or "early years" or nursery)).ti,ab. (40307)

7 ((parent* or mother* or father*) adj5 (Adaptation or Adjustment or Transform* or transition*)).ti,ab. (8665)

8 ((Parent* or Mother* or Father*) adj2 (becoming or become or expect*)).ti,ab. (6343)

9 ((parental or maternal or paternal or mothers or fathers or parents) and (intergeneration* or transgeneration* or familial)).ti,ab. (9506)

10 exp EXPECTANT PARENTS/ or adolescent mothers/ or expectant mothers/ or ((exp PARENTS/ or exp SINGLE PARENTS/ or mothers/) and (child/ or "child, preschool"/ or early experience/ or pregnancy/ or perinatal period/ or postnatal period/ or primipara/)) (7448)

11 permissive parenting/ or child discipline/ or parenting/ or authoritarian parenting/ or authoritative parenting/ or exp childrearing practices/ or father child relations/ or mother child relations/ or exp parent child relations/ or exp parental attitudes/ or parental role/ or exp parenting skills/ or parental characteristics/ or parent training/ or exp parental attitudes/ or exp child discipline/ or exp parent child communication/ or parenting skills/ or exp parenting style/ or transgenerational patterns/ or childrearing attitudes/ or family planning attitudes/ or parental expectations/ or "labor (childbirth)"/ or midwifery/ or obstetrics/ or exp prenatal care/ or adolescent pregnancy/ or childbirth training/ (107557)

12 ((mothers or fathers or single parent or parents) and (child, preschool or infant or infant, newborn or child development)).mh. (11551)

13 (child rearing or parenting or Parent-Child Relations or Father-child relations or mother-child relations or Maternal-Fetal Relations or paternal behavior or maternal behavior or intergenerational relations or labor, obstetric or pregnant women or prenatal care or obstetrics or obstetric nursing or Maternal-Child Nursing or preconception care or Maternal Health or perinatal care or midwifery).mh. (38750)

14 1 or 2 or 3 or 4 or 5 or 6 or 7 or 8 or 9 or 10 or 11 or 12 or 13 (207298)

**Concept 2: Intergenerational abuse (line 15)**

15 ((parentification adj1 histor*) or (((Intergenerational or transgenerational or (across adj2 generation*) or (cross* adj generation*) or ((break or breaking or breaks or broke) adj3 (cycle* or pattern*))) and (abus* or trauma* or neglect* or maltreatment or violence or posttrauma* or "post-trauma*" or "toxic stress")) or (cycle adj2 (abus* or trauma* or neglect* or maltreatment or violence or posttrauma* or "post-trauma*" or "toxic stress")) or (((Transmission or transmit*) adj2 (generation* or family pattern*)) and (abus* or trauma* or neglect* or maltreatment or violence or posttrauma* or "post-trauma*" or "toxic stress")) or (((Transmission or transmit*) adj2 (abus* or trauma* or neglect* or maltreatment or violence or posttrauma* or "post-trauma*" or "toxic stress")) or (Cycle adj2 (abus* or trauma* or neglect* or maltreatment or violence or posttrauma* or "post-trauma*" or "toxic stress")) or (Abusive adj families)) or ((abus* or trauma* or neglect* or maltreatment or violence or posttrauma* or "post-trauma*" or "toxic stress") adj2 (continuity or discontinuity or legacy)))).ti,ab. (3545)

**Concept 2: History of abuse (lines 16-18)**

16 ((History or histories or surviv* or recovery or unresolved or past) adj5 (Abus* or neglect* or trauma or maltreatment or stress)).ti,ab. (23723)

17 ((Childhood or children or "as a child" or "as an infant" or "early life") adj3 (Abused or mistreated or neglected or maltreated)).ti,ab. (5273)

18 "Adult survivors of child abuse".mh. (1038)

**Concept 2: Post-traumatic stress – (lines 19-22)**

19 ("Posttraumatic stress*" or "Post traumatic stress*" or "Post-traumatic stress*" or "relational trauma" or "developmental trauma" or "toxic stress" or "trauma-informed" or "complex trauma").ti,ab. (35560)

20 complex ptsd/ or posttraumatic stress disorder/ or desnos/ or post-traumatic stress/ (29524)

21 ("Stress Disorders, Post-Traumatic" or "Psychological Trauma").mh. (16416)

22 15 or 16 or 17 or 18 or 19 or 20 or 21 (70016)

**Concept 2: Survivors or history (line 21-25) AND Child abuse (lines 26-29)**

23 survivors.mh. or survivors/ or "medical history taking".mh. (15052)

24 (History or histories or historical or Survivor* or experiences* or experienced* or victim* or exposure or exposed or "unresolved past" or "past experience" or (generation* adj2 (continuity or discontinuity))).ti,ab. (724069)

25 23 or 24 (725799)

26 ((Childhood or children or Child*) adj5 (Abuse or abused or abusive or neglect or maltreat* or incest or assault or trauma)).ti,ab. (39932)

27 ("Adverse childhood experience*" or "adverse childhood events" or "aversive childhood experience*" or "childhood adversity" or "early life trauma" or "traumatic childhood experience*").ti,ab. (2154)

28 Child Abuse/ or battered child syndrome/ or child abuse, sexual/ or child neglect/ or ("child abuse" or "child abuse, sexual").mh. (33191)

29 26 or 27 or 28 (49402)

**Concept 1 and 2: Parental post-traumatic stress / relational trauma (line 30)**

30 ((parental or maternal or paternal or mothers or fathers or parents) adj8 ("Posttraumatic stress*" or "Post traumatic stress*" or "Post-traumatic stress*" or "relational trauma" or "developmental trauma" or "toxic stress" or "trauma-informed" or "complex trauma")).ti,ab. (643)

31 14 and (15 or 22 or (25 and 29)) (8612) **(Concepts 1 and 2 combined, except for line 30)**

32 30 or 31 (8842) **(Concepts 1 and 2)**

33 limit 32 to english language (8271)

34 limit 33 to human (8102)
