# Supplementary material for: Healing The Past By Nurturing The Future: A qualitative systematic review and meta-synthesis of pregnancy, birth and early postpartum experiences and views of parents with a history of childhood maltreatment
Source: PLoS One. 2019 Dec 13;14(12):e0225441. doi: 10.1371/journal.pone.0225441 (PMC6910698; doi:10.1371/journal.pone.0225441)
Supplement: S3 Appendix — A copy of the Nvivo file attributes imported from the Excel data extraction spreadsheet. (DOCX) [file pone.0225441.s003.docx]

## **S3 Appendix: Excel study data extraction imported into Nvivo as file attributes**

|  |  |  |
| --- | --- | --- |
| **Study ID / Inclusion details** | **Assoc refs (red if in this review)*** |  |
|  | **Title*** |  |
|  | **Author names (red similar authors)*** |  |
|  | **Study name*** |  |
|  | **Is this same study as other included reference? (y/n/u/na)*^** |  |
|  | **If yes, are participants the same? (y/n/u/na/p)*^** |  |
|  | **If yes, what is the Primary study ID for this reference (specify or na)*** |  |
|  | **Is this study; primary; associated ref; or other^** |  |
|  | **Comments (if other)** |  |
|  |  |  |
| **1. Data extraction and author** | **Date data extraction completed (dd/mm/yyyy)*** |  |
|  | **Initials of person/s extracting data*** |  |
|  | **DE agreed date** |  |
|  | **Author contact details [email]*** |  |
|  | **Study funding source [if specified, or ‘not specified’]*** |  |
|  | **Risk of Possible conflicts of interest**  **[authors] (H/L/U)*^** |  |
|  |  |  |
| **2. Sample characteristics** | **Setting: country [describe]*^** |  |
|  | **Country if other** |  |
|  | **Setting: Description of location and social context*** |  |
|  | **Setting: Start and end dates of study (or ‘not specified’)*** |  |
|  | **Inclusion criteria [copy if specified or ‘not specified’]*?** |  |
|  | **Exclusion criteria [copy if specified or ‘not specified’]*?** |  |
|  | **Equity pointer: Could disadvantaged populations have been excluded from the study?(y/n/u) ^ (From ROB in scoping study)** |  |
|  | **Equity pointer comments** |  |
|  | **Brief description of how participants were recruited*** |  |
|  | **Total number approached [if known or ‘not specified’]*** |  |
|  | **Total number included [if known and by intervention group if appropriate or ‘not specified’] [participation rate=12/11]*** |  |
|  | **Number parents for synthesis*** |  |
|  | **Number of withdrawals/dropouts and reasons (if specified, and any characteristics of those dropping out)*** |  |
|  | **Specify stage of parenting or age of children are included, or 'not specified'*** |  |
|  | **Does this study involve parents *Before pregnancy (e.g. pre-pregnancy/family planning/preconception clinics)* (y/n/u)*^** |  |
|  | **Does this study involve parents *During pregnancy and up to 6 weeks postpartum (y/n/u)*^*** |  |
|  | **Does this study involve parents *After 6 weeks postpartum up to 1 year of age (y/n/u)*^*** |  |
|  | **Does this study involve parents of children *aged from 13 months to 2 years? (y/n/u/)*^*** |  |
|  | **Describe the type of trauma parents in this study have experienced, or 'not specified'*** |  |
|  | **Does this study report including parents who have experienced Self-reported childhood emotional abuse (complex trauma) (y/n/u/)*^ (if inferred, note in free box and mark as unclear)** |  |
|  | **Does this study report including parents who have experienced Self-reported childhood sexual abuse (complex trauma) (y/n/u/)*^ (if inferred note in free text box and mark as unclear)** |  |
|  | **Does this study report including parents who have experienced Self-reported childhood Physical abuse (complex trauma) (y/n/u/)*^ (if inferred note in free text box and mark as unclear)** |  |
|  | **Does this study report including parents who have experienced Any type of ‘adverse childhood experiences’ (as per definition) (y/n/u/)*^ (if inferred note in free text box and mark as unclear)** |  |
|  | **Does this study report including parents who have Substantiated child abuse (any type) by child protection services (y/n/u/)*^ (if inferred note in free text box and mark as unclear)** |  |
|  | **Does this study report including parents who were Removed from families due to substantiated child abuse (y/n/u/)*^ (if inferred note in free text box and mark as unclear)** |  |
|  | **Does this study report including parents who have experienced Emotional neglect (y/n/u/)*^ (if inferred note in free text box and mark as unclear)** |  |
|  | **Does this study report including parents who have experienced Physical Neglect (y/n/u/)*^ (if inferred note in free text box and mark as unclear)** |  |
|  | **Does this study report including parents who have experienced Neglect otherwise unspecified (y/n/u/)*^ (if inferred note in free text box and mark as unclear)** |  |
|  | **Does this study report including parents who have experienced witnessing IPV (y/n/u/)*^ (if inferred note in free text box and mark as unclear)** |  |
|  | **Does this study report including parents who have experienced Witnessing other violence (eg shootings) (y/n/u/)*^ (if inferred note in free text box and mark as unclear)** |  |
|  | **Does this study report including parents who have experienced any other type of trauma not previously specified? (y/n/u/)*^- and specify if yes) (if inferred note in free text box and mark as unclear)** |  |
|  | **Other types of parental trauma (specify)** |  |
|  | **Does the study include parents who have AND have NOT experienced trauma? E.g for comparison (y/n/u/)** |  |
|  |  |  |
| **3. Equity criteria (Progress Plus)** | **Is the sample selected from any other 'at risk' area (other than for child maltreatment history) e.g. mental health service, prison, drug rehab centre, child maltreatment register (y/n/u/)^** |  |
|  | **Describe if yes** |  |
|  | **Are any socio-demographic or PROGRESS plus criteria reported for the population? (y/n/u)^** |  |
|  | **Age of population [if specified or ‘not specified’] [Note if specifically aimed at young adults/parents]*** |  |
|  | **mean age*** |  |
|  | **Standard deviation*** |  |
|  | **age range (lower)*** |  |
|  | **age range (upper)*** |  |
|  | **Sex of population included*^** |  |
|  | **Are PROGRESS+ socio-demographic characteristics reported for Place of residence (e.g. economically deprived areas] (y/n/u)*^** |  |
|  | **Place of residence details if yes*** |  |
|  | **Are PROGRESS+ socio-demographic characteristics reported for Race/ethnicity (including Indigenous status) (y/n/u)*^** |  |
|  | **Race/ethnicity details if yes** |  |
|  | **Mostly (>50%) majority/dominant population (white)(y/n/u/na)*^** |  |
|  | **Mostly (>50%) minority populations (y/n/u/na) *^** |  |
|  | **Any identified as Indigenous (y/n/u/na)*^** |  |
|  | **Are PROGRESS+ socio-demographic characteristics reported for Language other than English (y/n/u)*^** |  |
|  | **Language information** |  |
|  | **Are PROGRESS+ socio-demographic characteristics reported for Education status (y/n/u)*^** |  |
|  | **Education status if yes** |  |
|  | **Majority college graduate, Majority high school only, Majority less than high school/GED; other *^** |  |
|  | **large proportion (>20% not finished high school) (y/n/u/na)*^** |  |
|  | **Are PROGRESS+ socio-demographic characteristics reported for Socio-economic status (y/n/u)*^** |  |
|  | **SES details if yes** |  |
|  | **Majority low SES (y/n/u/na)*^** |  |
|  | **Are PROGRESS+ socio-demographic characteristics reported for Social capital (e.g. low levels of social support/single parents) (y/n/u)*^** |  |
|  | **Social capital details if yes** |  |
|  | **Majority (>50%) single (unmarried/no partner) parents (y/n/u/na)*^** |  |
|  | **High proportion sole parents (>20%) (y/n/u/na)*^** |  |
|  | **Majority other Low levels of social support (y/n/u/na)*^** |  |
|  | **Are PROGRESS+ socio-demographic characteristics reported for Other (e.g. mental illness, substance misuse, infants with FASD, imprisonment/justice involvement) (y/n/u)*^** |  |
|  | **Description other PROGRESS + characteristics if yes** |  |
|  | **Majority mental illness (y/n/u/na)*^** |  |
|  | **Majority Drug use (y/n/u/na)*^** |  |
|  | **Majority IPV (y/n/u/na)*^** |  |
|  | **Majority CPS involvement (y/n/u/na)*^** |  |
|  | **Progress + Other comments** |  |
|  |  |  |
| **4. Phenomena of Interest** | **Brief description of aim of study*** |  |
|  | **Does the study report parent or provider views?** |  |
|  |  |  |
| **Provider information if Both (including number)** | **If views other than parent or provider, describe** |  |
|  | **Does the study explore perinatal care experiences? (y/n/u)** |  |
|  | **Does the study explore barriers and/or enablers to perinatal care? (y/n/u)** |  |
|  | **Does the study explore parenting transition experiences? (y/n/u)** |  |
|  | **Does the study explore parent aspirations or challenges? (y/n/u)** |  |
|  | **Does the study describe parental strategies that help or hinder healing or transmission of intergenerational trauma? (y/n/u)** |  |
|  | **Does the study explore other relevant phenomena for this review? (y/n/u)** |  |
|  | **Included in PregnancyBirthEarlyPP Review** |  |
|  | **Included in ParentingTransition Review** |  |
|  | **Describe other relevant phenomena if yes** |  |
|  |  | |
| **5. Risk of bias assessment (CASP)** | **1. Is there a clear description of study aims? (Y=low ROB, N=High ROB, Unclear)*^** |  |
|  | **ROB 1 Comment** |  |
|  | **2. Is the qualitative methodology appropriate for this study? (Y=low ROB, N=High ROB, Unclear)*^** |  |
|  | **ROB 2 Comment** |  |
|  | **3. Was the research design appropriate to address the aims of the research? (Y=low ROB, N=High ROB, Unclear)^** |  |
|  | **ROB 3 Comment** |  |
|  | **4. Was the recruitment/ sampling strategy appropriate to address the aims of the research? (Y=low ROB, N=High ROB, Unclear)*^** |  |
|  | **ROB 4 Comment** |  |
|  | **5. Was the data collected in a way that addressed the research issue? (Y=low ROB, N=High ROB, Unclear)*^** |  |
|  | **ROB 5 Comment** |  |
|  | **6. Has the relationship between researcher and participants been adequately considered? (Y=low ROB, N=High ROB, Unclear)*^** |  |
|  | **ROB 6 Comment** |  |
|  | **7. Have ethical issues been taken into consideration? (Y=low ROB, N=High ROB, Unclear)*^** |  |
|  | **ROB 7 Comment** |  |
|  | **8. Was the data analysis sufficiently rigorous? (Y=low ROB, N=High ROB, Unclear)*^** |  |
|  | **ROB 8 Comment** |  |
|  | **9. Is there a clear statement of findings? (Y=low ROB, N=High ROB, Unclear)^** |  |
|  | **ROB 9 Comment** |  |
|  | **10. Is the research valuable? (Y=low ROB, N=High ROB, Unclear)^** |  |
|  | **ROB 10 Comments** |  |
|  | **Other ROB comments** |  |
| Overall ROB (modified GRADE) within study | Comments on reasons for any downgrade: Start at High and downgrade (-1) for serious concerns or (-2) for very serious concerns to Moderate, Low or Very low based on:  1. **Methodological limitations** (concerns about whether qualitative method is appropriate or researcher-participant relationship considered)  2. **Relevance** (concerns whether data collection addresses research issue)  3. **Adequacy of data** (concerns about sampling strategy; analysis approach or data richness) |  |
