# Supplementary figures and images for: Healing The Past By Nurturing The Future: A qualitative systematic review and meta-synthesis of pregnancy, birth and early postpartum experiences and views of parents with a history of childhood maltreatment
Source: PLoS One. 2019 Dec 13;14(12):e0225441. doi: 10.1371/journal.pone.0225441 (PMC6910698; doi:10.1371/journal.pone.0225441)

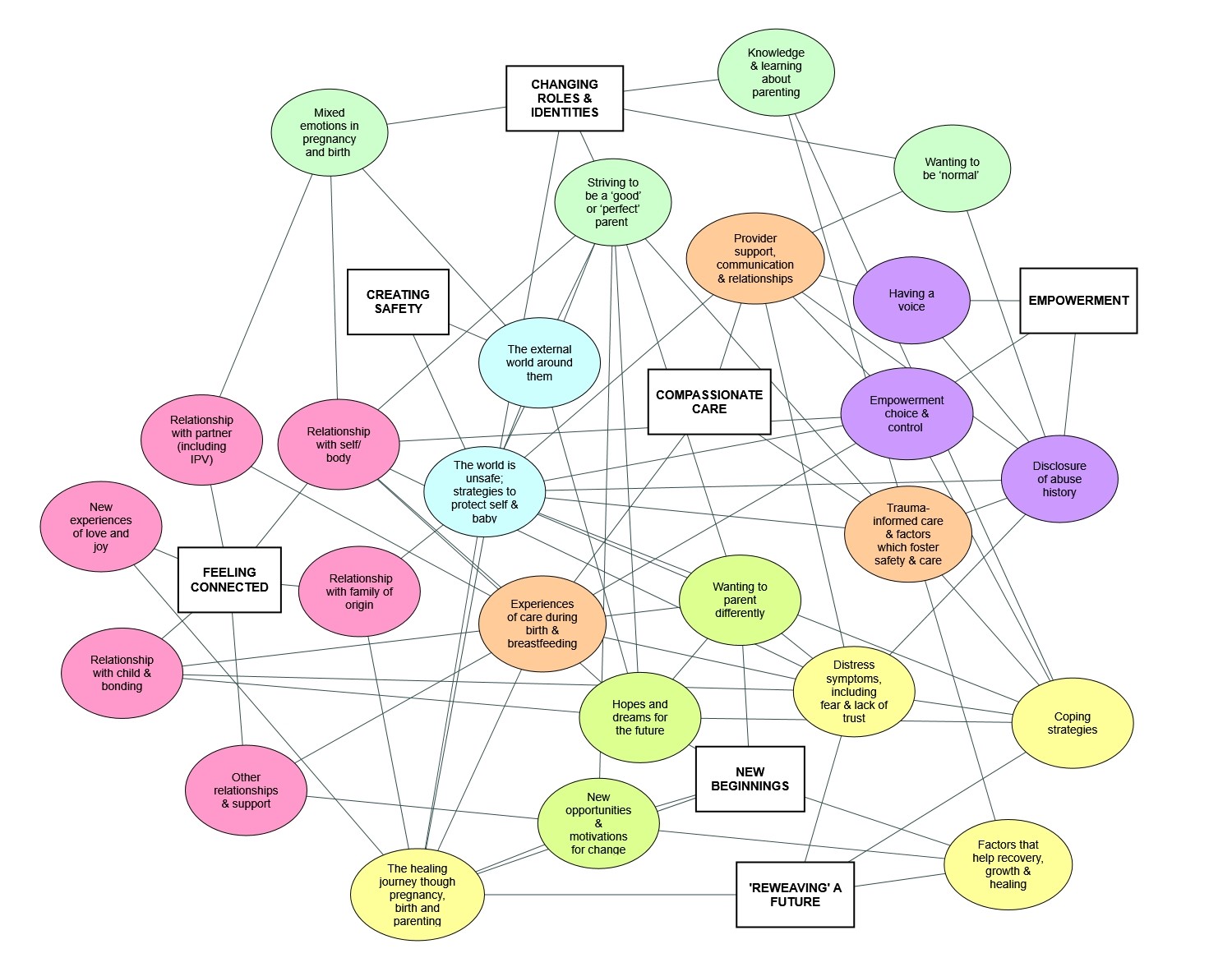

Supplement: S4 Appendix — The concept map of analytic themes and descriptive subthemes generated by this review. (JPG) [file pone.0225441.s004.jpg]
