# Supplementary material for: Healing The Past By Nurturing The Future: A qualitative systematic review and meta-synthesis of pregnancy, birth and early postpartum experiences and views of parents with a history of childhood maltreatment
Source: PLoS One. 2019 Dec 13;14(12):e0225441. doi: 10.1371/journal.pone.0225441 (PMC6910698; doi:10.1371/journal.pone.0225441)
Supplement: S5 Appendix — Summary table of analytic themes, descriptive subthemes, and axial codes generated by this review. (DOCX) [file pone.0225441.s005.docx]

## **S5 Appendix:** **Summary of analytic themes, descriptive subthemes and axial codes**

Note colour coding of axial nodes: Green - *positive experiences*; red - *challenges*; purple - *things that help*; orange - *diverse experiences* between study participants; yellow - *internal conflicting experiences* within study participants.

| **Analytic theme** | **Descriptive subtheme** |  | | | | **Axial code in NVivo** | **No. of relevant articles and references** |
| --- | --- | --- | --- | --- | --- | --- | --- |
| 1. *New beginnings*: Becoming a parent is an opportunity for ‘a fresh start’, to put the past behind them and move forward with hope for the future to create a new life for themselves and their child | 1.1 New opportunities and motivations for change |  | | | | New opportunity or fresh start  Self-care or take care of self for baby  Maturing or changing behaviour for the baby | 10 [1-10]  3 [2, 3, 11]  3 [2, 6, 8] |
|  | *1.2 Hopes and dreams for the future* |  | | | | Hopes or dreams for the future  Stable home life or family  Stable or safe relationship  Hoped their partner would be a good father  Hopes for their child  A ‘perfect’ or idealised family  Being a ‘good mother’ | 6 [1, 5, 8-10, 12]  5 [1, 2, 7, 8, 12]  1 [2]  1 [8]  2 [4, 9]  2 [1, 6]  4 [1-3, 8] |
|  | *1.3 Wanting to parent differently* |  | | | | Desire to not repeat the past  Wanting to parent differently  Fear of repeating the past  Wanting to be emotionally responsive | 5 [2, 6, 8, 13, 14]  6 [1, 2, 6, 7, 11, 14]  7 [2, 4-6, 11, 13, 14]  5 [2, 4, 8, 9, 15] |
|  |  |  | | | |  |  |
|  |  |  | | | |  |  |
|  |  |  | | | |  |  |
| 2. *Changing roles and identities*: Becoming a parent is a major life transition, influenced by perceptions of the parenting role. | 2.1 *Mixed emotions in pregnancy and birth* |  | | | | Conflicting internal emotions about pregnancy  Becoming pregnant (positive experiences)  Hopes and fears  Impact of pregnancy on their current lifestyle  Postpartum adjustment | 9 [1, 2, 4, 6-8, 15-17]  5 [2, 4, 8, 15, 18]  1 [4]  2 [2, 8]  1 [6] |
|  |  |  | | | |  |  |
|  |  |  | | | |  |  |
|  |  |  | | | |  |  |
|  |  |  | | | |  |  |
|  | *2.2 Striving to be a ‘good’ or ‘perfect’ parent* |  | | | | Delaying pregnancy  Childless by choice  Responsibility or purpose  Determination  Regain a sense of self-worth | 5 [2, 3, 11, 19, 20]  1 [6]  6 [2-4, 6, 7, 21]  1 [4]  2 [2, 8] |
|  |  |  | | | |  |  |
|  |  |  | | | |  |  |
|  |  |  | | | |  |  |
|  |  |  | | | |  |  |
|  |  |  |  | |  | Being able or ready to parent | 4 [4, 6-8] |
|  |  |  | | | | Expectations versus reality | 1 [7]  6 [2, 3, 6, 11, 19, 22]  3 [1, 11, 17] |
|  |  |  | |  | | Identity |  |
|  |  |  | | | | Containing trauma and ‘being good’ |  |
|  | *2.3 Wanting to be ‘normal’* |  | | | | Wanting to be ‘normal’ | 3 [6, 11, 22] |
|  | *2.4 Knowledge and learning about parenting* |  | | | | Lack of knowledge  Learning about pregnancy and parenting  Parent skill training  Access to information  Reassurance | 3 [2, 11, 23]  4 [2, 3, 7, 14]  2 [5, 8]  2 [2, 24]  1 [11] |
|  |  |  | | | |  |  |
|  |  |  | | | |  |  |
|  |  |  | | | |  |  |
| 3. *Feeling connected*: The quality of relationships with self, baby and others has major impacts on the experiences of becoming a parent. | *3.1 New experiences of love and joy* |  | | | | Love or joy  Primacy of the baby’s needs above all else | 7 [2, 4, 6-8, 10, 18]  1 [11] |
|  |  |  | | | |  |  |
|  | *3.2 Relationship with self/body* |  | | | | Body in pregnancy and birth (challenges)  Body in pregnancy and birth (positive experiences)  Co-morbidity or illnesses in pregnancy or physical symptoms  Baby as feeling foreign and invading their body  Sexual traumatisation  Fertility issues  Conflict between being ‘spoiled’ versus wanting to be ‘pure’ | 8 [2-4, 6, 11, 16, 19, 25]  6 [2, 3, 6, 18, 19, 22]  3 [3, 4, 25]  3 [2, 11, 22]  5 [2, 3, 6, 16, 19]  5 [2, 3, 6-8]  2 [2, 8] |
|  |  |  | | | |  |  |
|  |  |  | | | |  |  |
|  |  |  | | | |  |  |
|  |  |  | | | |  |  |
|  |  |  | | | |  |  |
|  |  |  | | | |  |  |
|  | *3.3* *Relationship with child and bonding* |  | | | | Bonding (positive experiences)  Early attachment (challenges)  Breastfeeding positive experiences  Breastfeeding challenges  Bonding or attachment through breastfeeding  Dissociation during breastfeeding  Baby gender  Parent-child relationship | 3 [6, 7, 23]  7 [2, 6, 7, 11, 15, 16, 23]  2 [19, 21]  9 [2, 6, 7, 16, 18, 19, 21, 22, 24]  2 [6, 21]  1 [24]  2 [9, 18]  2 [18, 25] |
|  |  |  | | | |  |  |
|  |  |  | | | |  |  |
|  |  |  | | | |  |  |
|  |  |  | | | |  |  |
|  |  |  | | | |  |  |
|  |  |  | | | |  |  |
|  |  |  | | | |  |  |
|  | *3.4 Relationship with partner (including IPV)* |  | | | | Positive relationship with their partner  Partner support  Challenging partner relationships  IPV in pregnancy  Partners’ family | 5 [2, 8-10, 14]  2 [3, 8]  9 [1, 2, 4, 6-10, 15]  2 [13, 26]  1 [4] |
|  |  |  | | | |  |  |
|  |  |  | | | |  |  |
|  |  |  | | | |  |  |
|  |  |  | | | |  |  |
|  | *3.5 Relationship with family of origin* |  | |  | | Family of origin | 8 [3, 4, 6-8, 10, 13, 18] |
|  | 3.6 *Other relationships and support* |  | | | | Supportive relationships  Support people during birth  Need for support  Lack of support during pregnancy  Lack of support in early postpartum  Friends | 3 [2, 4, 6]  2 [4, 10]  3 [6, 11, 15]  5 [2, 6, 9, 12, 15]  2 [6, 23]  1 [6] |
|  |  |  | | | |  |  |
|  |  |  | | | |  |  |
|  |  |  | | | |  |  |
|  |  |  | | | |  |  |
|  |  |  | | | |  |  |
| 4. *Compassionate care*: Kindness, empathy and sensitivity enables parents to build trust and feel valued and cared for. | *4.1 Provider support, communication and relationships* |  | | | | Perinatal care experience  Supportive providers  ‘Normalising’  Let others in  Trust in care providers  Lack of care, empathy or understanding  Inadequate care  Poor provider communication  Being disregarded or depersonalised  Care being reminiscent of abuse  Re-experiencing or being triggered in care setting  Invasive and violating  Effective communication in the care setting  Respect, empathy and understanding  Personalised individualised care  Lack of continuity of care  Continuity of care | 1 [13]  3 [2, 5, 16]  1 [19]  1 [2]  7 [2, 11, 16, 20, 22, 23, 26]  3 [2, 16, 22]  1 [2]  5 [2, 6, 11, 16, 19]  6 [2, 6, 11, 16, 20, 22]  4 [17, 19, 21, 22]  8 [2, 6, 16, 17, 20, 22, 24, 27]  6 [2, 6, 16, 20, 22, 23]  7 [3, 5, 16, 21, 26-28]  2 [6, 16]  3 [11, 16, 21]  1 [5]  6 [6, 16, 20, 23, 27, 29] |
|  |  |  | | | |  |  |
|  |  |  | | | |  |  |
|  |  |  | | | |  |  |
|  |  |  | | | |  |  |
|  |  |  | | | |  |  |
|  |  |  | | | |  |  |
|  |  |  | | | |  |  |
|  |  |  | | | |  |  |
|  |  |  | | | |  |  |
|  |  |  | | | |  |  |
|  |  |  | | | |  |  |
|  |  |  | | | |  |  |
|  |  |  | | | |  |  |
|  |  |  | | | |  |  |
|  |  |  | | | |  |  |
|  |  |  | | | |  |  |
|  | *4.2 Trauma-informed care and factors which foster safety and enable care* |  | | | | Postpartum access to care  Others’ expectations  ‘Trauma-informed’ perinatal care  Positive strengths-based approaches  Multi-disciplinary care and collaboration  Improving professional contact opportunities | 1 [20]  1 [6]  10 [2, 3, 5, 6, 11, 19, 20, 22, 24, 26].  1 [5]  1 [5]  1 [20] |
|  |  |  | | | |  |  |
|  |  |  | | | |  |  |
|  |  |  | | | |  |  |
|  |  |  | | | |  |  |
|  |  |  | | | |  |  |
|  | *4.3 Experiences of care during birth and breastfeeding* |  | | | | Birth (positive experiences)  Birth (challenges)  Healing process of birth  Support people  Birth partner  Hopes for the subsequent birth  Traumatic birth experiences  Stillbirth  Pregnancy loss  Breastfeeding challenges in the care setting | 4 [2-4, 16]  4 [2, 4, 19, 23]  2 [3, 6]  2 [4, 10]  4 [2, 11, 16, 23]  2 [2, 3]  3 [2, 4, 6]  1 [2]  1 [2]  6 [2, 6, 16, 21, 24, 29] |
|  |  |  | | | |  |  |
|  |  |  | | | |  |  |
|  |  |  | | | |  |  |
|  |  |  | | | |  |  |
|  |  |  | | | |  |  |
|  |  |  | | | |  |  |
|  |  |  | | | |  |  |
|  |  |  | | | |  |  |
|  |  |  | | | |  |  |
| 5. *Empowerment*: Control, choice and ‘having a voice’ are critical to fostering safety. | *5.1 Empowerment, choice and control* |  | | | | Lack of control  Vulnerability  Shame/humiliation  Authority | 11 [2, 6, 11, 15-17, 19, 20, 22, 23, 25]  2 [11, 22]  1 [16]  8 [2, 6, 16, 18, 20, 22-24]  7 [5, 6, 11, 16, 22, 26, 27]  4 [2, 3, 6, 23]  10 [3, 6, 11, 15, 16, 22, 23, 26, 27, 30]  2 [3, 6]  1 [16] |
|  |  |  | | | |  |  |
|  |  |  | | | |  |  |
|  |  |  | | | |  |  |
|  |  |  | |  | | Care provider gender |  |
|  |  |  | | | | Empowerment, choice and control (positive birth experiences)  Empowerment, choice and control (in improving care)  Having a birth plan  Home birth |  |
|  |  |  | | | |  |  |
|  |  |  | | | |  |  |
|  |  |  | | | |  |  |
|  | *5.2 Having a voice* |  | | | | Talking about trauma  Talking about birth experiences  Asking about trauma  Expressing trauma  Communication | 2 [3, 22]  1 [6]  3 [2, 26, 28]  4 [2, 3, 6, 9]  1 [7] |
|  |  |  | | | |  |  |
|  |  |  | | | |  |  |
|  |  |  | | | |  |  |
|  |  |  | | | |  |  |
|  | *5.3 Disclosure of abuse history* |  | | | | Disclosure of abuse (positive experiences)  Disclosure of abuse (challenges)  Impact on family  Fear of losing child  Fear of not being believed  Not wanting to burden others  Wanting to appear normal | 2 [3, 6]  8 [2, 3, 6, 11, 16, 19, 22, 26]  1 [16]  2 [19, 22]  1 [19].  1 [22]  2 [6, 22] |
|  |  |  | | | |  |  |
|  |  |  | | | |  |  |
|  |  |  | | | |  |  |
|  |  |  | | | |  |  |
|  |  |  | | | |  |  |
|  |  |  | | | |  |  |
| 6. *Creating safety*: Parents perceive the ‘world as unsafe’ and use conscious strategies to build safe places and relationships to protect themselves and their baby. | *6.1 The world is unsafe and strategies to protect themselves and their baby* |  | | | | Safety in disclosing their abuse history  Safety or protecting themselves and their baby  Monitoring safety and hypervigilance  Trusting others  Creating an environment of trust  Building trusting relationships with care providers  Regaining a sense of safety  Child safety and protection  Safety in perinatal care  Environments that foster safety  Protecting the child or ensuring safety | 2 [11, 26]  7 [1, 3, 6, 9, 11, 18, 20]  3 [2, 11, 20]  3 [9, 16, 18]  2 [7, 11]  2 [5, 27]  2 [6, 19]  4 [1, 2, 5, 7]  3 [11, 20, 23]  1 [5]  2 [2, 7] |
|  |  |  | | | |  |  |
|  |  |  | | | |  |  |
|  |  |  | | | |  |  |
|  |  |  | | | |  |  |
|  |  |  | | | |  |  |
|  |  |  | | | |  |  |
|  |  |  | | | |  |  |
|  |  |  | | | |  |  |
|  |  |  | | | |  |  |
|  |  |  | | | |  |  |
|  | *6.2 The external world around them* |  | | | | External environment  Housing instability  Financial challenges  Stigma or judgement  Racism and discrimination  Education or employment (opportunities)  Education or employment (challenges)  Practical support  Foster care | 1 [8]  3 [4, 8, 13]  3 [2, 8, 14]  7 [3, 4, 6-8, 16, 22]  1 [8]  2 [2, 8]  2 [4, 8]  1 [8]  2 [8, 13] |
|  |  |  | | | |  |  |
|  |  |  | | | |  |  |
|  |  |  | | | |  |  |
|  |  |  | | | |  |  |
|  |  |  | | | |  |  |
|  |  |  | | | |  |  |
|  |  |  | | | |  |  |
|  |  |  | | | |  |  |
| 7. *‘Reweaving’ a future*: Managing distress and healing while becoming a parent is a personal ongoing and complex process requiring strength, hope and support. | *7.1 Distress symptoms, including fear and lack of trust* |  | | | | Symptom reduction  Distress symptoms  Fear during pregnancy  Fear in care  Shame or humiliation in care  Negative self-belief  Guilt  Anxiety and depression  Postpartum depression  Avoidance or denial  Avoidance of care  Numbing  Re-experiencing  Triggers  Nightmares  Intrusive thoughts or nightmares  Delayed memory  Dissociation  Passivity  Substance use in pregnancy | 1 [3]  13 [1-4, 6, 8, 9, 15, 16, 22, 23, 25, 30]  4 [2, 4, 6, 16]  5 [2, 6, 16, 17, 22]  2 [6, 16]  6 [5, 6, 8, 9, 11, 16]  3 [11, 20, 22]  3 [2, 6, 8]  3 [6, 15, 18]  6 [6, 10, 11, 16, 25, 26]  2 [6, 11]  3 [11, 16, 26]  10 [2, 3, 6, 9, 11, 18, 23, 25, 27, 30]  3 [6, 16, 23]  2 [6, 11]  3 [2, 3, 16]  3 [17, 23, 30]  7 [3, 6, 16, 23-26]  1 [11]  6 [2, 8, 13, 25-27] |
|  |  |  | | | |  |  |
|  |  |  | | | |  |  |
|  |  |  | | | |  |  |
|  |  |  | | | |  |  |
|  |  |  | | | |  |  |
|  |  |  | | | |  |  |
|  |  |  | | | |  |  |
|  |  |  | | | |  |  |
|  |  |  | | | |  |  |
|  |  |  | | | |  |  |
|  |  |  | | | |  |  |
|  |  |  | | | |  |  |
|  |  |  | | | |  |  |
|  |  |  | | | |  |  |
|  |  |  | | | |  |  |
|  |  |  | | | |  |  |
|  |  |  | | | |  |  |
|  |  |  | | | |  |  |
|  |  |  | | | |  |  |
|  |  |  | | | |  |  |
|  | *7.2 Coping strategies* |  | | | | Coping strategies  Managing, containing or controlling trauma  Coping strategies during vaginal examinations  Coping strategies during birth  Establishing healthy boundaries or balance  Spirituality or faith  Self-help or care strategies  Reading | 2 [3, 6]  1 [1]  1 [20]  3 [6, 11, 23]  1 [6]  1 [8]  3 [3, 6, 8]  1 [2] |
|  | *7.3 Factors that help recovery, growth and healing* |  | | | | Making sense of trauma  ‘Survivor’ and ‘victim’  Understandings of trauma  Catalyst to seek support  Clinical therapy  Ambivalence in seeking help  Peer support  Parenting groups  Trauma groups  Non-clinical therapies  Art therapy  Bodywork  Allowing time  Acceptance or forgiveness  Helping others | 5 [1, 3, 6, 7, 20]  1 [11]  1 [26]  2 [3, 5]  6 [1, 5-7, 11, 27]  1 [5]  2 [5, 28]  4 [2, 5, 18, 29]  2 [2, 6]  2 [3, 5]  1 [27]  1 [3]  1 [11]  1 [3]  2 [3, 8] |
|  |  |  | | | |  |  |
|  |  |  | | | |  |  |
|  |  |  | | | |  |  |
|  |  |  | | | |  |  |
|  |  |  | | | |  |  |
|  |  |  | | | |  |  |
|  |  |  | | | |  |  |
|  |  |  | | | |  |  |
|  |  |  | | | |  |  |
|  |  |  | | | |  |  |
|  |  |  | | | |  |  |
|  |  |  | | | |  |  |
|  |  |  | | | |  |  |
|  | *7.4 The healing process of pregnancy, birth and parenting* |  | | | | Healing through becoming a parent  Recovery  Post-traumatic growth  Resilience or being strong  Survival fostering resilience and agency | 9 [2, 3, 5, 6, 8-10, 19, 24]  2 [9, 27]  2 [9, 26]  3 [4, 6, 27]  2 [2, 18] |
|  |  |  | | | |  |  |
|  |  |  | | | |  |  |
|  |  |  | | | |  |  |
|  |  |  | | | |  |  |
|  |  |  | | | |  |  |
| Providers views |  |  | | | | No major additional insights  Barriers to being present in a professional role  Being seen as a worker and not a parent  Care can be reminiscent of abuse  Conventional practice is not trauma-informed  Choice and control, good communication, treating women as individuals  Acting as advocates on women's behalf | 1 [8]  1 [16] |

**References**

1. Berman H, Mason R, Hall J, Rodger S, Classen CC, Evans MK, et al. Laboring to mother in the context of past trauma: the transition to motherhood. Qual Health Res. 2014;24(9):1253-64. doi: 10.1177/1049732314521902.

2. Lasiuk GC. The lived experience of pregnancy and birthing of women with histories of childhood sexual abuse. Canada: University of Alberta; 2007.

3. Lee SRC. Survivors of childhood sexual abuse and the childbearing year. Dissertation Abstracts International Section B: The Sciences and Engineering. 2001;62(4-B):2064.

4. McCoy JJ. Maternal perceptions and pregnancy experiences of former foster youth with histories of sexual abuse. Dissertation Abstracts International Section B: The Sciences and Engineering. 2015;76(4-B(E)).

5. Muzik M, Ads M, Bonham C, Lisa Rosenblum K, Broderick A, Kirk R. Perspectives on trauma-informed care from mothers with a history of childhood maltreatment: A qualitative study. Child Abuse Negl. 2013;37(12):1215-24. doi: 10.1016/j.chiabu.2013.07.014.

6. Palmer BC. The childbearing experience of women who are childhood sexual abuse survivors. Canada: University of British Columbia; 2005.

7. Roberts RE. The survivors of child maltreatment description of the process of becoming a parent: A grounded theory study. Dissertation Abstracts International Section B: The Sciences and Engineering. 2011;72(6-B):3765.

8. Saewyc EM. Meanings of pregnancy and motherhood among out-of-home pregnant adolescents. Dissertation Abstracts International Section B: The Sciences and Engineering. 2000;60(11-B):5437.

9. Schwerdtfeger KL, Wampler KS. Sexual trauma and pregnancy: A qualitative exploration of women's dual life experience. Contemp Fam Ther. 2009;31(2):100-22. doi: 10.1007/s10591-009-9083-9.

10. Williams C, Vines SW. Broken past, fragile future: Personal stories of high-risk adolescent mothers. J Soc Pediatr Nurs. 1999;4(1):15-23.

11. Richmond KK. Being whole: Aligning personhoods to achieve successful childbirth with a history of childhood sexual abuse during perinatal services. United States of America: University of San Diego; 2006.

12. Kennedy AC, Agbényiga DL, Kasiborski N, Gladden J. Risk chains over the life course among homeless urban adolescent mothers: altering their trajectories through formal support. Child Youth Serv Rev. 2010;32(12):1740-9.

13. Miura PO, Tardivo L, Barrientos DMS. Helplessness experienced by adolescent mothers and pregnant adolescents sheltered in institutions. Cien Saude Colet. 2018;23(5):1601-10. doi: 10.1590/1413-81232018235.14152016.

14. Swartz NE, Mercier DJ, Curran MA. Influences of childhood abuse on parenting perspectives of pregnant cohabitors. J Fam Violence. 2012;27(6):597-606. doi: 10.1007/s10896-012-9452-2.

15. O'Brien DW. A qualitative study of parenting by incest survivors. Dissertation Abstracts International Section A: Humanities and Social Sciences. 1999;59(7-A):2721.

16. Garratt EF. The childbearing experiences of survivors of childhood sexual abuse. Dissertation Abstracts International Section C: Worldwide. 2018;75(4-C).

17. Montgomery E, Pope C, Rogers J. The re-enactment of childhood sexual abuse in maternity care: A qualitative study. BMC Pregnancy Childbirth. 2015;15:194. doi: 10.1186/s12884-015-0626-9.

18. Cohen T. Experiences of motherhood among women who were victims of childhood incest. Dissertation Abstracts International Section B: Sciences and Engineering. 1987;48(4-B):1148.

19. Byrne J, Smart C, Watson G. "I felt like i was being abused all over again": How survivors of child sexual abuse make sense of the perinatal period through their narratives. J Child Sex Abus. 2017;26(4):465-86. doi: 10.1080/10538712.2017.1297880.

20. Coles J, Jones K. "Universal Precautions": Perinatal touch and examination after childhood sexual abuse. Birth. 2009;36(3):230-6. doi: 10.1111/j.1523-536X.2009.00327.x.

21. Coles J. Qualitative study of breastfeeding after childhood sexual assault. J Hum Lact. 2009;25(3):317-24. doi: 10.1177/0890334409334926.

22. Montgomery E, Pope C, Rogers J. A feminist narrative study of the maternity care experiences of women who were sexually abused in childhood. Midwifery. 2015;31(1):54-60. doi: 10.1016/j.midw.2014.05.010.

23. Parratt J. The experience of childbirth for survivors of incest. Midwifery. 1994;10(1):26-39.

24. Wood K, Van Esterik P. Infant feeding experiences of women who were sexually abused in childhood. Can Fam Physician. 2010;56(4):e136-41.

25. Seng JS, Low LK, Sparbel KJ, Killion C. Abuse-related post-traumatic stress during the childbearing year. J Adv Nurs. 2004;46(6):604-13.

26. Seng JS, Sparbel KJ, Low LK, Killion C. Abuse-related posttraumatic stress and desired maternity care practices: Women's perspectives. J Midwifery Womens Health. 2002;47(5):360-70.

27. Roller CG. Moving beyond the pain: Women's responses to the perinatal period after childhood sexual abuse. J Midwifery Womens Health. 2011;56(5):488-93. doi: 10.1111/j.1542-2011.2011.00051.x.

28. White A, Danis M, Gillece J. Abuse survivor perspectives on trauma inquiry in obstetrical practice. Arch Womens Ment Health. 2016;19(2):423-7. doi: 10.1007/s00737-015-0547-7.

29. Datta J, Macdonald G, Barlow J, Barnes J, Elbourne D. Challenges faced by young mothers with a care history and views of stakeholders about the potential for Group Family Nurse Partnership to support their needs. Child Soc. 2017;31(463-474).

30. Rhodes N, Hutchinson S. Labor experiences of childhood sexual abuse survivors. Birth. 1994;21(4):213-20.
