# Supplementary material for: Healing The Past By Nurturing The Future: A qualitative systematic review and meta-synthesis of pregnancy, birth and early postpartum experiences and views of parents with a history of childhood maltreatment
Source: PLoS One. 2019 Dec 13;14(12):e0225441. doi: 10.1371/journal.pone.0225441 (PMC6910698; doi:10.1371/journal.pone.0225441)
Supplement: S6 Appendix — Summary table considering review’s analytic themes and descriptive subthemes by selected study characteristics. (DOCX) [file pone.0225441.s006.docx]

## **S6 Appendix: Summary of assessment of analytic themes and descriptive subthemes by selected study characteristics**

| **Analytic theme** | **Descriptive subtheme** | **No. of articles contributing to subtheme** | **No. of articles with low reflexivity (15 in total) contributing to subtheme** | **No. of articles including fathers (2 in total) contributing to subtheme** | **No. of articles of young parents (6 in total) contributing to subtheme** | **No. of articles with a majority of parents from ethnic minority groups (5 in total) contributing to subtheme** | **No. of articles with a majority of single parents (8 in total) contributing to subtheme** | **No. of articles of parents with CSA and other forms of CM (9 in total) contributing to subtheme** |
| --- | --- | --- | --- | --- | --- | --- | --- | --- |
| 1. *New beginnings*: Becoming a parent is an opportunity for ‘a fresh start’, to put the past behind them and move forward with hope for the future to create a new life for themselves and their child. | *1.1 New opportunities and motivations for change* | 11 [1-11] | 3 [5, 9, 10] | 1 [7] | 2 [8, 10] | 1 [11] | 3 [1, 6, 10] | 6 [1, 5, 7-10] |
|  | *1.2 Hopes and dreams for the future* | 12 [1-10, 12, 13] | 5 [5, 9, 10, 12, 14] | 1 [7] | 4 [4, 8, 10, 12] | 2 [4, 12] | 4 [1, 6, 10, 12] | 7 [1, 5, 7-10, 12] |
|  | *1.3 Wanting to parent differently* | 12 [1, 2, 4-9, 11, 15-17] | 4 [5, 9, 16, 17] | 2 [7, 16] | 3 [4, 8, 15] | 2 [4, 11] | 4 [6, 15, 17] | 7 [1, 5, 7-9, 15, 16] |
| 2. *Changing roles and identities*: Becoming a parent is a major life transition, influenced by perceptions of the parenting role. | 2.1 *Mixed emotions in pregnancy and birth* | 10 [1, 2, 4, 6-8, 17-20] | 2 [17, 19] | 1 [7] | 2 [4, 8] | 1 [4] | 3 [1, 6, 17] | 3 [1, 7, 8] |
|  | *2.2 Striving to be a ‘good’ or ‘perfect’ parent* | 15 [1-8, 11, 13, 19-23] | 3 [13, 19, 22] | 1 [7] | 2 [4, 8] | 2 [4, 11] | 2 [1, 6] | 3 [1, 7, 8] |
|  | *2.3 Wanting to be ‘normal’* | 3 [6, 11, 23] | 1 [23] | N/A | N/A | 1 [11] | 1 [6] | N/A |
|  | *2.4 Knowledge and learning about parenting* | 9 [2, 3, 5, 7, 8, 11, 14, 16, 24] | 4 [5, 14, 16, 24] | 2 [7, 16] | 1 [8] | 1 [11] | N/A | 4 [5, 7, 8, 16] |
| 3. *Feeling connected*: The quality of relationships with self, baby and others has major impacts on the experiences of becoming a parent. | *3.1 New experiences of love and joy* | 8 [2, 4, 6-8, 10, 11, 20] | 1 [10] | 1 [7] | 3 [4, 8, 10] | 2 [4, 11] | 2 [6, 10] | 3 [7, 8, 10] |
|  | *3.2 Relationship with self/body* | 12 [2-4, 6-8, 11, 18-21, 25] | 1 [23] | 1 [7] | 2 [4, 8] | 2 [4, 11] | 1 [6] | 2 [7, 8] |
|  | *3.3* *Relationship with child and bonding* | 14 [2, 6, 7, 9, 11, 13, 14, 17-21, 24, 25] | 6 [9, 13, 14, 17, 23, 24] | 1 [7] | N/A | 1 [11] | 2 [6, 17] | 2 [7, 9] |
|  | *3.4 Relationship with partner (including IPV)* | 13 [1-4, 6-10, 15-17, 26] | 4 [9, 10, 16, 17] | 2 [7, 16] | 4 [4, 8, 10, 15] | 1 [4] | 5 [1, 6, 10, 15, 17] | 7 [1, 7-10, 15, 16] |
|  | *3.5 Relationship with family of origin* | 10 [2-4, 6-8, 10, 15, 20, 21] | 1 [10] | 1 [7] | 4 [4, 8, 10, 15] | 1 [4] | 3 [6, 10, 15] | 4 [7, 8, 10, 15] |
|  | 3.6 *Other relationships and support* | 9 [2, 4, 6, 9-12, 14, 17] | 5 [9, 10, 12, 14, 17] | N/A | 3 [4, 10, 12] | 3 [4, 11, 12] | 4 [6, 10, 12, 17] | 3 [9, 10, 12] |
| 4. *Compassionate care*: Kindness, empathy and sensitivity enables parents to build trust and feel valued and cared for. | *4.1 Provider support, communication and relationships* | 18 [2, 3, 5, 6, 11, 13-15, 18, 19, 21-24, 26-29] | 9 [5, 13, 14, 19, 22-24, 27, 29] | N/A | 1 [15] | 3 [11, 27, 28] | 4 [6, 15, 27, 29] | 3 [5, 15, 28] |
|  | *4.2 Trauma-informed care and factors which foster safety and enable care* | 10 [2, 3, 5, 6, 11, 19, 21, 22, 24, 26] | 4 [5, 22-24] | N/A | N/A | 1 [11] | 1 [6] | 1 [5] |
|  | *4.3 Experiences of care during birth and breastfeeding* | 12 [2-4, 6, 10, 11, 13, 14, 18, 21, 24, 29] | 5 [10, 13, 14, 24, 29] | N/A | 3 [4, 10, 29] | 2 [4, 11] | 2 [6, 29] | 2 [10, 29] |
| 5. *Empowerment*: Control, choice and ‘having a voice’ are critical to fostering safety. | *5.1 Empowerment, choice and control* | 18 [2, 3, 5, 6, 11, 14, 17-27, 30] | 8 [5, 14, 17, 19, 22, 23, 27, 30] | N/A | N/A | 2 [11, 27] | 3 [6, 17, 27] | 1 [5] |
|  | *5.2 Having a voice* | 8 [2, 3, 6, 7, 9, 19, 26, 28] | 2 [9, 23] | 1 [7] | N/A | N/A | 1 [6] | 1 [28] |
|  | *5.3 Disclosure of abuse history* | 9 [2, 3, 5, 6, 11, 18, 19, 21, 26] | [23] | N/A | N/A | 1 [11] | 1 [6] | N/A |
| 6. *Creating safety*: Parents perceive the ‘world as unsafe’ and use conscious strategies to build safe places and relationships to protect themselves and their baby. | *6.1 The world is unsafe and strategies to protect themselves and their baby* | 15 [1-3, 5-7, 9, 11, 14, 18, 20-22, 26, 27] | 5 [5, 9, 14, 22, 27] | 1 [7] | N/A | 2 [11, 27] | 3 [1, 6, 27] | 4 [1, 5, 7, 9] |
|  | *6.2 The external world around them* | 10 [2-4, 6-8, 15, 16, 18, 19] | 2 [16, 23] | 2 [7, 16] | 4 [4, 8, 10, 15] | 1 [4] | 2 [6, 15] | 4 [7, 8, 15, 16] |
| 7. *‘Reweaving’ a future*: Managing distress and healing while becoming a parent is a personal ongoing and complex process requiring strength, hope and support. | *7.1 Distress symptoms, including fear and lack of trust* | 23 [1-6, 8-11, 14, 15, 17-20, 22-27, 30] | 10 [5, 9, 10, 14, 19, 22-24, 27, 30] | N/A | 4 [4, 8, 10, 15] | 3 [4, 11, 27] | 5 [1, 6, 10, 17, 27] | 5 [5, 8-10, 15] |
|  | *7.2 Coping strategies* | 8 [1-3, 6, 8, 11, 14, 22] | 2 [14, 22] | N/A | 1 [8] | 1 [11] | 2 [1, 6] | 2 [1, 8] |
|  | *7.3 Factors that help recovery, growth and healing* | 14 [1-3, 5-8, 11, 20, 22, 26-29] | 4 [5, 22, 27, 29] | 1 [7] | 2 [8, 29] | 3 [11, 27, 28] | 4 [1, 6, 27, 29] | 6 [1, 5, 7, 8, 28, 29] |
|  | *7.4 The healing process of pregnancy, birth and parenting* | 13 [2-6, 8-10, 20, 21, 24, 26, 27] | 5 [5, 9, 10, 24, 27] | N/A | 3 [4, 8, 10] | 2 [4, 27] | 3 [6, 10, 27] | 4 [5, 8-10] |

# **References**

1. Berman H, Mason R, Hall J, Rodger S, Classen CC, Evans MK, et al. Laboring to mother in the context of past trauma: the transition to motherhood. Qual Health Res. 2014;24(9):1253-64. doi: 10.1177/1049732314521902.

2. Lasiuk GC. The lived experience of pregnancy and birthing of women with histories of childhood sexual abuse. Canada: University of Alberta; 2007.

3. Lee SRC. Survivors of childhood sexual abuse and the childbearing year. Dissertation Abstracts International Section B: The Sciences and Engineering. 2001;62(4-B):2064.

4. McCoy JJ. Maternal perceptions and pregnancy experiences of former foster youth with histories of sexual abuse. Dissertation Abstracts International Section B: The Sciences and Engineering. 2015;76(4-B(E)).

5. Muzik M, Ads M, Bonham C, Lisa Rosenblum K, Broderick A, Kirk R. Perspectives on trauma-informed care from mothers with a history of childhood maltreatment: A qualitative study. Child Abuse Negl. 2013;37(12):1215-24. doi: 10.1016/j.chiabu.2013.07.014.

6. Palmer BC. The childbearing experience of women who are childhood sexual abuse survivors. Canada: University of British Columbia; 2005.

7. Roberts RE. The survivors of child maltreatment description of the process of becoming a parent: A grounded theory study. Dissertation Abstracts International Section B: The Sciences and Engineering. 2011;72(6-B):3765.

8. Saewyc EM. Meanings of pregnancy and motherhood among out-of-home pregnant adolescents. Dissertation Abstracts International Section B: The Sciences and Engineering. 2000;60(11-B):5437.

9. Schwerdtfeger KL, Wampler KS. Sexual trauma and pregnancy: A qualitative exploration of women's dual life experience. Contemp Fam Ther. 2009;31(2):100-22. doi: 10.1007/s10591-009-9083-9.

10. Williams C, Vines SW. Broken past, fragile future: Personal stories of high-risk adolescent mothers. J Soc Pediatr Nurs. 1999;4(1):15-23.

11. Richmond KK. Being whole: Aligning personhoods to achieve successful childbirth with a history of childhood sexual abuse during perinatal services. United States of America: University of San Diego; 2006.

12. Kennedy AC, Agbényiga DL, Kasiborski N, Gladden J. Risk chains over the life course among homeless urban adolescent mothers: altering their trajectories through formal support. Child Youth Serv Rev. 2010;32(12):1740-9.

13. Coles J. Qualitative study of breastfeeding after childhood sexual assault. J Hum Lact. 2009;25(3):317-24. doi: 10.1177/0890334409334926.

14. Parratt J. The experience of childbirth for survivors of incest. Midwifery. 1994;10(1):26-39.

15. Miura PO, Tardivo L, Barrientos DMS. Helplessness experienced by adolescent mothers and pregnant adolescents sheltered in institutions. Cien Saude Colet. 2018;23(5):1601-10. doi: 10.1590/1413-81232018235.14152016.

16. Swartz NE, Mercier DJ, Curran MA. Influences of childhood abuse on parenting perspectives of pregnant cohabitors. J Fam Violence. 2012;27(6):597-606. doi: 10.1007/s10896-012-9452-2.

17. O'Brien DW. A qualitative study of parenting by incest survivors. Dissertation Abstracts International Section A: Humanities and Social Sciences. 1999;59(7-A):2721.

18. Garratt EF. The childbearing experiences of survivors of childhood sexual abuse. Dissertation Abstracts International Section C: Worldwide. 2018;75(4-C).

19. Montgomery E, Pope C, Rogers J. The re-enactment of childhood sexual abuse in maternity care: A qualitative study. BMC Pregnancy Childbirth. 2015;15:194. doi: 10.1186/s12884-015-0626-9.

20. Cohen T. Experiences of motherhood among women who were victims of childhood incest. Dissertation Abstracts International Section B: Sciences and Engineering. 1987;48(4-B):1148.

21. Byrne J, Smart C, Watson G. "I felt like i was being abused all over again": How survivors of child sexual abuse make sense of the perinatal period through their narratives. J Child Sex Abus. 2017;26(4):465-86. doi: 10.1080/10538712.2017.1297880.

22. Coles J, Jones K. "Universal Precautions": Perinatal touch and examination after childhood sexual abuse. Birth. 2009;36(3):230-6. doi: 10.1111/j.1523-536X.2009.00327.x.

23. Montgomery E, Pope C, Rogers J. A feminist narrative study of the maternity care experiences of women who were sexually abused in childhood. Midwifery. 2015;31(1):54-60. doi: 10.1016/j.midw.2014.05.010.

24. Wood K, Van Esterik P. Infant feeding experiences of women who were sexually abused in childhood. Can Fam Physician. 2010;56(4):e136-41.

25. Seng JS, Low LK, Sparbel KJ, Killion C. Abuse-related post-traumatic stress during the childbearing year. J Adv Nurs. 2004;46(6):604-13.

26. Seng JS, Sparbel KJ, Low LK, Killion C. Abuse-related posttraumatic stress and desired maternity care practices: Women's perspectives. J Midwifery Womens Health. 2002;47(5):360-70.

27. Roller CG. Moving beyond the pain: Women's responses to the perinatal period after childhood sexual abuse. J Midwifery Womens Health. 2011;56(5):488-93. doi: 10.1111/j.1542-2011.2011.00051.x.

28. White A, Danis M, Gillece J. Abuse survivor perspectives on trauma inquiry in obstetrical practice. Arch Womens Ment Health. 2016;19(2):423-7. doi: 10.1007/s00737-015-0547-7.

29. Datta J, Macdonald G, Barlow J, Barnes J, Elbourne D. Challenges faced by young mothers with a care history and views of stakeholders about the potential for Group Family Nurse Partnership to support their needs. Child Soc. 2017;31(463-474).

30. Rhodes N, Hutchinson S. Labor experiences of childhood sexual abuse survivors. Birth. 1994;21(4):213-20.
