# Supplementary material for: Healing The Past By Nurturing The Future: A qualitative systematic review and meta-synthesis of pregnancy, birth and early postpartum experiences and views of parents with a history of childhood maltreatment
Source: PLoS One. 2019 Dec 13;14(12):e0225441. doi: 10.1371/journal.pone.0225441 (PMC6910698; doi:10.1371/journal.pone.0225441)
Supplement: S7 Appendix — Table of excluded studies, with reasons for exclusion provided. (DOCX) [file pone.0225441.s007.docx]

**S7 Appendix: Table of excluded studies**

| **Study ID** | **Reasons for exclusion** | **Study description** |
| --- | --- | --- |
| Abboud 2005 [1] | Excluded on basis of relevance to topic (focussed exclusively on miscarriage) and not on parenting or other aspects of perinatal care. | Qualitative study of experiences of 6 women and their partners with ethnic backgrounds about miscarriage |
| Abraham 2018 [2] | Excluded due to age and topic, with focus on survivor meaning making in relation to religion. | Meaning making for the treatment of complex trauma for 13 adult survivors and their mechanisms for change |
| Abram 2008 [3] | Excluded due to lack of CM background (not identified as an inclusion criteria). | Qualitative study of experiences, impact and meaning about pregnancy and early parenting for 11 adult women. |
| Aho 2014 [4] | Excluded due to lack of children, and perinatal topic focus. | The stories of multi-generational historical trauma for Maori women in New Zealand and how telling the stories assisted healing. |
| Allbaugh 2014 [5] | Excluded due to study design (quantitative); and not perinatal period. | Factor analysis and validation of a parenting questionnaire (Ruscio) of 60 child sexual abuse mothers. |
| Allen 2010 [6] | Excluded due to CM backgrounds and age of children (not perinatal period). | Using a feminist standpoint epistemological framework, this article reports the findings from 26 interviews with drug-offending incarcerated mothers in Kentucky regarding the effects of incarceration on their relationships with their children. |
| Allnock 2013 [7] | Excluded due to lack of specified parent sample, nor perinatal care. | The report describes the childhood experiences of abuse of 60 young men and women and how they disclosed this abuse and sought help. |
| Almqvist 2003 [8] | Excluded due to study design (case study) and lack of CM background (trauma background of parent not established). | Three examples of young children traumatized by organized violence together with their mothers – The critical effects of damaged internal representations |
| Amin 2017 [9] | Excluded due to topic (i.e. physicians perceived role responding to sexual abuse in practise, and no other obstetric or perinatal areas); and not about parents in perinatal period. | Qualitative study of 16 physicians about their experiences (enablers and challenges) of treating and supporting child sexual abuse survivors. |
| Anderson 2006 [10] | Excluded due to age of sample, and topic (i.e. youth in Hawaii’s drug and alcohol resistance strategies); and not about parenting. | Pilot study of the gender differences of 74 (in focus groups) native Hawaiian youth and their drug and alcohol resistant strategies. |
| Andrews 2002 [11] | Excluded due to age of children; focus of topic on life course and not parenting or perinatal; and retrospective study from elderly subjects on their parenting. | Retrospective study of interviews with 4 elderly adults talking about their relationship with their mother and their life course. |
| Annand 2018 [12] | Excluded due to lack of CM background. | Qualitative interviews with 9 mothers, previously court-mandated, to attend substance abuse programs (by child welfare services) to discuss their pre and perinatal experiences. Findings have implications for future models of treatment. |
| Arditti 2008 [13] | Excluded due to lack of CM background and age of children outside of perinatal range (mean age 13 years). | Maternal distress of 10 mothers following incarceration. Distress due to psychological, relational, situational, financial and environmental (prison) issues with recommendations for treatment and support. |
| Babcock 2014 [14] | Excluded due to age (not in perinatal period), and type of study design. | Single case study of a female about a range of life stressors and experiences, including sexual abuse and parenting. |
| Bacchus 2003 [15] | Excluded due to lack of CM background (DV and not past CM). | Exploration of help-seeking behaviours and health of 16 women (some in perinatal period) who have suffered from domestic violence. |
| Bacchus 2016 [16] | Excluded due to lack of CM background. | Qualitative interpretive study of the experiences of Interpersonal violence from 26 women whilst receiving early intervention during perinatal home visits in urban and rural areas in USA. |
| Backett-Milburn 2012 [17] | Excluded due to age of children (11-15 years). | Focus on children's experiences (via Child-Line) of their parent's health and wellbeing, its affect on them and how they make sense of their parenting experiences. |
| Baker 1993 [18] | Excluded due to age of children (not perinatal period). | The study looks at the reasons and implications for 6 parents involved in school and community politics over a decade in Massachusetts. |
| Baker 2001 [19] | Excluded due to children’s age (not perinatal period). | Qualitative study of 9 women with child sexual abuse backgrounds and examines cognitive self-understanding of its impact on parenting and promotes best practice for clinicians. |
| Ball 2010 [20] | Excluded due to lack of CM background. | Exploration of 80 Indigenous fathers in Canada about fatherhood and manhood in the context of changing gender relationships and regeneration cycles of care. |
| Banwell 2006 [21] | Excluded due to lack of CM background. | A comparative study of 70 childbearing women using illicit drugs with 3 other groups of women (with low incomes, mobile and medium/high incomes) found that these women were blames for their difficulties and not well supported. |
| Barclay 1996 [22] | Excluded due to lack of CM background. | Report on 53 men attending antenatal classes with their first-time pregnant partners. They discussed experiences in focus groups run by male midwives. |
| Barnes 2018 [23] | Excluded due to lack of CM background. | Study of impact of historical trauma on 7 parenting native Americans in an urban setting in New Mexico. |
| Barrett 2017 [24] | Excluded due to lack of CM background. | Explores paediatric nurses’ views of caring for infants who have suffered non-accidental injury |
| Bateman 2017 [25] | Excluded due to study type (meta synthesis and not qualitative). | A narrative synthesis of qualitative research was employed to systematically synthesize out of body narratives from existing studies. |
| Baxter 2014 [26] | Excluded due to topic focus, on ovarian cancer. | Thematic exploration of the lived experiences of 11 women diagnosed with ovarian cancer in their homes and workplaces. |
| Bayes 2008 [27] | Excluded due to lack of CM background (general population accessing care at hospital in Perth). | Postnatal qualitative aspect (thematic analysis) from a larger study involving 141 women pre and postnatal levels of childbirth fear in WA, Australia. |
| Bayes 2012 [28] | Excluded due to lack of CM background. | Qualitative study to contextualise the experiences of 28 Australian women who had elective caesareans for medical reasons. |
| Beck 2005 [29] | Excluded due to lack of CM background (sample of women that experienced a traumatic birth). | Experiences of an Internet type study with a sample of 40 women on birth trauma. |
| Beck 2006 [30] | Excluded due to lack of CM background (sample of women that experienced a traumatic birth). | A narrative analysis of 11 mothers' birth trauma stories. Implications focus on clinical provision of care. |
| Beck 2008 [31] | Excluded due to lack of CM background (sample of women that experienced a traumatic birth). | An exploration of the impact of the traumatic childbirth on mothers' (n=52) breastfeeding experiences. |
| Beck 2009 [32] | Excluded due to study type (single case study). | A single case study of a women's childhood sexual abuse and the impact on breastfeeding and the triggering effects of trauma. |
| Beck 2015 [33] | Excluded due to lack of CM background and topic (sample was nurse midwives and topic focussed on secondary traumatic stress from experiences of traumatic births). | Focussed on impact of traumatic birth on midwives. |
| Belknap 2007 [34] | Excluded due to topic not relating to perinatal or parenting, rather on life course of Mexican adolescents (not in perinatal period). | Descriptions of 24 Mexican women's experiences of violence and abuse in their family of origin. |
| Bell 2008 [35] | Excluded due to age of sample (not perinatal) and topic (not parenting focussed); and lack of CM background. | Examination of relationship between African American mothers under correctional supervision (with drug and alcohol addictions) and their maternal grandmothers. |
| Ben-Amitay 2015 [36] | Excluded due to age of sample (not perinatal) and topic (not parenting focussed). | A focus on metaphors of the victimisation experiences of 9 women which impacted on the 'self' and interpersonal relationships. |
| Benjamin 1996 [37] | Excluded due to study type. | Cross sectional study examining the extent symptoms of dissociative mothers interfere with their parenting and their subjective experiences of mothering |
| Benjamin 1998 [38] | Excluded due to lack of CM background (CM background of parents not clear). | Qualitative study to provide a clearer picture of 54 mothers with dissociative disorders and the impact on parenting. |
| Benoit 2010 [39] | Excluded due to not being a primary study. | Pregnant adolescents' unresolved trauma/mourning (using adapted Adult Attachment Interview) and links to self-report of socio-emotional adjustment. |
| Bertini 1996 [40] | Excluded due to study type. | Study of 97 single, married and divorced midlife women who were neglected in childhood and the impact on their marital status, parenting and self-esteem. |
| Bick 2017 [41] | Excluded due to age of children (unclear about age of children) and lack of CM background. | Explores health care needs, service use and challenges among women who became pregnant while in the trafficking situation in the United Kingdom (UK) and clinicians’ perspectives of maternity care for trafficked persons. |
| Blakey 2013 [42] | Excluded due to topic; Excluded due to children's age (11-15 years); unclear the study was about the prevalence of trauma with Child protection sample of mothers- the ages of children were not determined | Experiences of 26 African American mothers with substance-abuse histories who were trying to regain custody of their children, and 26 professionals who were helping these women accomplish that goal. |
| Boustani 2015 [43] | Excluded due to age (Unsure if the teens were in perinatal period- youth workers interviewed about inflated rates of teenage pregnancy and prevention) and lack of CM background. | Examines youth care workers’ perceptions of the specific and unique sexual health needs of youth at risk for foster care. |
| Brazelton 2011 [44] | Excluded due to age and excluded due to topic. | Exploration the meaning 17 African American women make of their experiences with childhood sexual abuse, and the many ways in which they have disclosed across the life course. |
| Breckenridge 2006 [45] | Excluded due to study design, as a review of themes in literature on mothering with histories of CSA.; not an original study with samples in perinatal period. | Study of how mothers with CSA are portrayed in the literature. |
| Brookes 2014 [46] | Excluded due to lack of CM background and type of study (Book chapter rather than original article). | An evaluation of 17 women in prison about 'Baby Steps' (a perinatal education program) |
| Brown 1998 [47] | Excluded due to sample (not perinatal) and topic (not parenting focussed on preschool children). | About the transmission of trauma through caretaking and patterns of intergenerational behaviours in holocaust families. |
| Brown 2011 [48] | Excluded due to lack of CM backgrounds (maltreatment or trauma backgrounds of pregnant teenagers). | A qualitative study that explored 9 pregnant teenagers and young mothers’ experience of power, control and domestic violence in their familial and intimate relationships. |
| Buchanan 2013 [49] | Excluded due to lack of CM background. | An exploration of the ways in which fear impacted on 16 women and their babies. |
| Buchbinder 2004 [50] | Excluded due to age of children (not perinatal) and lack of CM background not identified as an inclusion criteria. | The study describes and analyses 20 battered women’s subjective perceptions of their own motherhood. |
| Bundy-Fazioli 2013 [51] | Excluded due to age of children (youngest 3 years and not in perinatal period) and lack of CM background. | This research explores the experiences and perceptions of 25 mothers receiving services for child neglect. |
| Burnett 2016 [52] | Excluded due to lack of CM backgrounds, and topic focus (IPV, rather than perinatal / parenting). | A qualitative study of 10 rural women about their lived experience of IPV during pregnancy and the first 2 postpartum years. |
| Burnette 2016 [53] | Excluded due to age of children not known (not in perinatal period), and topic not focussed on perinatal / parenting experiences but parenting and healing from IPV | Study about historical oppression and uncovering risk factors for Indigenous families touched by violence in USA |
| Burnette 2017 [54] | Excluded due to age (not perinatal period); lack of CM background and topic focus. | Explores the evolution of victimisation within the lives of Indigenous women and uncover various risk factors for IPV victimisation. |
| Butler 1990 [55] | Excluded due to study design (quantitative). | An examination of the relationship between childhood sexual victimization and adolescent pregnancy of 41 young rural mothers who had been pregnant as teenagers. |
| Cahalane 2013 [56] | Excluded due to topic focus, age (not perinatal) and to lack of CM background. | The treatment implications arising from letters written by non-offending partners of men who have perpetrated child sexual abuse. |
| Caliso 1986 [57] | Excluded due to study design. | An investigation of the differences in child abuse potential amongst 96 female parents. |
| Callaghan 2015 [58] | Excluded due to age and lack of CM background. | Qualitative interviews with six Nigerian married women between the ages of 8 and 15 and their experiences of the transition to marriage, being married, pregnancy and their understanding of the marital and parental role. |
| Calvete 2014 [59] | Excluded due to topic focus, to age (not perinatal) and to study design. | An exploration of the characteristics of child-to parent violence (CPV) in Spain based on the narrations of adolescents who perpetrate this kind of violence, their parents, and the professionals who work in this area. |
| Carpiano 2002 [60] | Excluded due to lack of CM background and to age (not perinatal). | An investigation of the relationships among female domestic abuse survivors’ recovery from victimization, the influential effects of motherhood on the recovery process, and the real and perceived physical and psychological health of the survivor. |
| Cavanaugh 2015 [61] | Excluded due to age (not in perinatal period). | Experiences of 44 mothers who are child sexual abuse survivors. |
| Cecchet 2014 [62] | Excluded due to age (not in perinatal period) and to topic focus. | This qualitative research of 6 survivors of child and adolescent sex trafficking to assess factors that influenced their ability to survive, leave the sex trade, and reintegrate back into the community. |
| Cerulli 2012 [63] | Excluded due to age (not in perinatal period), to topic focus and to lack of CM background. | Focus groups with survivors to explore the need to address IPV consequences with an integrated model and begin to understand the interconnectedness between violence, health, and safety. |
| Chadwick 2014 [64] | Excluded due to lack of CM background. | Exploration of the factors associated with negative birth experiences in South African public maternity settings from the perspective of 33 women |
| Chambers 2010 [65] | Excluded due to age (not perinatal). | Exploration of the contributing factors to positive birthing experiences for sexual abuse survivors |
| Chanmugam 2011 [66] | Excluded due to children's age (about adolescents 12-14 and their mothers residing in Shelter). | As part of a larger qualitative study using Life Story methods, an ethnically diverse, 27 young adolescents (ages 1214) and their mothers, residing in four US domestic violence emergency shelters, were interviewed about their perspectives of shelter life. |
| Charbonneau-Dahlen 2010 [67] | Excluded due to topic focus and to age. | Documentation of contributing and residual effects of historical trauma among American Indian mission boarding school survivors and culturally responsive ways to enhance story telling (i.e. dream catcher-medicine wheel model). |
| Chien 2005 [68] | Excluded due to topic focus, to age and to study design. | Using a life history approach to explore how Taiwanese women with a history of CSA look back on the disclosure of CSA and their mothers' reactions. |
| Chilton 2015 [69] | Excluded due to topic focus. | Explores caregivers’ perceptions of the impact of their childhood adversity on educational attainment, employment and mental health. |
| Choi 2015a [70] | Excluded due to age and to lack of CM background. | Evaluation of a trauma informed perinatal care program with 47 participants (attendees in mental health, health care, and administrative staff roles) who found that the program was useful, but training needed to be more advanced. |
| Choi 2015b [71] | Excluded due to lack of CM background and to age. | Exploration of the perceived impact of parental drinking on children in a South African township where alcohol abuse is prevalent and high levels of existing poverty and violence may exacerbate potential consequences on children. |
| Clark 2011 [72] | Excluded due to study design. | A focus on the effects of child sexual abuse on the survivor, particularly on the effects for a childbearing woman. |
| Clarke 2010 [73] | Excluded due to lack of CM background. | Aimed to explore, understand, and describe nine Saskatchewan Aboriginal women's perceptions and beliefs regarding the nature and perceived cause of their depression post-birth, and to examine the role of socio-cultural factors related to the onset and maintenance of perinatal depression. |
| Cleveland 2016 [74] | Excluded due to lack of CM background. | Describes the mothering experiences of women with substance use disorders. |
| Coates 2014 [75] | Excluded due to lack of CM background. | A qualitative study of 17 women who experienced psychological problems in the first year after having a baby. |
| Coates 2017 [76] | Excluded due to lack of CM background. | Investigates experiences of women who have accessed a perinatal infant mental health (PIMH) service. |
| Cogan 1998 [77] | Exclusion due to age and lack of CM background. | In Vermont 25 women with serious mental illness were interviewed about relationship-based needs and support. |
| Cohen 1995 [78] | Excluded due to study design and age (not perinatal). | A preliminary report of an investigation of the maternal functioning of woman survivors of child sexual abuse. |
| Cole 1992 [79] | Excluded due to study design. | This study examined the self-reported parenting experience and practices of women who were incest victims as children. |
| Coleman 1997 [80] | Excluded due to age (not in perinatal period) and study design. | This paper reports on interviews with primary caretakers of children who have been abused and those with behaviour problems between six months and three and a half years after family preservation services ended. |
| Collins 1996 [81] | Excluded due to topic and lack of CM background. | Explores parents' perceptions of the risk of child sexual abuse and their protective behaviour. |
| Conn 2018 [82] | Excluded due to age (not in perinatal period) | Parent perspectives of screening for ACEs in the pediatric primary care setting, to understand their views on the potential impact of their ACEs on their parenting. |
| Coyer 2003 [83] | Excluded due to lack of CM background. | Discussion about parenting by women in recovery whilst addicted to cocaine. |
| Crawford 2014 [84] | Excluded due to topic focus and to age (not in perinatal period). | The study (from thesis) examined the relationship between the experience of trauma during childhood (ages birth-12) and life satisfaction in adulthood (ages 30-45) in a sample of eight (8) adults. |
| Cross 2001 [85] | Excluded due to age (not in perinatal period). | Reviews the available reports of parenting problems among mothers with CSA histories, and presents one clinical case and material from 4 out-patients who participated in a focus group to illustrate the complexities of treating mothers with CSA histories and their children. |
| Cross 2010 [86] | Excluded due to lack of CM background and topic focus. | A qualitative study to determine the rationale for 31 American Indian grandparents' who provide sole care of their grandchildren, the impact of historical trauma on their decision-making process in accessing services, the value of American Indian Child Welfare policies in addressing care issues, and custody status of the grand families. |
| Crumbley 1990 [87] | Excluded due to age (not in perinatal period). | Explored the experiences of non-abusive parents who were physically abused by their parents in relation to breaking intergenerational transmission of physical abuse. |
| Cummings 2018 [88] | Excluded due to lack of CM background, age (not perinatal) and topic focus. | The project developed a comprehensive, explanatory theory of the dynamic process by which parenting changes in response to a range of child trauma, using a sample of parents whose children had experienced a range of interpersonal trauma types. |
| da Silva 2018 [89] | Excluded due to age (not in perinatal period) and topic focus. | The study associates the life experiences of older women from the Northeast of Brazil with their suicidal ideation and attempts |
| Dahlen 2010a [90] | Excluded due to lack of CM background. | Exploration of ﬁrst-time mothers’ experiences of birth at home and in hospital in Australia. |
| Dahlen 2010b [91] | Excluded due to lack of CM background. | Exploration of ﬁrst-time mothers’ experiences of birth at home and in hospital in Australia same as above study). |
| Dalla 2003 [92] | Excluded due to lack of CM background and to age (not in perinatal period). | Examining intergenerational parent-child relational patterns among street-level sex workers and their parents and children. |
| Damant 2010 [93] | Excluded due to lack of CM background. | The paper draws upon the ﬁndings of a study that looked at women’s experiences of mothering in the context of co-occurrence of domestic violence and child abuse and considers the issue of women’s violence towards children – while acknowledging the fact that men are the main perpetrators of violence towards women and children in these families. |
| Danskin 2017 [94] | Excluded due to study design (quantitative) and age (not in perinatal period). | Investigates the impact of adverse childhood experiences and current parenting stress on beliefs regarding an 'ideal mother' among African American and Hispanic mothers. |
| Danto 2017 [95] | Excluded due to topic focus and age (not in perinatal period). | The study is a qualitative study of the mental health perceptions and practices of one Aboriginal community in the northern Ontario James and Hudson Bay region. |
| Davies 2000 [96] | Excluded due to age (not in perinatal period) and lack of CM background. | Children's and Primary Caretakers' Perceptions of the Sexual Abuse Investigation Process in New Zealand. |
| de Oliveira 2017 [97] | Excluded because published in Portuguese. | The study was to characterize obstetric violence experienced by women during the parturition process. |
| Denham 2008 [98] | Excluded due to topic focus, age (not in perinatal) and study design. | The article highlights how a four generation American Indian family contextualizes historical trauma and, speciﬁcally, how they frame their traumatic past into an ethic that functions in the transmission of resilience strategies, family identity, and as a framework for narrative employment. |
| Denov 2004 [99] | Excluded due to age (not in perinatal period) and topic focus. | The qualitative study explores the experience and long-term impact of sexual abuse by women. The data were derived from in-depth interviews with 14 adult victims (7men,7women) of child sexual abuse. |
| Devieux 2016 [100] | Excluded due to study design and lack of CM background. | The study derives from data from 224 pregnant and postpartum women in substance abuse treatment were analysed to examine effects of history of substance use, child abuse, and mental health problems on current substance use and condom-use. |
| DeVoe 2002 [101] | Excluded due to lack of CM background. | The article explores urban battered mothers’ perceptions of their preschool children’s exposure to domestic violence. It also examines mothers’ reports about their young children’s functioning and traumatic stress symptoms. |
| DeVoe 2003 [102] | Excluded due to lack of CM background. | Exploration of 43 women’s experiences of and barriers to help-seeking among battered mothers of children under six years of age |
| Dijkstra 1995 [103] | Excluded due to study design (case study from clinician perspective). | A case study of 2 mothers retrospective experiences on the long-term consequences of physical and sexual abuse, focused on how parents cope with their abusive childhood histories and manage to raise their children. |
| Dole 2009 [104] | Excluded due to lack of CM background. | A participatory research method explored the cultural meanings of mothering of nine African American adolescent mothers revealing how cultural mothering practices are being transferred generationally. |
| Domian 2010 [105] | Excluded due to topic focus (parents views’ of an intervention). | Identification of factors inﬂuencing mothers’ abilities to engage in a comprehensive parenting intervention program. |
| Dorr 2001 [106] | Excluded due to topic focus and age (not in perinatal period). | The article analyses the interviews of five men who came from abusive or otherwise unhealthy families to understand how they were able to overcome obstacles from their childhood to be more intimate with their own families. |
| Dossett 2017 [107] | Excluded due to study design and lack of CM background. | A single case study of a female pregnant patient with a range of mental health challenges. |
| Dufour 2001 [108] | Excluded due to age and study design (quantitative). | Exploration of variables that distinguish resilience in people experiencing addiction who were sexually abused as children. |
| Dumbrill 2006 [109] | Excluded due to lack of CM background and age (not in perinatal period). | Qualitative exploration of the ways in which parents experience and negotiate child protection intervention. |
| Dunlap 2003 [110] | Excluded due to topic focus and age (not in perinatal period). | Study of inner-city girls, early and then continued experiences of being compelled to have sex and the pathway leading to independent sexuality that often involved prostitution, teen pregnancy, early motherhood, school dropout, limited involvement with jobs, drug abuse, multiple children by different fathers and single-parent families. |
| Dunn 1993 [111] | Excluded due to topic focus and lack of CM background. | Study about nine adults who had been reared by mothers diagnosed with psychosis reported on their childhood experiences. |
| Efevbera 2017 [112] | Excluded due to topic focus and age (not in perinatal period) and lack of CM background. | Investigates what effective integration of ECD plus violence prevention can look like in LMICs with key informants. |
| Egeland 1988 [113] | Excluded due to study design. | Descriptive study to identify variables that distinguish mothers who broke the cycle of abuse from mothers who were abused as children and who also abused their own children. |
| Elizabeth 2006 [114] | Excluded due to study design and lack of CM background. | Post-traumatic Stress Disorder and the Violent Loss of a Child |
| El-Khani 2016 [115] | Excluded due to age (not in perinatal period) and lack of CM background. | The challenges of parenting in Syria refugee situation of immediate displacement |
| El-Khani 2017 [116] | Excluded due to age (not in perinatal period) and lack of CM background. | Coping mechanisms utilised by displaced Syrian refugee parents caring for their children in pre-settlement contexts |
| Ellsberg 2000 [117] | Excluded due to lack of CM background and age (not in perinatal period). | The study describes the characteristics of domestic violence against 488 women in Nicaragua |
| Engnes 2012 [118] | Excluded due to lack of CM background. | The study gains a deeper understanding of women’s experiences of being exposed to IPV during pregnancy. |
| Enriquez 2015 [119] | Excluded due to lack of CM background. | Multigenerational punishment and shared experiences of undocumented immigration status within mixed-status families. |
| Escobar-Chew 2015 [120] | Excluded due to age (not in perinatal period). | The qualitative study focused on disadvantaged women in the child welfare system who have lost their parental rights and an understanding how experiences of complex trauma inﬂuences parenting, health, and well-being. |
| Escoto-Lloyd [121] 2005 | Excluded due to lack of CM background. | Examination of the meaning of pregnancy intentions among Latina adolescents. |
| Escriba-Aguir 2013 [122] | Excluded due to lack of CM background and study design (quantitative). | A longitudinal study to determine the effect of isolated psychological IPV and psychosocial factors (social support and alcohol or drug use by a partner/family member) on psychological well-being (depression or poor self-perceived health status) at 5 and 12 months post-partum. |
| Eshed 2008 [123] | Excluded due to age (not in perinatal period). | Explores among 3 men, what, in participants' subjective perspectives, helped them break the intergenerational transmission cycle of maltreatment and show resilience in the domain of parenting. Ages of children 3-10. |
| Etheridge 2017 [124] | Excluded due to lack of CM background. | A study into men’s experiences of being present at childbirth this study explored the experiences of fathers who found childbirth traumatic. The aim of the research was to investigate how men coped with these experiences; the impact on their lives; and their views on what may have helped to reduce distress. |
| Etherington 1995 [125] | Excluded due to age (not in perinatal period). | The paper outlines the conclusions drawn from a qualitative research study which involved in-depth interviews with 2.5 adult males who had been sexually abused during childhood. |
| Etherington 2007 [126] | Excluded due to age (not in perinatal period). | A narrative of parenting as a turning point in drug users’ lives. |
| Evans 2010 [127] | Excluded due to lack of CM background. | Investigation of the experience of parenting education for single, Latina mothers with substance abuse histories who have persisted in attendance to completion of a parenting program. |
| Evans 2008 [128] | Excluded due to age (not in perinatal period) and lack of CM background. | A qualitative study to understand the mealtime and bedtime routines and rituals of 10 families of typically developing children (less than 6 years). |
| Falletta 2018 [129] | Excluded due to lack of CM background. | Perceptions of child protective services among pregnant or recently pregnant, opioid-using women in substance abuse treatment. |
| Farahzad 2016 [130] | Excluded due to lack of CM background and age (not perinatal). | An assessment of differences in drug use and depressive symptoms among female street sex workers (FSSWs) who have experienced child custody loss and those who have not. |
| Fenwick 2013 [131] | Excluded due to lack of CM background. | A study of 33 women’s perceptions of perinatal emotional support interventions following childbirth |
| Few-Demo 2014 [132] | Excluded due to lack of CM background. | A qualitative study involving a follow-up interview with 10 incarcerated and re-entry mothers in rural southwest and central Virginia was conducted to explore the influence that women’s close relationships have on their re-entry experiences with their families. |
| Finnbogadóttir 2012 [133] | Excluded due to lack of CM background. | An exploration of midwives awareness of and clinical experiences of domestic violence among pregnant women in Southern Sweden. |
| Finnbogadottir 2014 [134] | Excluded due to lack of CM background. | A grounded theory model of women’s experiences of IPV during pregnancy and how they handled the situation. |
| Firestone 1989 [135] | Excluded due to age (not perinatal), lack of CM background and study design. | A study of parenting groups based on voice therapy. |
| Fisher 2000 [136] | Excluded due to age (not perinatal). | Explored the relationships between mental representations of attachment and caregiving among women sexually abused during childhood to better understand individual differences in the intergenerational transmission of trauma. |
| Fitzgerald 2005 [137] | Excluded due to study design and age (not perinatal). | Study of perceptions of parenting versus parent-child interactions among incest survivors. |
| Flam 2013 [138] | Excluded due to age (not perinatal) and topic focus. | A qualitative study on the circumstances facilitating adults awareness of children's first sign of sexual abuse. |
| Fonfield-Ayinla 2009 [139] | Excluded due to lack of CM background. | A commentary on a consumer perspective on parenting while homeless. |
| Forssen 2012 [140] | Excluded due age (not perinatal) and to lack of CM background. | Lifelong Significance of Disempowering Experiences in Prenatal and Maternity Care: Interviews With Elderly Swedish Women |
| Foster 2017 [141] | Excluded due to age (not perinatal) and topic focus. | A narrative analysis of the fears and futures of boy victims of Sexual Abuse |
| Fowler 2004 [142] | Excluded due to lack of CM background and study design. | Case study of one woman's experience and memory of learning to mother during pregnancy and the first postnatal year. |
| Fowler 2018 [143] | Excluded due to lack of CM background. | Explores the factors affecting the delivery and outcomes of parenting programs in correctional facilities in New South Wales Australia from the perspective of individuals involved in developing and implementing the programs. |
| Franz 2017 [144] | Excluded due to age (not perinatal) and lack of CM background. | An examination of the experiences, parenting practices, and utilization and perception of community supports among single Latina mothers experiencing poverty. (* from Franz 2016) |
| Frisman 2012 [145] | Excluded due to topic focus and lack of CM background. | The experience of becoming a grandmother (11 women) to a premature infant – a balancing act, inﬂuenced by ambivalent feeling |
| Frost-Pineda 2009 [146] | Excluded due to lack of CM background. | Explores addiction and fertility through a review of the literature and original qualitative and quantitative research in a population of women in residential treatment for Substance Use Disorders (SUDs). |
| Gaensbauer 2009 [147] | Excluded due to age (not perinatal) and topic focus. | A psychoanalytic perspective on early trauma from interviews with 30 analysts who treated an adult victim of a circumscribed trauma in early childhood. |
| Galera 2005 [148] | Excluded due to lack of CM background. | A study on maternal role-related perceptions, beliefs and attitudes in which women with small children and are undergoing or underwent treatment for alcohol or drug addiction live. |
| Ghaffar 2012 [149] | Excluded due to age (not perinatal). | Exploration of the experiences of parents and carers whose children have been subject to child protection plans. |
| Gil 2010 [150]. | Excluded due to age (not perinatal). | Exploration of the perceptions of mothering expressed by a sample of eight women who experienced childhood sexual abuse and to explore the protective factors that promoted their positive mothering experiences. (*from Gil 2009) |
| Gilgun 2013 [151] | Excluded due to lack of CM background and age (not in perinatal period). | Mothers’ perspectives on signs of child sexual abuse in their families. |
| Gojman-de-Millan 2017 [152] | Excluded due to lack of CM background | A dynamic understanding for clinicians of mental collapse as “disorganized attachment”. |
| Gold 2007 [153] | Excluded due to study design (quantitative), lack of CM background and age (not perinatal). | The study examined relations between posttraumatic stress disorder (PTSD) symptom severity and several family adjustment variables among a sample of 89 female Vietnam veterans and their male relationship partners. |
| Goldberg 1995 [154] | Excluded due to study design. | Reviews and interpretation of some recent literature on substance abuse problems and treatments among women.. |
| Goldberg 2010 [155] | Excluded due age (not perinatal), study design and to lack of CM background. | An interdisciplinary case study of a 4-year 11-month-old child born to a mother addicted to meth revealed significant cognitive and communicative delays |
| Goldblatt 2009[156] | Excluded due to age (not perinatal) and topic focus. | A report of a study of the impact of caring for abused women on nurses’ professional and personal life experiences. |
| Golden 2013 [157] | Excluded due to study design, lack of CM background and age (not perinatal). | A study on how economic resources, gender beliefs, and neighbourhood disadvantage influence IPV. |
| Golding 2015 [158] | Excluded due to study design. | Study on supporting parents to meet the challenges of parenting children who have been traumatised within their early parenting environments. |
| Goldsmith 2011 [159] | Excluded due to study design. | Discussion about the devastating experience of the death of an infant or child affects the maternal attachment to surviving children. |
| Goldson 1976 [160] | Excluded due to study design. | A report on non-accidental trauma and failure to thrive. |
| Golfenshtein 2017 [161] | Excluded due to lack of CM background. | Investigation of the coping mechanisms of mothers who’s infant with complex congenital heart disease is admitted in the Cardiac Intensive Care Unit and the exploration of mindfulness as a potential stress-reduction intervention for these mothers. |
| Golub 2016 [162] | Excluded due to study design and lack of CM background. | The effects of in utero environment and maternal behaviour on neuroendocrine and behavioural alterations in a mouse model of prenatal trauma. |
| Gomez 2012 [163] | Excluded due to study design. | Working with parents with children with complex trauma via a Comprehensive EMDR Treatment. |
| Gondwe 2015 [164] | Excluded due to study design (quantitative systematic review) and lack of CM background. | A review of evidence on the incidence, prevalence, and impact of PTS symptoms following preterm birth, predictors of PTS symptoms, screening and management, and to identify the gaps and the applicability of the evidence to developing countries such as Malawi. |
| Gondwe 2017 [165] | Excluded due to study design (quantitative) and lack of CM background. | A longitudinal study of the role of sociodemographic factors in maternal psychological distress and mother-preterm infant interactions. |
| Gonzalez 1994 [166] | Excluded due to study design and lack of CM background. | Preview about prenatal exposure to cocaine and the effect on development |
| Gonzalez 2012 [167] | Excluded due to study design. | The mediating role of cortisol and executive functioning on maternal early life experiences and parenting. |
| Goodkind 2012 [168] | Excluded due to age (not perinatal). | Promotion of the mental health and wellbeing of Diné (Navajo) youth, and exploration of the relevance of addressing historical trauma and current structural stressors to build on individual and community strengths through healing and social transformation at multiple levels. |
| Goodman 1991 [169] | Excluded due to study design. | A comparative study of the prevalence of 50 homeless and 50 house poor mothers about physical and sexual abuse. |
| Goodman 2015 [170] | Excluded due to study design and lack of CM background | A case report on the complexities in the treatment of a pregnant woman with opioid use disorder and posttraumatic stress disorder and reviews the psychotherapeutic and pharmacologic approaches available to treat these co-occurring disorders in pregnancy. |
| Goodman 2017 [171] | Excluded due to topic focus, study design and age | An assessment of household and maternal factors associated with street-migration of children through self-report of 1974 randomly selected women in semi-rural Kenya. |
| Goodyear 2002 [172] | Excluded due to study design and lack of CM background. | A study of 493 pregnant Latina teenagers about psychosocial and developmental determinants of how they select and perceive the men who father their children. |
| Goosen 2014 [173] | Excluded due to study design and age (not perinatal). | A longitudinal medical record study about frequent relocations between asylum-seeker centres are associated with mental distress in asylum-seeking children. |
| Gordon 1989 [174] | Excluded due to study design, age (not perinatal) and lack of CM background. | An investigation of the behaviour of mothers with depression, bipolar disorder and chronic medical illness with normal subjects, toward their children during an observed conflict discussion task. |
| Gosh Ippen 2014 [175] | Excluded due to study design. | Report on clinical considerations for conducting child-parent psychotherapy with young children with developmental disabilities who have experienced trauma. |
| Goutaudier 2011 [176] | Excluded due to lack of CM background. | Exploration of the experience of premature infants’ mothers, the way they are taken care of, how they cope with this traumatic experience as well as the psychopathological and psychosocial consequences post-delivery. |
| Goutaudier 2012 [177] | Excluded due to study design and to lack of CM background. | Assessment of the contribution of negative emotions, childbirth pain, perinatal dissociation, and feelings of self-efficacy to the development of posttraumatic stress disorder (PTSD) symptoms following childbirth. |
| Grabow 2017 [178]. | Excluded due to study design and age (not perinatal). | This study of 541 adoptive and126 biological families, examined the heritable and environmental mechanisms by which maternal trauma and associated depressive symptoms are linked to child internalizing and externalizing behaviours. |
| Grafwallner 2016 [179] | Excluded due to age (not perinatal) and study design. | Discusses lessons learned from the school-to-work journey of a five-year-old adopted daughter from an orphanage in Bulgaria. |
| Graham-Bermann 1997 [180] | Excluded due to study design, age (not perinatal) and to lack of CM background | A study about the social functioning of preschool-age children whose mothers were emotionally and physically abused. |
| Graham-Bermann 2005 [181] | Excluded due to study design, age (not perinatal) and to lack of CM background. | A study on the violent exposure and traumatic stress symptoms as additional predictors of health problems in high risk children. |
| Graham-Bermann 2006 [182] | Excluded due to study design, age (not perinatal) and to lack of CM background. | An assessment of traumatic stress symptoms for 218 children ages 5 to 13 following exposure to IPV and diagnosis of posttraumatic stress disorder. |
| Graham-Bermann 2008 [183] | Excluded due to study design, age (not perinatal) and to lack of CM background. | An assessment of traumatic events and stress symptoms in preschool children from low income families. |
| Graham-Bermann 2009 [184] | Excluded due to study design and age (not perinatal). | Evaluation of the social and emotional adjustment of 219 children in families with varying levels of IPV using a model of risk and protection. |
| Graham-Bermann 2010 [185] | Excluded due to study design, age (not perinatal) and to lack of CM background. | Exploration of 87 preschool-aged children and their mothers exposed to IPV within the last two years to ascertain verbal ability, history of violence, and exposure to trauma. |
| Graham-Bermann 2011a [186] | Excluded due to study design, age (not perinatal) and to lack of CM background. | Investigates mediators and moderators of change in adjustment following intervention for children exposed to intimate partner violence. |
| Graham-Bermann 2011b [187] | Excluded due to study design, age (not perinatal) and to lack of CM background. | Investigates whether disclosure is therapeutic for children following exposure to traumatic violence. |
| Graham-Bermann 2013 [188] | Excluded due to study design and age (not perinatal). | Assessment of a 10-week, group therapeutic-oriented community-based intervention, the Moms’ Empowerment Program (MEP), was tested with 181 children and their mothers exposed to IPV. |
| Granek 2014 [189] | Excluded due to lack of CM background and age (not in perinatal period). | An exploration of how single parents of children with cancer describe their caregiving experiences and to understand their contextual life stressors. |
| Granqvist 2014 [190] | Excluded due to study design, age (not perinatal) and lack of CM background. | A small matched comparison study of mothers with intellectual disability, their experiences of maltreatment, and their children’s attachment representations. |
| Grant 2014 [191] | Excluded due to study design and lack of CM background. | A study about improving pregnancy outcomes among high-risk mothers who abuse alcohol and drugs. |
| Grasso 2016 [192] | Excluded due to study design, age (not perinatal) and lack of CM background. | An examination of the overlap of the quality and frequency of psychological and physical forms of IPV and harsh parenting. |
| Greaves 2006 [193] | Excluded due to lack of CM background and age (not in perinatal period). | A study of changes in substance use among women in shelters for abused women and children. |
| Green 1995 [194] | Excluded due to study design. | The case histories of four women who developed symptoms of post-traumatic stress disorder following the disclosure of the sexual abuse of their daughters. |
| Greene 2018 [195] | Excluded due to lack of CM background, and age (not perinatal). | The role of maternal posttraumatic stress symptoms and parenting behaviours on psychological and physical intimate partner violence and young children’s mental health. |
| Greif 1993 [196] | Excluded due to lack of CM background, topic focus and age (not in perinatal). | A report on the common issues for parents in a methadone maintenance group. |
| Grekin 2017 [197] | Excluded due to study design and topic focus. | An examination of the trajectories of depression from pregnancy through 24 months postpartum in an at-risk population and the effects of trauma on perinatal depression. |
| Guardino 2014 [198] | Excluded due to primary study, study design and lack of CM background. | The outcome and recommendations from a systematic review about coping during pregnancy. |
| Haggett 2013 [199] | Excluded due to age (not perinatal) and study design. | Lived experience of one mother struggling to overcome the adversity of her childhood to create a nurturing environment for her children. |
| Haight 2007 [200] | Excluded due to lack of CM background. | The perspectives and strategies from women involved in child protective services. |
| Haight 2009 [201] | Excluded due to uncertainty of parental CM background. | A case-based analysis of rural and Midwestern mothers' experiences of methamphetamine addiction. |
| Hall 2011 [202] | Excluded due to age (not perinatal). | A strength-based examination of intergenerational cycles of child abuse and explore strategies for non-abuse in parenting. |
| Hall 2000 [203] | Excluded due to topic focus and age (not perinatal). | A report on women survivors of childhood abuse and the impact of traumatic stress on education and work. |
| Halperin 2015 [204] | Excluded due to study design and lack of CM background. | A comparative study between Israeli Jewish and Arab women about the influence of childbirth experiences on women's postpartum traumatic stress symptoms. |
| Halsey 2012 [205] | Excluded due to age (not perinatal). | An exploration of the dynamics of repeat incarceration and release of a small cohort of young men from the perspectives of their formerly incarcerated fathers. |
| Halvorsen 2013 [206] | Excluded due to lack of CM background. | The study examines and illuminates how women previously subjected to rape experience giving birth for the first time and their advice on the kind of birth care they regard as good for women with a history of rape. |
| Hanlon 2010 [207] | Excluded due to lack of CM background and study design. | A qualitative exploration of the sociocultural context of antenatal mental distress in a rural Ethiopian community. |
| Harner 2013 [208] | Excluded due to topic focus, age (not perinatal) and to lack of CM background | Responses from women in a maximum-security prison and the effect on their mental health. |
| Harris-McKoy 2015 [209] | Excluded due to lack of CM background and age (not in perinatal period). | Exploration of the experiences and observations of marriage and family therapists (MFTs) conducting an empirically supported parenting program at a transitional homeless community. |
| Harvey 2012 [210] | Excluded due to lack of CM background and study design (quantitative). | A qualitative study with fathers present during the resuscitation of their baby at delivery. |
| Hayes 2007 [211] | Excluded due to topic focus and lack of CM background. | Explores experience of mothering after prison. |
| Henderson 1993 [212] | Excluded due to topic focus. | Phenomenological study looking at abused women's perceptions of their children's experiences while living with their mothers during and after leaving an abusive marriage. |
| Henriksen 2017 [213] | Excluded due to topic focus and lack of CM background. | Explores factors associated with a negative childbirth experience including descriptions from women themselves. |
| Herland 2017 [214] | Excluded due to age. | As part of a 30-year-long follow-up study, the paper examines how participants with troubled upbringings experience social norms of motherhood. |
| Herland 2015 [215] | Excluded due to age (10-20yrs), uncertain about CM background and not primary study. | Experiences of fatherhood among fathers with a difficult upbringing and past. |
| Heward-Belle 2017 [216] | Excluded due to age (children’s age not mentioned) and topic (perpetrating violence on mothers and children). | Examination of the ways that 17 domestically violent Australian men assault women as mothers and their mothering. |
| Hill 2013 [217] | Excluded due to lack of CM background. | Examines Native grandparent caregivers' motivations and circumstances that resulted in them assuming the full-time caring role. |
| Hilton 2014 [218] | Excluded due to topic focus, lack of CM background and age (not perinatal). | This research identifies survival patterns and stress related to 53 homeless parents’ struggles in Michigan’s Upper Peninsula. |
| Hinton 2014 [219] | Excluded due to lack of CM background and study design. | A quality study of partner experiences of ‘‘Near-Miss’’ events in pregnancy and childbirth in the UK. |
| Hinton 2015 [220] | Excluded due to lack of CM background and study design. | Support for mothers and their families after life-threatening illness in pregnancy and childbirth: |
| Hodgdon 2018 [221] | Excluded due to lack of CM background. | Dissertation examines the experiences of young mothers enrolled in an innovative, gender-responsive, and trauma-informed treatment approach via qualitative and quantitative methods. |
| Hogg 2015 [222] | Excluded due to lack of CM background and study design (book chapter for impact and evidence series). | Documented evidence from a relationships based perinatal education programme "Baby Steps" that is delivered in the UK. |
| Holka-Pokorska 2016 [223] | Excluded due to study design and lack of CM background. | Discussion about the different mental disorders, which may determine psychiatric indications for elective caesarean services. |
| Holliday 2018 [224] | Excluded due to lack of CM background and topic focus. | A qualitative exploration of racial differences in pregnancy intention, reproductive coercion, and partner violence among family planning clients. |
| Holt 2011 [225] | Excluded due to age (not perinatal) and lack of CM background. | A three-year Irish research study that focuses on the decision-making process in child contact, specifically the assessment and management of risk of continuing abuse to young people previously exposed to domestic abuse. |
| Holt 2015 [226] | Excluded due to age (not perinatal) and lack of CM background. | Challenges and contradictions for post-separation fathering and domestic abuse. |
| Holt 2017 [227] | Excluded due to age (not perinatal) and lack of CM background. | A mixed-methods study to consider the paradoxical post-separation position many women find themselves occupying when child contact necessitates the continued and mainly unmonitored presence of abusive men in their lives and the lives of their children. |
| Hooper 2004 [228] | Excluded due to age (not perinatal) and study design. | Exploration of the vulnerabilities of children whose parents have been sexually abused in childhood. |
| Hudson 2016 [229] | Excluded due to study design and age (not perinatal). | A look at how one overcomes the worst of disabilities associated with complex childhood trauma, the family support that the community needs to distinguish from parents behaviours. |
| Hughes 2010 [230] | Excluded due to lack of CM background and age (not in perinatal period). | A qualitative exploration of the utility of motivational interviewing in domestic violence shelters. |
| Hughes 2011 [231] | Excluded due to age (not perinatal) and lack of CM background. | An analysis of what mothers, who have experienced intimate partner violence, say about their involvement in the child protection system. |
| Hurd 2002 [232] | Excluded due to age (not perinatal) and lack of CM background. | A study of sibling support systems in childhood after parent dies. |
| Iles 2015 [233] | Excluded due to topic focus and lack of CM background. | The development of a grounded theory model of first-time mothers’ experiences of postnatal posttraumatic stress, with the aim of aiding understanding, formulation and treatment of new mothers. |
| Insetta 2015 [234] | Excluded due to lack of CM background and age (not perinatal). | Mothers experiencing IPV and their messages and strategies for communicating with children to break the cycle of violence. |
| Izaguirre 2015 [235] | Excluded due to topic focus and lack of CM background. | Interviewing mothers to understand its impact on their children who are exposed to IPV. |
| Jack 2005 [236] | Excluded due to topic focus, lack of CM background and age (not perinatal). | A qualitative study of mothers with post-traumatic stress disorder after traumatic childbirth who struggled to survive and experienced nightmares, flashbacks, anger, anxiety, depression, and isolation. |
| Jack2017 [237] | Excluded due to lack of CM background. | Development of strategies for the identification and assessment of intimate partner violence in a nurse home visitation programme. |
| Jackson 2017 [238] | Excluded due to topic focus and lack of CM background. | Explores the experiences of twelve parents who were, at the time of the study, subject to statutory child protection intervention measures in Scotland. |
| Jacobs 2014 [239] | Excluded due to lack of CM background and age (not in perinatal period). | The life stories of South African mothers’ who had a heavy drinking problem and their barriers to accessing treatment. |
| Jammeh 2011 [240] | Excluded due to topic focus and lack of CM background. | A qualitative assessment of the barriers to emergency obstetric care services in perinatal deaths in rural Gambia. |
| Jewkes 2005 [241] | Excluded due to age (not perinatal) and lack of CM background. | Reflections from 77 interviews on gender in the social context of child rape in South Africa and Namibia. |
| Johnson 2002 [242] | Excluded due to topic focus and age (not perinatal). | A qualitative study that describes the childhood experiences of five imprisoned African American women who have extensive histories of drug addiction and criminal behaviour. |
| Johnston 2014 [243] | Excluded due to lack of CM background and study type. | This case study details the experience of a young black man from a high-risk background during the pregnancy, birth, and hospitalization of his premature son. |
| Jones 2017a [244] | Excluded due to age (not perinatal) and lack of CM background. | A narrative analysis of 8 mothers’ accounts of how they coped, both during an abusive relationship and after leaving. |
| Jones 2017b [245] | Excluded due to age (not perinatal) and topic focus. | Life course impact of emotional abuse. |
| Kadish 2015 [246] | Excluded due to age (not perinatal), lack of CM background and topic focus. | A qualitative analysis of five women’s recollections and reflections on being raised by a mother with psychosis. |
| Kallan 2014 [247] | Excluded due to lack of CM background and topic focus. | Mixed qualitative methods study examining parenting in the medicalised context of neonatal intensive care unit. |
| Kamal 2017 [248] | Excluded due to lack of CM background and top (IPV and parenting) | A study of the perceptions and experiences of an attachment-based Intervention for parents troubled by intimate partner violence. |
| Kamite 2017 [249] | Excluded due to topic focus. | Experience of subsequent generation Hiroshima bomb survivors. |
| Kanku 2010 [250] | Excluded due to lack of CM background and topic focus. | Teenage attitudes regarding contraception. |
| Kantrowitz-Gordon 2016 [251] | Excluded due to lack of CM background and topic focus. | Experience of preterm birth. |
| Karver 2016 [252] | Excluded due to lack of CM background and topic focus. | Experience of gender roles and sexuality. |
| Kassam-Adams 2015 [253] | Excluded due to study design and topic focus. | Survey of paediatric nurses and primarily related to recognising paediatric trauma related to acute life-threatening events. |
| Katsumaru 2012 [254] | Excluded due to study design, primary study and topic focus. | Conference abstract only focussing on experiences of anorexia nervosa. |
| Katz 2003 [255] | Excluded due to topic focus and lack of CM background. | Case study of three nurses and their careers. |
| Katz 2017 [256] | Excluded due to topic focus, age (not perinatal) and lack of CM background. | Experiences of adult military sexual assault. |
| Katz 2014 [257] | Excluded due to children's age (not perinatal), unclear childhood maltreatment history of parent, and not a primary source. | How mother and child relationships are supported and strengthened as part of domestic violence recovery. |
| Katz 2015 [258] | Excluded due to age (not perinatal), lack of CM background and topic focus. | Recovery from adult domestic violence with parents with children over 10 years of age. |
| Katz 2016 [259] | Excluded due to age (not perinatal) and lack of CM background. | Recovery from adult domestic violence with parents with children over 10 years of age. |
| Kaye 2007 [260] | Excluded due to lack of CM background and topic focus. | Experiences of survivors of adult domestic violence. |
| Kaye 2014a [261] | Excluded due to lack of CM background and topic focus. | Experiences of obstetric trauma. |
| Kaye 2014b [262] | Excluded due to lack of CM background and topic focus. | Experiences of uterine rupture. |
| Kearney 2012 [263] | Excluded due to age (not perinatal) and lack of CM background. | Pilot intervention study for school-aged children exposed to domestic violence. |
| Keim 2017 [264] | Excluded due to lack of CM background and topic focus. | Parent experiences of infant dying in neonatal intensive care. |
| Keiski 2016 [265] | Excluded due to topic focus and age (not perinatal). | Women’s experiences of perpetrating family violence. |
| Kelleher 2012 [266] | Excluded due to topic focus. | Experiences of parents who have been notified to child protection. |
| Kelly Cardona 2016 [267] | Excluded due to lack of CM background and topic focus. | Focus on parents with substance use disorders. |
| Kenny 2015 [268] | Excluded due to lack of CM background and topic focus. | Experiences of young parents experiencing homelessness. |
| Kidner 2018 [269] | Excluded due to lack of CM background and age. | Focus on recent domestic violence |
| Kidner 2004 [270] | Excluded due to age (not perinatal) and lack of CM background. | Describes the experience of mothers whose pregnancies were complicated with HELLP syndrome. |
| Killion 1998 [271] | Excluded due to lack of CM background. | Focus on experiences of homelessness |
| Kilroy 2014 [272] | Excluded due to age (not perinatal) and lack of CM background. | Focus on children who have experienced sexual abuse, and their parent’s ability to support them. |
| Kirkman 2017 [273] | Excluded due to topic focus and lack of CM background. | Abortion experiences of women breast cancer survivors. |
| Kitzinger 2007 [274] | Excluded due to lack of CM background. | Experiences of birth trauma. |
| Kitzinger 2012 [275] | Excluded due to study design and lack of CM background. | Experiences of birth trauma. |
| Koren-Karie 2004 [276] | Excluded due to child’s age (not perinatal). | Examination of the co-construction of emotion dialogues between mothers and their 6-year-old children in light of mothers' experiences of being sexually, physically, and emotionally abused during childhood. |
| Koren-Karie 2008 [277] | Excluded due to age (not perinatal). | Psychometric study involving parents of children aged 4-10 years. |
| Kovalesky 2001 [278] | Excluded due to age (not perinatal) and lack of CM background. | Experiences of parents with substance abuse and child custody loss. |
| Kruger 2014 [279] | Excluded due to lack of CM background. | Experiences of parents experiencing depression and extreme anger. |
| Kruk 2010 [280] | Excluded due to lack of CM background and topic focus. | Experiences of divorced mothers with custody loss. |
| Kulkarni 2006 [281] | Excluded due to lack of CM background. | Participant’s adolescent mothers experiencing IPV. |
| Kyskan 2007 [282] | Excluded due to lack of CM background. | Explores the profound experience of becoming a mother in the context of an addiction. |
| Lainsbury 2009 [283] | Excluded due to study design and age (not in perinatal). | Father describes his personal experience of becoming a parent after CSA. |
| Laird-Bloom 2002 [284] | Excluded due to topic focus and age (not perinatal). | Describes the lived experience of male partners in relationship with female childhood sexual abuse survivors. |
| Lake 2002 [285] | Excluded due to age (not perinatal) and topic focus. | Explores the differences between men and women who witnessed domestic violence as children and their current adult relationships. |
| Lake 2004 [286] | Excluded due to age (not perinatal) and topic focus. | Participants are not specifically parents. In-depth analysis of recovery from child maltreatment in adulthood. |
| LaMancuso 2016 [287] | Excluded due to study design, primary study and lack of CM background. | Participants are refugees (not specifically child maltreatment trauma). |
| Lamb 2010 [288] | Excluded due to study design, primary study and lack of CM background. | No explicit mention of child maltreatment history among participants. |
| Lanctot 2017 [289] | Excluded due to age (not perinatal). | A narrative of the obstacles to the attainment of motherhood ideals among 13 adult women formerly placed in residential care. |
| Lapierre 2010 [290] | Excluded due to lack of CM background. | Explores women’s experiences of mothering in the context of current domestic violence. |
| Lear 2006 [291] | Excluded due to study design, primary study and lack of CM background. | Commentary on birth trauma. |
| Leeners 2007 [292] | Excluded due to study design and topic focus. | Case study of gynaecologic experiences after CSA. |
| Leeners 2016 [293] | Excluded due to study design. | Case study of birthing experiences after CSA. |
| Leon 2004 [294] | Excluded due to study design. | Observational study of role of unresolved loss on parenting transition. |
| Lewinsohn 2018 [295] | Excluded due to lack of CM background. | Experiences of parents with unplanned pregnancy. |
| Limoges 2015 [296] | Excluded due to age and lack of CM background. | Grandparents experiences of custodial care. |
| Lingen-Stallard 2016 [297] | Excluded due to lack of CM background and topic focus. | Experience of perinatal HIV testing. |
| Linton 2014 [298] | Excluded due to lack of CM background. | Pregnancy/birth experiences of adolescents with a disability. |
| LoGuidice 2016 [299] | Excluded due to age (not perinatal). | Examines the lived experience of pregnancy, labor, and birth from survivors. |
| LoGuidice 2018 [300] | Excluded due to study design lack of CM background. | Case study of birth experience of mothers with history of adult sexual assault |
| LoVerso 2017 [301] | Excluded due to age (not perinatal). | Explores experiences of motherhood and recovery among six female survivors of child sexual abuse (children aged 4-23). |
| Low 2003 [302] | Excluded due to lack of CM background. | Adolescent experiences of birth. |
| Lupton 2014 [303] | Excluded due to lack of CM background. | Case study and theoretical exploration of intergenerational transmission of depression. |
| Lutenbacher 2004 [304] | Excluded due to topic focus and age (not perinatal). | Participants are domestic violence victims, not specifically parents. |
| Lutz 2005 [305] | Excluded due to age (not perinatal) and lack of CM background. | Women's experiences and perceptions of intimate partner abuse during the childbearing cycle. |
| Lyndon 2018 [306] | Excluded due to lack of CM background. | Parents perceptions of safety during birth. Not specifically related to trauma. |
| Maker 2000 [307] | Excluded due to study design. | Case study of parenting after child maltreatment |
| Marina 2014 [308] | Excluded due to topic focus. | Focus on male experiences of domestic violence in childhood and the impact during adulthood (not specifically parenting). |
| Markert 2011 [309] | Excluded due to topic focus, age (not perinatal) and lack of CM background. | Case study of transgenerational effects of Chinese cultural revolution. |
| Martin 1992 [310] | Excluded due to study design and topic focus. | Longitudinal follow-up of outcomes in adulthood (not specifically parenting). |
| Martinez 2015 [311] | Excluded due to age (not perinatal) and lack of CM background. | Parent experiences of IPV. |
| Martsolf 2008 [312] | Excluded due to age (not perinatal) and topic focus. | Describes how childhood adversity influences the life course of survivors of childhood sexual abuse.. |
| Mason 2005 [313] | Excluded due to lack of CM background. | Experiences of psychiatric inpatients with postpartum depression. |
| Mathews 2011 [314] | Excluded due to age (not perinatal) and topic focus. | Exploration of previous experiences of men who killed their partners. |
| Mbonye 2012 [315] | Excluded due to topic focus, age (not perinatal) and lack of CM background. | Exploration of role of life history events on gender equity among sex workers. |
| McComish 1999 [316] | Excluded due to study design and topic focus. | Intervention study of treatment for grief among substance using women. |
| McConnell 2017 [317] | Excluded due to age (not perinatal), lack of CM background and topic focus. | Evaluation of intervention to support dads at risk of continuing perpetration of child abuse. |
| McGaw 2018 [318] | Excluded due to lack of CM background and age (not perinatal). | Parenting experiences after military PTSD. |
| McGhan 2005 [319] | Excluded due to lack of CM background. | Explored factors that contributed to re-infection with gonorrhea (GC) or chlamydia (CT) among low income, inner-city adolescent girls. |
| McGhee 2017 [320] | Excluded due to lack of CM background. | Case study of mother involved with child protection. |
| McMahon 2008 [321] | Excluded due to study design and age (not perinatal). | Observational study of fatherhood and substance use/methadone treatment. |
| McWey 2013 [322] | Excluded due to lack of CM background. | Exploration of perceptions of parental history of parenting among parents involved in CPS |
| Meadows-Oliver 2006 [323] | Excluded due to primary study and lack of CM background. | Experiences of homeless adolescent mothers. |
| Meaney 2016 [324] | Excluded due to lack of CM background. | Experiences of maternal morbidity. |
| Meek 2007 [325] | Excluded due to lack of CM background. | Explores the parenting aspirations and concerns of young fathers in prison. |
| Mehta 2017 [326] | Excluded due to lack of CM background. | Explores reasons for preferring unscheduled maternity care. |
| Menashe 2014 [327] | Excluded due to lack of CM background. | Experiences of parenting among child protection workers. |
| Mendel 2016 [328] | Excluded due to study design. | Observational study involving first mothers examining the facets of childhood well-being that prove most salient in such distal health outcomes which hampers prevention and intervention efforts. |
| Mendis 2009 [329] | Excluded due to topic focus (but unclear). | Discusses feminist methodology and highlights the concepts that proved invaluable in collecting rich data of a sensitive nature. |
| Meredith 2017 [330] | Excluded due to lack of CM background. | Subsequent pregnancy experiences of parents after previous perinatal loss. |
| Milch 2012 [331] | Excluded due to study design and lack of CM background. | Case study of attachment security. |
| Miller 2011 [332] | Excluded due to primary study and lack of CM background. | Commentary about PTSD in obstetric care providers. |
| Milligan 2002 [333] | Excluded due to lack of CM background. | Perspectives of prenatal care among vulnerable populations. |
| Mohler 2001 [334] | Excluded due to study design. | Case study of intergenerational transmission of childhood trauma and healing therapy |
| Monaghan- Blout 1999 [335] | Excluded due to topic focus and age (not perinatal). | A manuscript focusing on parents who were either at risk for or had actually abused their children, but chose to find an alternative to the parent-child relationship they learned from their own family. |
| Montgomery 2015c [336] | Excluded due to primary study (lay summary of primary study). | Focuses experience of maternity care for women with child sexual abuse backgrounds |
| Moore 2017 [337] | Excluded due to lack of CM background. | Experiences of postnatal mental illness. |
| Moran 2004 [338] | Excluded due to lack of CM background. | Childbirth experiences among victims of intimate partner violence. |
| Motley 1995 [339] | Excluded due to topic focus and age (not perinatal). | Examines the characteristics of the mother-child relationship of adult survivors of childhood sexual abuse at the time of the abuse. Children are currently aged over 2 years (mostly teenagers/adults). |
| Moulthrop 1982 [340] | Excluded due to study design. | Attitudes and expectations of mothers who were abused as children. |
| Muchena 2007 [341] | Excluded due to lack of CM background. | Men’s experience of their partners postnatal mental illness. |
| Muldowney 1996 [342] | Excluded due study design and to lack of CM background. | Case study exploring reasons for infant failure to thrive. |
| Murphy 2003 [343] | Excluded due to age (not perinatal), lack of CM background and topic focus. | Making meaning of traumatic child death. |
| Murphy 2004 [344] | Excluded due to lack of CM background. | Experiences of racially diverse mothers who use substances. |
| Murphy 2018 [345] | Excluded due to lack of CM background. | Traumatic birth experiences. |
| Naples 1992 [346] | Excluded due to lack of CM background. | Explores how community work conducted by women contributes to social construction of mothering. |
| Nash 2008 [347] | Excluded due to lack of CM background. | A study describing mothers’ opinions of the crying behaviour of infants under one year of age. |
| Nellsch 1992 [348] | Excluded due to study design. | Cross sectional study examining the effect of childhood experiences, stress, and social support on parental attributions related to the dynamics of physical child abuse |
| Nelson 1990 [349] | Excluded due to lack of CM background. | Experiences of family day care providers. |
| Newby 1993 [350] | Excluded due to topic focus and lack of CM background. | Experiences of grandparents transitioning to parenting role. |
| Newton 2017 [351] | Excluded due to topic focus and to lack of CM background. | Perceptions of child abuse among Aboriginal communities. |
| Ney 2013 [352] | Excluded due to topic focus and to lack of CM background. | Discourse analysis illustrating how child protection discourse dominates in family group conference discussions in the CP context. |
| Nicholls 2007 [353] | Excluded due to lack of CM background and topic focus. | Experiences of childbirth related PTSD. |
| Nicolai 2013 [354] | Excluded due to topic focus and age (not perinatal). | Explores the perceptions of historical trauma among service providers caring for Indigenous children exposed to trauma. |
| Nixon 2013[355] | Excluded due to lack of CM background. | Explores the impact of child protective services on mothers because of concerns of domestic violence. |
| Noble 2007 [356] | Excluded due to lack of CM background, study design and primary study. | Book review of Sheila Kitzinger's 'Birth Crisis'. |
| North 1996 [357] | Excluded due to lack of CM background and topic focus. | Describes experiences of victimisation and violence among homeless women participating in a substance use program. |
| Nyberg 2010 [358] | Excluded due to lack of CM background and topic focus. | Midwifery experiences of encountering PTSD after childbirth. |
| Onyango 2016 [359] | Excluded due to lack of CM background and topic focus. | Experiences of pregnancy after sexual abuse in Democratic Republic of Congo. |
| OReilly 2009 [360] | Excluded due to lack of CM background and topic focus. | Women’s experiences of recovery from childbirth. |
| OReilly 2014 [361] | Excluded due to lack of CM background, age (not perinatal) and topic focus | Parenting experiences and reflections of child protection workers. |
| Pajulo 2012 [362] | Excluded due to lack of CM background and study design. | Descriptive study of mothers involved in residential substance abuse program. |
| Palacios 2008 [363] | Excluded due to age (not perinatal) and lack of CM background. | Interpretive phenomenological study aimed to discover retrospectively the early childbearing experiences of adult, self-identified Native American women who lived on a particular reservation. |
| Palacios 2010 [364] | Excluded due to age (not perinatal) and lack of CM background. | Investigates the previously lived experience of early childbearing among adult Native American women.  . |
| Paluzzi 2007 [365] | Excluded as not primary study. | Discussion article related to understanding the impact of child maltreatment and family violence on the sexual, reproductive, and parenting behaviours of young men. |
| Panchanadewar 2012 [366] | Excluded due to lack of CM background. | Experiences of drug use and parenting among women in substance abuse treatment. |
| Paredes 2001 [367] | Excluded due to study design, age (not perinatal) and lack of CM background. | Descriptive study of mothers of sexually abused children. |
| Paris 2008 [368] | Excluded due to lack of CM background and topic focus. | Experiences of recently arrived immigrant mothers. |
| Paris 2015 [369] | Excluded due to study design and lack of CM background. | Descriptive study of parenting intervention participants |
| Pasalich 2016 [370] | Excluded due to study design. | Descriptive study exploring impact of child abuse history on infant attachment. |
| Peled 2011 [371] | Excluded due to lack of CM background. | Parenting experiences of mothers who are victims of intimate partner violence. |
| Perel 2008 [372] | Excluded due to lack of CM background. | Explores experiences of parenting among men who are violent towards their partners |
| Perry 2015 [373] | Excluded due to lack of CM background and age (not perinatal). | Explores termination of pregnancy experiences among rape victims. |
| Perry 2017 [374] | Excluded due to topic focus, age (not perinatal) and lack of CM background. | Explores experiences of death of parent among homeless adults. |
| Peter 2006 [375] | Excluded due to age (not perinatal) and topic focus. | In-depth exploration of experiences of women who were sexually abused by their mothers. Not specifically related to parenting. |
| Peter 2008 [376] | Excluded due to age (not perinatal) and topic focus. | In-depth exploration of experiences of women who were sexually abused by their mothers. Not specifically related to parenting. |
| Pettersen 2013 [377] | Excluded due to age (not perinatal) and topic focus. | Explores experiences of sexual abuse survivors. |
| Pieh-holder 2012 [378] | Excluded due to lack of CM background and topic focus. | Describes health care experiences of 'underserved' populations. |
| Pierre 2017 [379] | Excluded due to age (not perinatal), topic focus and lack of CM background. | Explores what ways historically traumatic events impact the beliefs, attitudes, perceptions and experiences of Haitian Americans today. |
| Price-Robertson 2012 [380] | Excluded due to primary study. | Review of CSA on fathering. |
| Priddis 2017 [381] | Excluded due to lack of CM background and topic focus. | Abstract regarding experiences of birth trauma. |
| Priddis 2018 [382] | Excluded due to lack of CM background and topic focus. | Experiences of birth trauma among mothers accessing residential parenting services. |
| Psaila 2014 [383] | Excluded due to lack of CM background and topic focus. | Interviews with services providers reading transition from maternity to child health services. |
| Raj 2011 [384] | Excluded due to lack of CM background. | Examines experiences of perinatal abuse from in-laws and husbands in India. |
| Rajiva 2013 [385] | Excluded due to lack of CM background and topic focus. | Explores shifting work/family dynamics among female South Asian diaspora in Canada. |
| Rees 2015 [386] | Excluded due to lack of CM background and topic focus. | Explores role of IPV and explosive anger to reduce harsh parenting among women in East Timor. |
| Reeves 2018 [387] | Excluded due to age (not perinatal), lack of CM background and topic focus. | Describes the healthcare experiences and strategies of women survivors of violence. |
| Remez 2014 [388] | Excluded due to lack of CM background and study design. | Therapist reflections of working with traumatized mothers and their babies in a residential drug rehabilitation facility. |
| Renker 2002 [389] | Excluded due to lack of CM background. | Explores adolescents’ experiences of abuse in the year before and during pregnancy. |
| Renner 2006 [390] | Excluded due to study design. | Descriptive study (survey) investigating intimate partner violence and different forms of child maltreatment within and across childhood and adulthood for a high-risk group of women. |
| Rice 2013 [391] | Excluded due to lack of CM background. | Midwives experiences of witnessing traumatic birth. |
| Richards 2015 [392] | Excluded due to lack of CM background and topic focus. | Experiences of mothers who have had a loss from a twin pregnancy and subsequently continued visiting hospital whilst their surviving twin was cared for. |
| Rigg 2017 [393] | Excluded due to lack of CM background and topic focus. | Explores the reasons why women choose to give birth at home with an unregulated birth worker (UBW) from the perspective of women and UBWs. |
| Rizo 2016 [394] | Excluded due to lack of CM background and topic focus. | Investigation of a mandated parenting and safety program for system-involved female IPV survivors. |
| Rollans 2013 [395] | Excluded due to lack of CM background. | Describes women’s experience of psychosocial assessment and screening for child maltreatment and other issues. |
| Roman 2008 [396] | Excluded due to age (not perinatal) and topic focus. | Explores role of interpersonal relationships in women who have thrived despite childhood maltreatment. |
| Rose 1992 [397] | Excluded due to study design (case study, first person). | One woman's story of the effects of childhood sexual abuse on childbearing |
| Rose 2001 [398] | Excluded due to lack of CM background, study design and topic focus. | Midwives experience of working in the Philippines. |
| Roseth 2011 [399] | Excluded due to study design. | Case study exploring how the experience of incest intertwines with the experience of postpartum depression. |
| Rossman 2005 [400] | Excluded due to study design and lack of CM background. | Descriptive study exploring relationship between parenting styles and inconsistencies to adaptive functioning for children in conﬂictual and violent families. |
| Roth-howe 2007 [401] | Excluded due to topic focus and to lack of CM background. | Personal journey toward a better understanding and integration of parents’ Holocaust experiences, and of the losses they and their extended families suffered under Nazi rule. |
| Sachs 1995 [402] | Excluded due to lack of CM background. | Describes psychosocial and environmental contexts affecting the lives of low-income, single mothers and to explore their everyday coping strategies. |
| Salberg 2015 [403] | Excluded due to study design and age (not perinatal). | Utilizing examples from her own life, the author illustrates how attachment patterns are a primary mode of transmission of trauma. |
| Salomonsson 2013 [404] | Excluded due to study design and lack of CM background. | Case study of a PND mother in psychoanalysis and mother–infant dyad in psychotherapy. |
| Salter 2014 [405] | Excluded due to topic focus and lack of CM background. | Experiences of adult women with histories of childhood sexual abuse and/or domestic violence in AOD treatment. |
| Salvi 2008 [406] | Excluded due to age (not perinatal). | A dissertation of the understanding of the lived experiences of women who had seen abuse pass from earlier generations and elaborate a theory to account for intergenerational transmission of childhood maltreatment, using the Assimilation Model. |
| Santoro 2018 [407] | Excluded due to topic focus and lack of CM background. | Narratives of birth trauma in the postnatal period. |
| Saunders 2015 [408] | Excluded due to primary study. | Editorial for journal edition around memories of abuse. |
| Sawyer 2013 [409] | Excluded due to lack of CM background and topic focus. | Parents’ experiences and satisfaction with care during the birth of their very preterm baby. |
| Schechter 2003 [410] | Excluded due to study design. | A mother-infant case-study involving intergenerational violent trauma and pseudo seizures across three generations. |
| Schechter 2017 [411] | Excluded due to study design and lack of CM background. | Case study exploring challenges in the treatment of women suffering from disturbances in maternal identification. |
| Schimmenti 2012 [412] | Excluded due to study design, age (not perinatal) and topic focus. | A clinical vignette of a patient who suﬀered emotional neglect and intense role reversal during his childhood to elucidate how developmental trauma can dramatically aﬀect the patient’s personality and behaviours, and ultimately lead to negative expectations towards interpersonal relationships, disturbing feelings of shame, and a sense of a defective self. |
| Schlesinger 2012 [413] | Excluded due to study design, primary study and topic focus. | Commentary on Paper by Laurel Moldawsky Silber relating to the 'history of ghosts'. |
| Schleske 1999 [414] | Excluded due to study design. | Case of three women with a focus on continuity between fantasies about the imaged child during pregnancy and the quality of the real early mother-child relationship. |
| Schroll 2013 [415] | Excluded due to lack of CM background. | Describes abuse experiences in postnatal care. |
| Schumacher 2008 [416] | Excluded due to study design. | Case study and commentary about the need to support mothers with personality disorders WITH their children. |
| Seamans 2007 [417] | Excluded due to lack of CM background and topic focus. | Examined female domestic violence offenders referred for treatment in batterers’ intervention programs in a major metropolitan area. |
| Searle 2017 [418] | Excluded due to lack of CM background. | Explores understandings of trauma-informed care with queer birthing women in a rural context. |
| Setterberg 2017 [419] | Excluded due to study design and lack of CM background. | Mixed methods study of risk factors of antenatal anxiety and depression focusing particularly on maternal representations of the relationship towards the foetus and her own parents during pregnancy and the early postpartum period. |
| Shahram 2017 [420] | Excluded due to lack of CM background. | As part of life history interviews, 17 young pregnant-involved Indigenous women with experiences with substances completed a participant-generated mapping activity CIRCLES (Charting Intersectional Relationships in the Context of Life). |
| Shahram 2017 [421] | Excluded due to lack of CM background. | Understanding the life histories of pregnant-involved young Aboriginal women with substance use experiences in three Canadian cities. |
| Sheen 2016 [422] | Excluded due to lack of CM background and topic focus. | Midwives experiences of traumatic perinatal events. |
| Sheen 2016 [423] | Excluded due to lack of CM background and topic focus. | Characteristics of perinatal events perceived as traumatic by midwives. |
| Sherman 2016 [424] | Excluded due to age (not perinatal) and lack of CM background. | Explores the impact of PTSD among war veterans on their functioning as parents. |
| Shimoda 2018 [425] | Excluded due to lack of CM background. | Midwives’ respect and disrespect of women during facility-based childbirth in urban Tanzania. |
| Siegel 2011 [426] | Excluded due to study design. | Parent-infant psychotherapy and art therapy in the treatment of intergenerational separation individuation struggles. |
| Sigurdardottir 2012 [427] | Excluded due to age (not perinatal) and topic focus. | Phenomenological study of the consequences of childhood sexual abuse for Icelandic men’s health and wellbeing. |
| Silva-Martinez 2017 [428] | Excluded due to age (not perinatal) and lack of CM background. | Explores experiences among immigrant Latina survivors of intimate partner violence. |
| Simkin 2010 [429] | Excluded due to not being a primary study. | Book review of "Survivor Moms: Women’s Stories of Birthing, Mothering and Healing after Sexual Abuse". |
| Simpkins 2006 [430] | Excluded due to primary study and study design. | Literature review of the effects of sexual abuse on antenatal care and childbearing. |
| Skibniewski 2017 [431] | Excluded due to primary study and lack of CM background. | Literature review of studies involving women with major mental illness and their children. |
| Skinner 2010 [432] | Excluded due to age (not perinatal), primary study and study design. | Personal account of a survivor’s experience of perinatal care. |
| Skinner 2018 [433] | Excluded due to lack of CM background . | Explores perineal floor trauma. |
| Slesnick 2013 [434] | Excluded due to study design and lack of CM background. | Explores homeless mothers' desires for treatment in several commonly reported problem areas including substance use, parenting, depressive symptoms/mood, physical health, and childhood abuse history. |
| Smid 2010 [435] | Excluded due to lack of CM background. | Explores how homeless street youth experience their pregnancies. |
| Smith 2009 [436] | Excluded due to topic focus and lack of CM background. | Explores chronic sorrow as a relapse trigger for relapse in substance use treatment. |
| Smith 2004 [437] | Excluded due to lack of CM background. | Explores experiences of addicted incarcerated parents whose children are cared for by relatives. |
| SmithBattle 2008 [438] | Excluded due to lack of CM background and study design (case study of multiple generations). | A multigenerational longitudinal study of teen mothering and the continuity and discontinuity of intergenerational parenting traditions and caregiving legacies. |
| SmithLester 2017 [439] | Excluded due to age (not perinatal), lack of CM background and topic focus. | Investigates intergenerational continuities and discontinuities in parenting traditions. |
| SmithMK 2003 [440] | Excluded due to study design, age (not perinatal) and topic focus. | Narratives of recovery stories that heal intergenerational trauma. |
| SmithStover 2013 [441] | Excluded due to lack of CM background. | Explores the ways in which fathers with co-occurring IPV and SA describe the parenting of their own parents and how it is related to the ways they parent. |
| Snajder-Murray 2011 [442] | Excluded due to lack of CM background. | A qualitative study to gain a better understanding of homeless mothers’ perceptions of service providers. |
| Song 2013 [443] | Excluded due to topic focus and lack of CM background. | Explores homeless mothers’ perceptions of service providers. |
| Song 2014 [444] | Excluded due to topic focus and lack of CM background. | Explores parenting experiences of former child soldiers. Mean age of current children 5 years. |
| Souza 2009 [445] | Excluded due to lack of CM background and topic focus. | Investigates women's experiences related to the burden of severe maternal morbidity. |
| Spangaro 2011 [446] | Excluded due to lack of CM background. | Explores the conditions under which a group of women recruited from antenatal, mental health, and substance abuse services disclose abuse in response to routine screening for IPV and their constructions of the impact of routine screening. |
| Spangaro 2016 [447] | Excluded due to lack of CM background. | Qualitative configurational analysis of factors and pathways influencing women's decisions to disclose intimate partner violence in the antenatal care setting. |
| Springer-Kremser 2003 [448] | Excluded due to study design. | Case study describing characteristics of the psychic structure of patients who presented symptoms of deliberate self-harm and of misusing and mistreating their children. |
| Stanley 2012 [449] | Excluded due to lack of CM background. | Describes views of parents and young people who had experienced domestic violence. |
| Stanley 2016 [450] | Excluded due to lack of CM background. | Explores health and healthcare needs of young people trafficked into the UK. |
| Stapleton 2013 [451] | Excluded due to lack of CM background. | Explores the cross-cultural application of the Edinburgh Postnatal Depression Scale (EPDS) and the difﬁculties associated with administration to women from refugee backgrounds. |
| Stern 1995 [452] | Excluded due to lack of CM background. | Investigates adult children of Holocaust survivors' subjective experience of parenthood. |
| Stern 2014 [453] | Excluded due to lack of CM background. | Presents clinical work with a woman who experienced trauma in both her childhood and adult life and who demonstrated poor reﬂective functioning. |
| Stewart 2015 [454] | Excluded due to lack of CM background. | Examines challenges faced by refugee new parents from Africa in Canada. |
| Stidham 2009 [455] | Excluded due to age (not perinatal), topic focus and lack of CM background. | Explores how helping others affects coping or recovery from sexual violence. |
| Stockl 2013 [456] | Excluded due to lack of CM background. | Investigates women’s perceptions on how their pregnancy inﬂuences the context in which intimate partner violence occurs. |
| Strong 2010[457] | Excluded due to study design (article is review and not original study about grand parenting and trauma). | Development of a model of treatment for trauma, attachment, and family therapy with grand families. |
| Tait 2013 [458] | Excluded due to age (not perinatal), lack of CM background and topic focus. | Examines the life history of a First Nations woman, and illustrates the intergenerational role that government policies play in the lives of impoverished Indigenous women and their families. |
| Tapias 2006 [459] | Excluded due to study design, lack of CM background and topic focus. | Explores embodied manifestations of distress across generations to illustrate the subtle articulations between the political restructuring of the Bolivian state and the private anxieties women experience under enduring political and economic instability. |
| Taylor 2015 [460] | Excluded due to age (not perinatal), topic focus and lack of CM background. | Investigates how homeless may affect preschool choices. |
| Telfeyan 2007 [461] | Excluded due to lack of CM background. | Explores the effects of maternal substance abuse and the impact that treatment and recovery have upon mother-infant attachment patterns. |
| Thomas 2008 [462] | Excluded due to age (not perinatal) and topic focus. | A narrative study of thriving adult female survivors of childhood maltreatment to discover how they had achieved success. |
| Thomas 2012 [463] | Excluded due to age (not perinatal). | Explores the role of anger in the trajectory of healing from childhood maltreatment. |
| Thomas 2018 [464] | Excluded due to lack of CM background. | Explores Responsible Fathering Group participants' understanding of DV and its impact on the children exposed to it. |
| Tilley 2004 [465] | Excluded due to lack of CM background. | Investigates critical developmental periods, experiences, and events in women’s lives associated with violence. |
| Tingberg 2008 [466] | Excluded due to topic focus and lack of CM background. | Explores nurse’s experiences in the clinical care of children experiencing abuse. |
| Titsworth 2009 [467] | Excluded due to lack of CM background. | Explores the impact of mothering practices as a way to influence, contribute to and empower single mothers and their children's potential for educational persistence. |
| Tol 2010 [468] | Excluded due to topic focus and lack of CM background. | Examines the health care system in relation to communal violence-related psychosocial wellbeing in Poso, Indonesia. |
| Torchalla 2015 [469] | Excluded due to lack of CM background. | Explores themes and subjective perspectives of trauma and gender-based violence in women who lived in an impoverished neighbourhood and struggled with substance use during pregnancy and early motherhood. |
| Tracey 1996 [470] | Excluded due to study design. | Case study narrative of a first-time father with a son born seven weeks early by Caesarean section. |
| Trad 1996 [471] | Excluded due to study design. | A case history of a 16 year old child-abused mother of a 3 month old girl. |
| Ueno 2004 [472] | Excluded due to lack of CM background. | Describes the practical knowledge that public health nurses use in identifying and providing assistance to the mothers of child abuse victims. |
| Veale 2013 [473] | Excluded due to lack of CM background and topic focus. | Examines how young mothers formerly involved with armed groups transformed their identity and membership within communities of return through drama, songs and poetry, and engagement in social actions. |
| Veltkamp 2017 [474] | Excluded due to lack of CM background. | Investigates how healthcare professionals juggle assessing risk of abuse and neglect and managing risk of ‘poor parenting’ more broadly. |
| Virokannas 2011 [475] | Excluded due to lack of CM background. | Discusses the self-conceptions of the women recovering from using illegal drugs as they related their experiences with social workers and the child welfare system. |
| Volpe 2017 [476] | Excluded due to lack of CM background. | Examines the feasibility of Narrative Exposure Therapy among adolescents at-risk for PTSD and depression. |
| Waldrop 2000 [477] | Excluded due to age (not perinatal) and lack of CM background. | Explores the issues that grandparents face while raising their grandchildren. |
| Walker 2007 [478] | Excluded due to lack of CM background. | A phenomenological case study of the lived experiences of a mother and daughter who have exhibited resiliency factors in the process of recovery from childhood sexual abuse, incest, and trauma. |
| Walker 2017 [479] | Excluded due to age (not perinatal) and topic focus. | A focus on sex work among non-nationals in Zimbabwe, and influence this has on parenting and perinatal care |
| Walters 2001 [480] | Excluded due to lack of CM background and age (not in perinatal period). | Explores the experience of repeated homelessness in the lives of young women. |
| Wamoyi 2015 [481] | Excluded due to lack of CM background and topic focus. | Explores the structural inﬂuence of family and parenting on young people’s sexual and reproductive health in rural northern Tanzania. |
| Wangerin 1996 (1994) [482] | Excluded due to age (not perinatal). | Examines patterns of maternal protectiveness regarding sexual abuse in a sample of mothers with a history of childhood sexual abuse. Participants are parents with a child 3 years of age or over. |
| Wapinsky 2016 [483] | Excluded due to lack of CM background and topic focus. | Explores the perspectives of pregnant women using opiates about the circumstances in their lives and/or the nature of the treatments offered to them that may present barriers to engaging in treatment as well as to understand factors that might motivate them to seek substance use treatment. |
| Ward 2005 [484] | Excluded due to age (not perinatal) and topic focus. | A study of children’s views of the care system in England. |
| Watt 2017 [485] | Excluded due to age (not perinatal) and lack of CM background. | Explores the impact of sexual trauma on HIV care engagement: among female patients with Trauma Histories in South Africa. |
| Werner-Wilson 2000 [486] | Excluded due to lack of CM background. | Explores why, despite harsh life circumstances, some victims of intimate violence survive and later thrive after experiencing trauma. |
| White 2007 [487] | Excluded due to lack of CM background. | Explores the phenomenon of post-traumatic stress following childbirth among fathers witnessing a traumatic birth. |
| Wijnberg 1999 [488] | Excluded due to lack of CM background and topic focus. | Explores role of chronic stressors, commonly called “hassles”, in the lives of poor rural women. |
| Willey 2003 [489] | Excluded due to lack of CM background. | Explores the experiences of 8 women about changes in their own sexuality during pregnancy. |
| Williams 2009[490] | Excluded due to age (not perinatal). | An exploration on how past events influence current traumatic stress among mothers experiencing homelessness. |
| Williams 2015 [491] | Excluded due to lack of CM background. | Explores life experiences among mothers who were homeless and living in transitional housing. |
| Williamson 2018 [492] | Excluded due to age (not perinatal) and lack of CM background. | Explores caregivers’ experiences of accessing and interacting with public services post-trauma and perceptions of needed improvements to public services in a LMIC context. |
| Wilson-Mitchell 2014 [493] | Excluded due to lack of CM background. | Explores the experiences and the impact of pregnancy on pregnant adolescent psychological health. |
| Woodhouse 1992 [494] | Excluded due to lack of CM background and topic focus. | Life history study to illuminate the lives of women who are substance abusers. |
| Wright 2007 [495] | Excluded due to age (not perinatal). | Resolution of the trauma of childhood sexual abuse (CSA), and the current adjustment of 60 adult female CSA survivors (all mothers). |
| Wuest 2010 [496] | Excluded due to topic focus and age (not perinatal). | Explores daughters’ obligation to care in the context of past abuse. |
| Xie 2017 [497] | Excluded due to age (not perinatal) and to lack of CM background. | Explores parents' intentions of reporting their own children's CSA experiences to authorities as well as their reporting willingness when they become aware of possible CSA cases happening to children in other families in China. |
| Yaroslawitz 2015 [498] | Excluded due to lack of CM background and age (not perinatal). | Describe the lived experience of holocaust survivors and captures the perceptions of the impacts on family health. |
| Yi 2008 [499] | Excluded due to lack of CM background. | Investigates psychosocial factors that Black women think should be addressed in prenatal care assessment to develop a Prenatal Event History Calendar to assess these factors. |
| Zanoni 2014 [500] | Excluded due to lack of CM background. | Describes stereotypes of fathers involved with child protection regarding limited opportunities for change and explores how these stereotypes are supported in a sample of Australian fathers involved with child protection services. |
| Zanoni 2014 [501] | Excluded due to lack of CM background. | Describes child protection fathers' experiences of childhood, intimate partner violence and parenting. |
| Zeanah 2006 [502] | Excluded due to lack of CM background. | Describes the role of the nurse in the NFP and present results of focus groups with experienced NFP nurses regarding their perspectives, challenges, and rewards in conducting this work. |
| Zlotnick 1999 [503] | Excluded due to study design and lack of CM background. | Descriptive study comparing homeless women who had childhood histories of foster care or other out-of-home placement to those who have not. |

# References

1. Abboud L, Liamputtong P. When pregnancy fails: Coping strategies, support networks and experiences with health care of ethnic women and their partners. Journal of Reproductive and Infant Psychology. 2005;23(1):3-18. doi: <http://dx.doi.org/10.1080/02646830512331330974>.

2. Abraham RJ. Meaning making in adult survivors of complex trauma: A narrative analysis. Dissertation Abstracts International: Section B: The Sciences and Engineering. 2018;78(8-B(E)).

3. Abram A. Motherhood as a transformative experience for first-time mothers. Dissertation Abstracts International Section A: Humanities and Social Sciences. 2008;69(3-A):1154.

4. Aho KL-T. The healing is in the pain: Revisiting and re-narrating trauma histories as a starting point for healing. Psychology and Developing Societies. 2014;26(2):181-212. doi: <http://dx.doi.org/10.1177/0971333614549139>.

5. Allbaugh LJ, O'Dougherty Wright M, Atkins Seltmann L. An exploratory study of domains of parenting concern among mothers who are childhood sexual abuse survivors. J Child Sex Abuse. 2014;23(8):885-99. doi: <https://dx.doi.org/10.1080/10538712.2014.960636>.

6. Allen S, Flaherty C, Ely GE. Throwaway moms: maternal incarceration and the criminalization of female poverty. Afilia. 2010;25(2):160.

7. Allnock D, Miller P. No one noticed, no one heard : a study of disclosures of childhood abuse. NSPCC, 2013.

8. Almqvist K, Broberg AG. Young children traumatized by organized violence together with their mothers--the critical effects of damaged internal representations. Attach Hum Dev. 2003;5(4):367-80; discussion 409-14.

9. Amin P, Buranosky R, Chang JC. Physicians' Perceived Roles, as Well as Barriers, Toward Caring for Women Sex Assault Survivors. Women's Health Issues. 2017;27(1):43-9. doi: 10.1016/j.whi.2016.10.002.

10. Anderson KM, Danis FS. Adult daughters of battered women: resistance and resilience in the face of danger. Affilia: Journal of Women & Social Work. 2006;21(4):419-32.

11. Andrews M. Memories of mother: Counter-narratives of early maternal influence. Narrative Inquiry. 2002;12(1):7-27. doi: <http://dx.doi.org/10.1075/ni.12.1.04and>.

12. Annand S. From the beginning: Exploring the prenatal and perinatal narratives of mothers court-mandated to substance abuse treatment. Dissertation Abstracts International: Section B: The Sciences and Engineering. 2018;78(10-B(E)).

13. Arditti J, Few A. Maternal distress and women's reentry into family and community life. Fam Process. 2008;47(3):303.

14. Babcock D. The darkness isn't so dark anymore. Motherhood, mental illness and recovery: Stories of hope. Cham, Switzerland: Springer International Publishing; Switzerland; 2014. p. 225-9.

15. Bacchus L, Mezey G, Bewley S. Experiences of seeking help from health professionals in a sample of women who experienced domestic violence. Health Soc Care Community. 2003;11(1):10-8.

16. Bacchus LJ, Bullock L, Sharps P, Burnett C, Schminkey D, Buller AM, et al. 'Opening the door': A qualitative interpretive study of women's experiences of being asked about intimate partner violence and receiving an intervention during perinatal home visits in rural and urban settings in the USA. Journal of Research in Nursing. 2016;21(5-6):345-64. doi: <http://dx.doi.org/10.1177/1744987116649634>.

17. Backett‐Milburn K, Jackson S. Children's concerns about their parents' health and well-being: researching with ChildLine Scotland. Child Soc. 2012;26:381-93.

18. Baker LJ. Multigenerational sexual abuse: A cognitive developmental approach to understanding mothers' perceptions of self, parenthood, and change. Dissertation Abstracts International. 1993;54(6-A):2051.

19. Baker LJ. Multigenerational sexual abuse: A cognitive developmental approach to understanding mothers in treatment. Journal of Adult Development. 2001;8(1):51-9. doi: <http://dx.doi.org/10.1023/A:1026449805197>.

20. Ball J. Indigenous Fathers' Involvement in Reconstituting "Circles of Care". Am J Community Psychol. 2010;45(1-2):124-38. doi: 10.1007/s10464-009-9293-1.

21. Banwell C, Bammer G. Maternal habits: Narratives of mothering, social position and drug use. Int J Drug Policy. 2006;17(6):504-13. doi: 10.1016/j.drugpo.2006.09.005.

22. Barclay L, Donovan J, Genovese A. Men's experiences during their partner's first pregnancy: a grounded theory analysis. Aust J Adv Nurs. 1996;13(3):12-24.

23. Barnes S. Historical trauma's impact on dating violence and pregnancy among urban native Americans. Dissertation Abstracts International: Section B: The Sciences and Engineering. 2018;79(1-B(E)).

24. Barrett E, Denieffe S, Bergin M, Gooney M. An exploration of paediatric nurses' views of caring for infants who have suffered nonaccidental injury. J Clin Nurs. 2017;26(15-16):2274-85. doi: <https://dx.doi.org/10.1111/jocn.13439>.

25. Bateman L, Jones C, Jomeen J. A Narrative Synthesis of Women's Out-of-Body Experiences During Childbirth. J Midwifery Womens Health. 2017;62(4):442-51. doi: <https://dx.doi.org/10.1111/jmwh.12655>.

26. Baxter JMS, Kavanaugh K, Vonderheid S. Exploring the Lived Experience of Trauma Among Obstetric Registered Nurses. JOGNN: Journal of Obstetric, Gynecologic & Neonatal Nursing. 2014;43(Supp 1):S83-4. doi: 10.1111/1552-6909.12438.

27. Bayes S, Fenwick J, Hauck Y. A qualitative analysis of women's short accounts of labour and birth in a Western Australian public tertiary hospital. J Midwifery Women Health. 2008;53(1):53-61. doi: 10.1016/j.jmwh.2007.07.021.

28. Bayes S, Fenwick J, Hauck Y. 'Off everyone's radar': Australian women's experiences of medically necessary elective caesarean section. Midwifery. 2012;28(6):E900-E9. doi: 10.1016/j.midw.2012.01.004.

29. Beck CT. Benefits of participating in Internet interviews: Women helping women. Qual Health Res. 2005;15(3):411-22. doi: 10.1177/1049732304270837.

30. Beck CT. Pentadic cartography: mapping birth trauma narratives. Qual Health Res. 2006;16(4):453-66.

31. Beck CT, Watson S. Impact of birth trauma on breast-feeding - A tale of two pathways. Nurs Res. 2008;57(4):228-36. doi: 10.1097/01.nnr.0000313494.87282.90.

32. Beck CT. An adult survivor of child sexual abuse and her breastfeeding experience: a case study. MCN Am J Matern Child Nurs. 2009;34(2):91-7. doi: <https://dx.doi.org/10.1097/01.NMC.0000347302.85455.c8>.

33. Beck CT, LoGiudice J, Gable RK. A mixed-methods study of secondary traumatic stress in certified nurse-midwives: shaken belief in the birth process. J Midwifery Womens Health. 2015;60(1):16-23. doi: <https://dx.doi.org/10.1111/jmwh.12221>.

34. Belknap RA, Cruz N. When I was in my home I suffered a lot: Mexican women's descriptions of abuse in family of origin. Health Care Women Int. 2007;28(5):506-22.

35. Bell PW. The relational patterns between African American mothers under correctional supervision and maternal caregiving grandmothers. Dissertation Abstracts International Section A: Humanities and Social Sciences. 2008;69(4-A):1538.

36. Ben-Amitay G, Buchbinder E, Toren P. Understanding sexual revictimization of women through metaphors: A qualitative research. Journal of Aggression, Maltreatment and Trauma. 2015;24(8):914-31. doi: <http://dx.doi.org/10.1080/10926771.2015.1069773>.

37. Benjamin LR, Benjamin R, Rind B. Dissociative mothers' subjective experience of parenting. Child Abuse Negl. 1996;20(10):933-42.

38. Benjamin LR, Benjamin R, Rind B. The parenting experiences of mothers with dissociative disorders. J Marital Fam Ther. 1998;24(3):337-54.

39. Benoit D, Madigan S, McKibbon A, Vaillancourt K. Pregnant adolescents' unresolved trauma/mourning (using adapted Adult Attachment Interview): Links to self-report of socio-emotional adjustment. Infant Ment Health J. 2010;31(3):164-.

40. Bertini KA. Marital status and midlife: Perceptions of early parenting and perceptions of self. Dissertation Abstracts International: Section B: The Sciences and Engineering. 1996;56(10-B):5758.

41. Bick D, Howard LM, Oram S, Zimmerman C. Maternity care for trafficked women: Survivor experiences and clinicians' perspectives in the United Kingdom's National Health Service. PLoS ONE. 2017;12(11):e0187856. doi: <https://dx.doi.org/10.1371/journal.pone.0187856>.

42. Blakey JM, Hatcher SS. Trauma and substance abuse among child welfare involved African American mothers: a case study. Journal of Public Child Welfare. 2013;7(2):194-216.

43. Boustani MM, Frazier SL, Hartley C, Meinzer M, Hedemann E. Perceived Benefits and Proposed Solutions for Teen Pregnancy: Qualitative Interviews With Youth Care Workers. Am J Orthopsychiatry. 2015;85(1):80-92. doi: 10.1037/ort0000040.

44. Brazelton JF. African American women looking back: Making meaning of the disclosure process of incest survivors across the life course. Dissertation Abstracts International Section A: Humanities and Social Sciences. 2011;72(2-A):738.

45. Breckenridge J. "Speaking of mothers. . ."How does the literature portray mothers who have a history of child sexual abuse? J Child Sex Abuse. 2006;15(2):57-74.

46. Brookes H, Coster D. Baby Steps in a prison context: parents perspectives. London: NSPCC, 2014.

47. Brown EM. The transmission of trauma through caretaking patterns of behavior in Holocaust families: Re-enactments in a facilitated long-term second-generation group. Smith College Studies in Social Work. 1998;68(3):267-85. doi: <http://dx.doi.org/10.1080/00377319809517531>.

48. Brown G, Brady G, Letherby G. Young mothers' experiences of power, control and violence within intimate and familial relationships. Child Care in Practice. 2011;17(4):359-74.

49. Buchanan F, Power C, Verity F. Domestic violence and the place of fear in mother/baby relationships: "what was I afraid of ? Of making it worse.". J Interpers Violence. 2013;28(9):1817-38. doi: <https://dx.doi.org/10.1177/0886260512469108>.

50. Buchbinder E. Motherhood of Battered Women: The Struggle for Repairing the Past. Clinical Social Work Journal. 2004;32(3):307-26. doi: <http://dx.doi.org/10.1023/B:CSOW.0000035110.10602.ec>.

51. Bundy-Fazioli K, Hamilton TA. A qualitative study exploring mothers' perceptions of child neglect. Child & Youth Services. 2013;34(3):250-66. doi: <http://dx.doi.org/10.1080/0145935X.2013.826034>.

52. Burnett C, Schminkey D, Milburn J, Kastello J, Bullock L, Campbell J, et al. Negotiating Peril: The Lived Experience of Rural, Low-Income Women Exposed to IPV During Pregnancy and Postpartum. Violence Against Women. 2016;22(8):943-65. doi: 10.1177/1077801215614972.

53. Burnette CE. Historical oppression and indigenous families: Uncovering potential risk factors for indigenous families touched by violence. Family Relations: An Interdisciplinary Journal of Applied Family Studies. 2016;65(2):354-68. doi: <http://dx.doi.org/10.1111/fare.12191>.

54. Burnette CE, Renner LM. A pattern of cumulative disadvantage: Risk factors for violence across indigenous women's lives. British Journal of Social Work. 2017;47(4):1166-85.

55. Butler JR, Burton LM. Rethinking teenage childbearing: Is sexual abuse a missing link? Family Relations: An Interdisciplinary Journal of Applied Family Studies. 1990;39(1):73-80. doi: <http://dx.doi.org/10.2307/584952>.

56. Cahalane H, Parker G, Duff S. Treatment implications arising from a qualitative analysis of letters written by the nonoffending partners of men who have perpetrated child sexual abuse. J Child Sex Abuse. 2013;22(6):720-41. doi: <https://dx.doi.org/10.1080/10538712.2013.811138>.

57. Caliso JA. A psychological study of mothers who do not physically abuse their children despite histories of physical abuse in their own childhoods. Dissertation Abstracts International. 1986;47(3-B):1265-6.

58. Callaghan JEM, Gambo Y, Fellin LC. Hearing the silences: Adult Nigerian women’s accounts of ‘early marriages’. Feminism & Psychology. 2015;25(4):506-27. doi: 10.1177/0959353515590691.

59. Calvete E, Orue I, Bertino L, Gonzalez Z, Montes Y, Padilla P, et al. Child-to-parent violence in adolescents: The perspectives of the parents, children, and professionals in a sample of Spanish focus group participants. Journal of Family Violence. 2014;29(3):343-52. doi: <http://dx.doi.org/10.1007/s10896-014-9578-5>.

60. Carpiano RM. Long roads to tall mountains: the impact of motherhood on the recovery and health of domestic abuse survivors. Health Care Women Int. 2002;23(5):442-9.

61. Cavanaugh CE, Harper B, Classen CC, Palesh O, Koopman C, Spiegel D. Experiences of Mothers Who Are Child Sexual Abuse Survivors: A Qualitative Exploration. J Child Sex Abuse. 2015;24(5):506-25. doi: <https://dx.doi.org/10.1080/10538712.2015.1042186>.

62. Cecchet SJ, Thoburn J. The psychological experience of child and adolescent sex trafficking in the United States: Trauma and resilience in survivors. Psychological Trauma: Theory, Research, Practice, and Policy. 2014;6(5):482-93. doi: <http://dx.doi.org/10.1037/a0035763>.

63. Cerulli C, Poleshuck E, Raimondi C, Veale S, Chin N. 'What Fresh Hell Is This?' Victims of Intimate Partner Violence Describe Their Experiences of Abuse, Pain, and Depression. Journal of Family Violence. 2012;27(8):773-81. doi: 10.1007/s10896-012-9469-6.

64. Chadwick RJ, Cooper D, Harries J. Narratives of distress about birth in South African public maternity settings: a qualitative study. Midwifery. 2014;30(7):862-8. doi: <https://dx.doi.org/10.1016/j.midw.2013.12.014>.

65. Chambers J. An analysis of the factors that contribute to a positive birthing experience for women with a history of sexual trauma. Dissertation Abstracts International: Section B: The Sciences and Engineering. 2010;70(10-B):6543.

66. Chanmugam A. Perspectives on US domestic violence emergency shelters: what do young adolescent residents and their mothers say? Child Care in Practice. 2011;17(4):393-415.

67. Charbonneau-Dahlen BK. Giving voice to historical trauma through storytelling: The impact of boarding school experience on American Indians: Florida Atlantic University; 2010.

68. Chien MH. Deciding to disclose childhood sexual abuse - The experiences of Taiwanese women with their non-offending mothers. Asia Pac J Soc Work Dev. 2005;15(2):19-39. doi: 10.1080/21650993.2005.9755978.

69. Chilton M, Knowles M, Rabinowich J, Arnold KT. The relationship between childhood adversity and food insecurity: 'It's like a bird nesting in your head'. Public Health Nutr. 2015;18(14):2643-53. doi: <https://dx.doi.org/10.1017/S1368980014003036>.

70. Choi KR, Seng JS. Pilot for Nurse-Led, Interprofessional In-Service Training on Trauma-Informed Perinatal Care. J Contin Educ Nurs. 2015;46(11):515-21. doi: <https://dx.doi.org/10.3928/00220124-20151020-04>.

71. Choi KW, Watt MH, Skinner D, Kalichman SC, Sikkema KJ. "Wine you get every day, but a child you can't replace": The perceived impact of parental drinking on child outcomes in a South African township. Journal of Child & Adolescent Mental Health. 2015;27(3):173-87. doi: <https://dx.doi.org/10.2989/17280583.2015.1113974>.

72. Clark E, Smythe L. The Effects of Childhood Sexual Abuse on Labour and Birthing: an Exploration to Assist Midwives. New Zealand College of Midwives Journal. 2011;(45):21-4.

73. Clarke PJ. Saskatchewan aboriginal women's postpartum depressive experiences: A qualitative exploration. Dissertation Abstracts International: Section B: The Sciences and Engineering. 2010;71(1-B):653.

74. Cleveland LM, Bonugli RJ, McGlothen KS. The Mothering Experiences of Women With Substance Use Disorders. ANS Adv Nurs Sci. 2016;39(2):119-29. doi: 10.1097/ans.0000000000000118.

75. Coates R, Ayers S, de Visser R. Women's experiences of postnatal distress: a qualitative study. Bmc Pregnancy and Childbirth. 2014;14:14. doi: 10.1186/1471-2393-14-359.

76. Coates D, Davis E, Campbell L. The experiences of women who have accessed a perinatal and infant mental health service: A qualitative investigation. Advances in Mental Health. 2017;15(1):88-100. doi: <http://dx.doi.org/10.1080/18387357.2016.1242374>.

77. Cogan JC. The consumer as expert: Women with serious mental illness and their relationship-based needs. Psychiatr Rehabil J. 1998;22(2):142-54. doi: 10.1037/h0095253.

78. Cohen T. Motherhood among incest survivors. Child Abuse Negl. 1995;19(12):1423-9.

79. Cole PM, Woolger C, Power TG, Smith KD. Parenting difficulties among adult survivors of father-daughter incest. Child Abuse Negl. 1992;16(2):239-49.

80. Coleman H, Collins D. The voices of parents: A qualitative study of a family preservation program. Child & Youth Care Forum. 1997;26(4):261-78. doi: <http://dx.doi.org/10.1007/BF02589419>.

81. Collins ME. Parents' perceptions of the risk of child sexual abuse and their protective behaviors: Findings from a qualitative study. Child Maltreat. 1996;1(1):53-64. doi: <http://dx.doi.org/10.1177/1077559596001001006>.

82. Conn A-M, Szilagyi MA, Jee SH, Manly JT, Briggs R, Szilagyi PG. Parental perspectives of screening for adverse childhood experiences in pediatric primary care. Fam Syst Health. 2018;36(1):62-72. doi: <http://dx.doi.org/10.1037/fsh0000311>.

83. Coyer SM. Women in recovery discuss parenting while addicted to cocaine. MCN Am J Matern Child Nurs. 2003;28(1):45-9.

84. Crawford S. Life satisfaction in adulthood among those who experienced trauma in early childhood: A qualitative study. Dissertation Abstracts International Section A: Humanities and Social Sciences. 2014;75(4-A(E)).

85. Cross W. A personal history of childhood sexual abuse: Parenting patterns and problems. Clinical Child Psychology and Psychiatry. 2001;6(4):563-74. doi: <http://dx.doi.org/10.1177/1359104501006004010>.

86. Cross SL, Day AG, Byers LG. American Indian grand families: a qualitative study conducted with grandmothers and grandfathers who provide sole care for their grandchildren. J Cross Cult Gerontol. 2010;25(4):371-83. doi: <https://dx.doi.org/10.1007/s10823-010-9127-5>.

87. Crumbley JD. Breaking the intergenerational transmission of physical abuse: An exploratory study. Dissertation Abstracts International. 1990;51(3-A):742.

88. Cummings JA. Transformational change in parenting practices after child interpersonal trauma: A grounded theory examination of parental response. Child Abuse Negl. 2018;76:117-28. doi: 10.1016/j.chiabu.2017.10.005.

89. da Silva RM, de Sousa GS, Vieira L, Caldas JMP, Minayo MCD. Suicidal ideation and attempt of older women in Northeastern Brazil. Rev Bras Enferm. 2018;71:755-62. doi: 10.1590/0034-7167-2017-0413.

90. Dahlen HG, Barclay LM, Homer CSE. Processing the first birth: journeying into 'motherland'. J Clin Nurs. 2010;19(13-14):1977-85. doi: 10.1111/j.1365-2702.2009.03089.x.

91. Dahlen HG, Barclay LM, Homer CSE. The novice birthing: theorising first-time mothers' experiences of birth at home and in hospital in Australia. Midwifery. 2010;26(1):53-63. doi: 10.1016/j.midw.2008.01.012.

92. Dalla RL. When the Bough Breaks ...: Examining Intergenerational Parent-Child Relational Patterns Among Street-Level Sex Workers and Their Parents and Children. Applied Developmental Science. 2003;7(4):216-28. doi: <http://dx.doi.org/10.1207/S1532480XADS0704_1>.

93. Damant D, Lapierre S, Lebosse C, Thibault S, Lessard G, Hamelin-Brabant L, et al. Women's abuse of their children in the context on domestic violence: Reflection from women's accounts. Child & Family Social Work. 2010;15(1):12-21. doi: <http://dx.doi.org/10.1111/j.1365-2206.2009.00632.x>.

94. Danskin KA. The impact of experience on beliefs about motherhood. Dissertation Abstracts International: Section B: The Sciences and Engineering. 2017;78(2-B(E)).

95. Danto D, Walsh R. Mental health perceptions and practices of a Cree community in Northern Ontario: A qualitative study. International Journal of Mental Health and Addiction. 2017;15(4):725-37. doi: <http://dx.doi.org/10.1007/s11469-017-9791-6>.

96. Davies E, Seymour F, Read J. Children's and primary caretakers' perceptions of the sexual abuse investigation process: A New Zealand example. Journal of Child Sexual Abuse: Research, Treatment, & Program Innovations for Victims, Survivors, & Offenders. 2000;9(2):41-56. doi: <http://dx.doi.org/10.1300/J070v09n02_03>.

97. de Oliveira TR, Oliveira Lira Costa RE, Monte NL, de Moura Feitosa Veras JM, Mendes da Rocha Sá MÍ. WOMEN'S PERCEPTION ON OBSTETRIC VIOLENCE. Journal of Nursing UFPE / Revista de Enfermagem UFPE. 2017;11(1):40-6. doi: 10.5205/reuol.9978-88449-6-1101201706.

98. Denham AR. Rethinking historical trauma: narratives of resilience. Transcult Psychiatry. 2008;45(3):391-414. doi: <https://dx.doi.org/10.1177/1363461508094673>.

99. Denov MS. The long-term effects of child sexual abuse by female perpetrators: a qualitative study of male and female victims. J Interpers Violence. 2004;19(10):1137-56.

100. Devieux JG, Jean-Gilles M, Rosenberg R, Beck-Sague C, Attonito JM, Saxena A, et al. Depression, Abuse, Relationship Power and Condom Use by Pregnant and Postpartum Women with Substance Abuse History. Aids Behav. 2016;20(2):292-303. doi: <https://dx.doi.org/10.1007/s10461-015-1176-x>.

101. DeVoe ER, Smith EL. The impact of domestic violence on urban preschool children: battered mothers' perspectives. J Interpers Violence. 2002;17(10):1075-101.

102. DeVoe ER, Smith EL. Don't take my kids: Barriers to service delivery for battered mothers and their young children. Journal of Emotional Abuse. 2003;3(3-4):277-94. doi: <http://dx.doi.org/10.1300/J135v03n03_06>.

103. Dijkstra S. Two mothers abused as children on raising their children. Making a plea for a differentiated approach. Child Abuse Review. 1995;4(4):291-7. doi: <http://dx.doi.org/10.1002/car.2380040408>.

104. Dole DM. Cultural meanings of mothering for African American adolescent mothers: through their own eyes: University of Cincinnati; 2009.

105. Domian EW, Baggett KM, Carta JJ, Mitchell S, Larson E. Factors influencing mothers' abilities to engage in a comprehensive parenting intervention program. Public Health Nurs. 2010;27(5):399-407. doi: <https://dx.doi.org/10.1111/j.1525-1446.2010.00872.x>.

106. Dorr C. Listening to men's stories: Overcoming obstacles to intimacy from childhood. Families in Society. 2001;82(5):509-15. doi: <http://dx.doi.org/10.1606/1044-3894.167>.

107. Dossett EC, Wusirika L, Burt V. No Perfect Choice. JAMA: Journal of the American Medical Association. 2017;318(1):29-30. doi: 10.1001/jama.2017.5523.

108. Dufour MH, Nadeau L. Sexual abuse: a comparison between resilient victims and drug-addicted victims. Violence Vict. 2001;16(6):655-72.

109. Dumbrill GC. Parental experience of child protection intervention: a qualitative study. Child Abuse Negl. 2006;30(1):27-37.

110. Dunlap E, Golub A, Johnson BD. Girls' sexual development in the inner city: from compelled childhood sexual contact to sex-for-things exchanges. J Child Sex Abuse. 2003;12(2):73-96.

111. Dunn B. Growing up with a psychotic mother: a retrospective study. Am J Orthopsychiatry. 1993;63(2):177-89.

112. Efevbera Y, McCoy DC, Wuermli AJ, Betancourt TS. Early Childhood Development Plus Violence Prevention in Low- and Middle-Income Countries: A Qualitative Study. Child Soc. 2017;31(2):98-109. doi: 10.1111/chso.12169.

113. Egeland B, Jacobvitz D, Sroufe LA. Breaking the cycle of abuse. Child Dev. 1988;59(4):1080-8.

114. Elizabeth L. Post-traumatic stress disorder and the violent loss of a child. Awhonn Lifelines. 2006;10(2):178-80.

115. El-Khani A, Ulph F, Peters S, Calam R. Syria: The challenges of parenting in refugee situations of immediate displacement. Intervention: Journal of Mental Health and Psychosocial Support in Conflict Affected Areas. 2016;14(2):99-113. doi: <http://dx.doi.org/10.1097/WTF.0000000000000118>.

116. El-Khani A, Ulph F, Peters S, Calam R. Syria: coping mechanisms utilised by displaced refugee parents caring for their children in pre-resettlement contexts. Intervention. 2017;15(1):34-50. doi: 10.1097/wtf.0000000000000136.

117. Ellsberg M, Pena R, Herrera A, Liljestrand J, Winkvist A. Candies in hell: women's experiences of violence in Nicaragua. Soc Sci Med. 2000;51(11):1595-610.

118. Engnes K, Liden E, Lundgren I. Experiences of being exposed to intimate partner violence during pregnancy. Int J Qual Stud Health Well-being. 2012;7:11. doi: 10.3402/qhw.v7i0.11199.

119. Enriquez LE. Multigenerational punishment: Shared experiences of undocumented immigration status within mixed-status families. Journal of Marriage and Family. 2015;77(4):939-53. doi: <http://dx.doi.org/10.1111/jomf.12196>.

120. Escobar-Chew AR, Carolan M, Burns-Jager K. Connecting trauma and health for mothers in the child welfare system. Journal of Feminist Family Therapy: An International Forum. 2015;27(2):47-71. doi: <http://dx.doi.org/10.1080/08952833.2015.1030331>.

121. Escoto-Lloyd CS. Pregnancy intentions in Latina adolescents: University of California, Los Angeles; 2005.

122. Escriba-Aguir V, Royo-Marques M, Artazcoz L, Romito P, Ruiz-Perez I. Longitudinal study of depression and health status in pregnant women: incidence, course and predictive factors. European Archives of Psychiatry and Clinical Neuroscience. 2013;263(2):143-51. doi: 10.1007/s00406-012-0336-5.

123. Eshed Bar-Sade I. Is there a construct of resilience in fathers who were neglected in childhood and do not maltreat their own children? Dissertation Abstracts International: Section B: The Sciences and Engineering. 2008;69(4-B):2622.

124. Etheridge J, Slade P. "Nothing's actually happened to me.": the experiences of fathers who found childbirth traumatic. Bmc Pregnancy and Childbirth. 2017;17:15. doi: 10.1186/s12884-017-1259-y.

125. Etherington K. Adult male survivors of childhood sexual abuse. Counselling Psychology Quarterly. 1995;8(3):233-41. doi: <http://dx.doi.org/10.1080/09515079508256342>.

126. Etherington K. Creation as transformation: parenting as a turning point in drug users' lives. Counselling & Psychotherapy Research. 2007;7(2):71-8.

127. Evans E. Persistence and success among minority single mothers with substance abuse histories. Dissertation Abstracts International: Section B: The Sciences and Engineering. 2010;71(4-B):2682.

128. Evans J, Rodger S. Mealtimes and bedtimes: windows to family routines and rituals. Journal of Occupational Science. 2008;15(2):98-104.

129. Falletta L, Hamilton K, Fischbein R, Aultman J, Kinney B, Kenne D. Perceptions of child protective services among pregnant or recently pregnant, opioid-using women in substance abuse treatment. Child Abuse Negl. 2018;79:125-35. doi: <http://dx.doi.org/10.1016/j.chiabu.2018.01.026>.

130. Farahzad MM. The impact of motherhood on the mental health & substance use of women who trade sex. Dissertation Abstracts International: Section B: The Sciences and Engineering. 2016;77(4-B(E)).

131. Fenwick J, Gamble J, Creedy D, Barclay L, Buist A, Ryding EL. Women's perceptions of emotional support following childbirth: A qualitative investigation. Midwifery. 2013;29(3):217-24. doi: 10.1016/j.midw.2011.12.008.

132. Few-Demo AL, Arditti JA. Relational vulnerabilities of incarcerated and reentry mothers: therapeutic implications. Int J Offender Ther Comp Criminol. 2014;58(11):1297-320. doi: <https://dx.doi.org/10.1177/0306624X13495378>.

133. Finnbogadóttir H, Dykes A-K. Midwives' awareness and experiences regarding domestic violence among pregnant women in southern Sweden. Midwifery. 2012;28(2):181-9. doi: 10.1016/j.midw.2010.11.010.

134. Finnbogadottir H, Dykes AK, Wann-Hansson C. Struggling to survive for the sake of the unborn baby: a grounded theory model of exposure to intimate partner violence during pregnancy. BMC Pregnancy Childbirth. 2014;14:293. doi: <https://dx.doi.org/10.1186/1471-2393-14-293>.

135. Firestone RW. Parenting groups based on voice therapy. Psychotherapy. 1989;26(4):524-9. doi: <http://dx.doi.org/10.1037/h0085473>.

136. Fisher NK. Mental representations of attachment and caregiving in women sexually abused during childhood: Links to the intergenerational transmission of trauma? Dissertation Abstracts International: Section B: The Sciences and Engineering. 2000;61(2-B):1079.

137. Fitzgerald MM, Shipman KL, Jackson JL, McMahon RJ, Hanley HM. Perceptions of parenting versus parent-child interactions among incest survivors. Child Abuse Negl. 2005;29(6):661-81.

138. Flam AM, Haugstvedt E. Test balloons? Small signs of big events: a qualitative study on circumstances facilitating adults' awareness of children's first signs of sexual abuse. Child Abuse Negl. 2013;37(9):633-42. doi: <https://dx.doi.org/10.1016/j.chiabu.2013.06.007>.

139. Fonfield-Ayinla G. Commentary: A consumer perspective on parenting while homeless. Am J Orthopsychiatry. 2009;79(3):299-300. doi: <http://dx.doi.org/10.1037/a0017239>.

140. Forssen AS. Lifelong significance of disempowering experiences in prenatal and maternity care: interviews with elderly Swedish women. Qual Health Res. 2012;22(11):1535-46. doi: <https://dx.doi.org/10.1177/1049732312449212>.

141. Foster JM. The Fears and Futures of Boy Victims of Sexual Abuse: An Analysis of Narratives. J Child Sex Abuse. 2017;26(6):710-30. doi: 10.1080/10538712.2017.1339223.

142. Fowler C, Lee A. Re-writing motherhood: researching women's experiences of learning to mother for the first time. Aust J Adv Nurs. 2004;22(2):39-44.

143. Fowler C, Dawson A, Rossiter C, Jackson D, Power T, Roche M. When parenting does not "come naturally': providers' perspectives on parenting education for incarcerated mothers and fathers. Stud Contin Educ. 2018;40(1):98-114. doi: 10.1080/0158037x.2017.1396449.

144. Franz TJ. Tell your story: A phenomenological investigation of the experiences of single, Latina mothers living in poverty. Dissertation Abstracts International Section A: Humanities and Social Sciences. 2017;77(9-A(E)).

145. Frisman GH, Eriksson C, Pernehed S, Morelius E. The experience of becoming a grandmother to a premature infant - a balancing act, influenced by ambivalent feelings. J Clin Nurs. 2012;21(21-22):3297-305. doi: 10.1111/j.1365-2702.2012.04204.x.

146. Frost-Pineda K. Addiction and pregnancy intentions: Understanding the why behind the what. Dissertation Abstracts International Section A: Humanities and Social Sciences. 2009;69(7-A):2766.

147. Gaensbauer TJ, Jordan L. Psychoanalytic perspectives on early trauma: interviews with thirty analysts who treated an adult victim of a circumscribed trauma in early childhood. J Am Psychoanal Assoc. 2009;57(4):947-77. doi: <https://dx.doi.org/10.1177/0003065109342589>.

148. Galera SA, Bernal Roldan MC, O'Brien B. Women living in a drug (and violence) context--the maternal role. Rev Lat Am Enfermagem. 2005;13 Spec No:1142-7.

149. Ghaffar W, Manby M, Race T. Exploring the experiences of parents and carers whose children have been subject to child protection plans. British Journal of Social Work. 2012;42(5):887-905. doi: <http://dx.doi.org/10.1093/bjsw/bcr132>.

150. Gil T. Mothering among women with histories of child sexual abuse. Dissertation Abstracts International Section A: Humanities and Social Sciences. 2010;71(3-A):1090.

151. Gilgun JF, Anderson G. Mothers' perspectives on signs of child sexual abuse in their families. Families in Society. 2013;94(4):259-67. doi: <http://dx.doi.org/10.1606/1044-3894.4324>.

152. Gojman-de-Millan S, Millan S. Mental collapse as "disorganized attachment": A dynamic understanding for clinicians. International Forum of Psychoanalysis. 2017. doi: <http://dx.doi.org/10.1080/0803706X.2017.1304655>.

153. Gold JI, Taft CT, Keehn MG, King DW, King LA, Samper RE. PTSD symptom severity and family adjustment among female Vietnam veterans. Military Psychology. 2007;19(2):71-81. doi: <http://dx.doi.org/10.1080/08995600701323368>.

154. Goldberg ME. Substance-abusing women: false stereotypes and real needs. Soc Work. 1995;40(6):789-98.

155. Goldberg LR, Heiss CJ, White L, Kaf WA, Becker A, Schindler JB, et al. Methamphetamine exposure, iron deficiency, and implications for cognitive-communicative function: A case study. Communication Disorders Quarterly. 2010;31(3):183-92. doi: <http://dx.doi.org/10.1177/1525740109340437>.

156. Goldblatt H. Caring for abused women: impact on nurses' professional and personal life experiences. J Adv Nurs. 2009;65(8):1645-54. doi: <https://dx.doi.org/10.1111/j.1365-2648.2009.05019.x>.

157. Golden SD, Perreira KM, Durrance CP. Troubled times, troubled relationships: how economic resources, gender beliefs, and neighborhood disadvantage influence intimate partner violence. J Interpers Violence. 2013;28(10):2134-55. doi: <https://dx.doi.org/10.1177/0886260512471083>.

158. Golding KS. Connection Before Correction: Supporting Parents to Meet the Challenges of Parenting Children who have been Traumatised within their Early Parenting Environments. Children Australia. 2015;40(2):152-9. doi: 10.1017/cha.2015.9.

159. Goldsmith J, Cowen H. The inheritance of loss. Journal of Child Psychotherapy. 2011;37(2):179-93. doi: <http://dx.doi.org/10.1080/0075417X.2011.581473>.

160. Goldson E, Cadol RV, Fitch MJ, Umlauf HJ, Jr. Nonaccidental trauma and failure to thrive. Am J Dis Child. 1976;130(5):490-2.

161. Golfenshtein N, Deatrick JA, Lisanti AJ, Medoff-Cooper B. Coping with the Stress in the Cardiac Intensive Care Unit: Can Mindfulness Be the Answer? J Pediatr Nurs. 2017;37:117-26. doi: 10.1016/j.pedn.2017.08.021.

162. Golub Y, Canneva F, Funke R, Frey S, Distler J, von Horsten S, et al. Effects of In utero environment and maternal behavior on neuroendocrine and behavioral alterations in a mouse model of prenatal trauma. Dev Neurobiol. 2016;76(11):1254-65. doi: 10.1002/dneu.22387.

163. Gomez AM. Healing the caregiving system: Working with parents within a comprehensive EMDR treatment. Journal of EMDR Practice and Research. 2012;6(3):136-44. doi: <http://dx.doi.org/10.1891/1933-3196.6.3.136>.

164. Gondwe KW, Holditch-Davis D. Posttraumatic stress symptoms in mothers of preterm infants. International Journal of Africa Nursing Sciences. 2015;3:8-17. doi: <http://dx.doi.org/10.1016/j.ijans.2015.05.002>.

165. Gondwe KW, White-Traut R, Brandon D, Pan W, Holditch-Davis D. The role of sociodemographic factors in maternal psychological distress and mother-preterm infant interactions. Res Nurs Health. 2017;40(6):528-40. doi: <https://dx.doi.org/10.1002/nur.21816>.

166. Gonzalez NM, Campbell M. Cocaine babies: Does prenatal exposure to cocaine affect development? J Am Acad Child Adolesc Psychiatry. 1994;33(1):16-9. doi: <http://dx.doi.org/10.1097/00004583-199401000-00003>.

167. Gonzalez A, Jenkins JM, Steiner M, Fleming AS. Maternal early life experiences and parenting: the mediating role of cortisol and executive function. J Am Acad Child Adolesc Psychiatry. 2012;51(7):673-82. doi: <https://dx.doi.org/10.1016/j.jaac.2012.04.003>.

168. Goodkind JR, Hess JM, Gorman B, Parker DP. "We're still in a struggle": Dine resilience, survival, historical trauma, and healing. Qual Health Res. 2012;22(8):1019-36. doi: <https://dx.doi.org/10.1177/1049732312450324>.

169. Goodman LA. The prevalence of abuse among homeless and housed poor mothers: a comparison study. Am J Orthopsychiatry. 1991;61(4):489-500.

170. Goodman DJ, Milliken CU, Theiler RN, Nordstrom BR, Akerman SC. A Multidisciplinary Approach to the Treatment of Co-occurring Opioid Use Disorder and Posttraumatic Stress Disorder in Pregnancy: A Case Report. J Dual Diagn. 2015;11(3-4):248-57. doi: <https://dx.doi.org/10.1080/15504263.2015.1104484>.

171. Goodman ML, Martinez K, Keiser PH, Gitari S, Seidel SE. Why do kenyan children live on the streets? Evidence from a cross-section of semi-rural maternal caregivers. Child Abuse Negl. 2017;63:51-60. doi: <http://dx.doi.org/10.1016/j.chiabu.2016.10.018>.

172. Goodyear RK, Newcomb MD, Locke TF. Pregnant Latina teenagers: Psychosocial and developmental determinants of how they select and perceive the men who father their children. Journal of Counseling Psychology. 2002;49(2):187-201. doi: <http://dx.doi.org/10.1037/0022-0167.49.2.187>.

173. Goosen S, Stronks K, Kunst AE. Frequent relocations between asylum-seeker centres are associated with mental distress in asylum-seeking children: a longitudinal medical record study. Int J Epidemiol. 2014;43(1):94-104. doi: <https://dx.doi.org/10.1093/ije/dyt233>.

174. Gordon D, Burge D, Hammen C, Adrian C, Jaenicke C, Hiroto D. Observations of interactions of depressed women with their children. Am J Psychiatry. 1989;146(1):50-5.

175. Gosh Ippen C, Norona CR, Lieberman AF. Clinical considerations for conducting Child-Parent Psychotherapy with young children with developmental disabilities who have experienced trauma. Pragmatic Case Studies in Psychotherapy. 2014;10(3):196-211.

176. Goutaudier N, Lopez A, Sejourne N, Denis A, Chabrol H. Premature birth: Subjective and psychological experiences in the first weeks following childbirth, a mixed-methods study. Journal of Reproductive and Infant Psychology. 2011;29(4):364-73. doi: <http://dx.doi.org/10.1080/02646838.2011.623227>.

177. Goutaudier N, Sejourne N, Rousset C, Lami C, Chabrol H. Negative emotions, childbirth pain, perinatal dissociation and self-efficacy as predictors of postpartum posttraumatic stress symptoms. Journal of Reproductive and Infant Psychology. 2012;30(4):352-62. doi: <http://dx.doi.org/10.1080/02646838.2012.738415>.

178. Grabow AP, Khurana A, Natsuaki MN, Neiderhiser JM, Harold GT, Shaw DS, et al. Using an adoption-biological family design to examine associations between maternal trauma, maternal depressive symptoms, and child internalizing and externalizing behaviors. Development and Psychopathology. 2017;29(5):1707-20. doi: 10.1017/s0954579417001341.

179. Grafwallner PEG. What I Learned. Exceptional Parent. 2016;46(9):28-30.

180. Graham-Bermann SA, Levendosky AA. The social functioning of preschool-age children whose mothers are emotionally and physically abused. Journal of Emotional Abuse. 1997;1(1):59-84. doi: <http://dx.doi.org/10.1300/J135v01n01_04>.

181. Graham-Bermann SA, Seng J. Violence exposure and traumatic stress symptoms as additional predictors of health problems in high-risk children. J Pediatr. 2005;146(3):349-54.

182. Graham-Bermann SA, DeVoe ER, Mattis JS, Lynch S, Thomas SA. Ecological predictors of traumatic stress symptoms in caucasian and ethnic minority children exposed to intimate partner violence. Violence Against Women. 2006;12(7):663-92.

183. Graham-Bermann SA, Howell K, Habarth J, Krishnan S, Loree A, Bermann EA. Toward assessing traumatic events and stress symptoms in preschool children from low-income families. Am J Orthopsychiatry. 2008;78(2):220-8. doi: <https://dx.doi.org/10.1037/a0013977>.

184. Graham-Bermann SA, Gruber G, Howell KH, Girz L. Factors discriminating among profiles of resilience and psychopathology in children exposed to intimate partner violence (IPV). Child Abuse Negl. 2009;33(9):648-60. doi: <https://dx.doi.org/10.1016/j.chiabu.2009.01.002>.

185. Graham-Bermann SA, Howell KH, Miller LE, Kwek J, Lilly MM. Traumatic events and maternal education as predictors of verbal ability for preschool children exposed to intimate partner violence (IPV). Journal of Family Violence. 2010;25(4):383-92. doi: 10.1007/s10896-009-9299-3.

186. Graham-Bermann SA, Howell KH, Lilly M, Devoe E. Mediators and moderators of change in adjustment following intervention for children exposed to intimate partner violence. J Interpers Violence. 2011;26(9):1815-33. doi: <https://dx.doi.org/10.1177/0886260510372931>.

187. Graham-Bermann SA, Kulkarni MR, Kanukollu SN. Is disclosure therapeutic for children following exposure to traumatic violence? J Interpers Violence. 2011;26(5):1056-76. doi: <https://dx.doi.org/10.1177/0886260510365855>.

188. Graham-Bermann SA, Miller LE. Intervention to reduce traumatic stress following intimate partner violence: an efficacy trial of the Moms' Empowerment Program (MEP). Psychodyn. 2013;41(2):329-49. doi: <https://dx.doi.org/10.1521/pdps.2013.41.2.329>.

189. Granek L, Rosenberg-Yunger ZR, Dix D, Klaassen RJ, Sung L, Cairney J, et al. Caregiving, single parents and cumulative stresses when caring for a child with cancer. Child Care Health Dev. 2014;40(2):184-94. doi: <https://dx.doi.org/10.1111/cch.12008>.

190. Granqvist P, Forslund T, Fransson M, Springer L, Lindberg L. Mothers with intellectual disability, their experiences of maltreatment, and their children's attachment representations: a small-group matched comparison study. Attach Hum Dev. 2014;16(5):417-36. doi: <https://dx.doi.org/10.1080/14616734.2014.926946>.

191. Grant T, Graham J, Ernst CC, Peavy K, Brown NN. Improving pregnancy outcomes among high-risk mothers who abuse alcohol and drugs: Factors associated with subsequent exposed births. Children and Youth Services Review. 2014;46:11-8. doi: <http://dx.doi.org/10.1016/j.childyouth.2014.07.014>.

192. Grasso DJ, Henry D, Kestler J, Nieto R, Wakschlag LS, Briggs-Gowan MJ. Harsh Parenting As a Potential Mediator of the Association Between Intimate Partner Violence and Child Disruptive Behavior in Families With Young Children. J Interpers Violence. 2016;31(11):2102-26. doi: <https://dx.doi.org/10.1177/0886260515572472>.

193. Greaves L, Chabot C, Jategaonkar N, Poole N, McCullough L. Substance use among women in shelters for abused women and children. Programming opportunities. Can J Public Health. 2006;97(5):388-92.

194. Green AH, Coupe P, Fernandez R, Stevens B. Incest revisited: delayed post-traumatic stress disorder in mothers following the sexual abuse of their children. Child Abuse Negl. 1995;19(10):1275-82.

195. Greene CA, Chan G, McCarthy KJ, Wakschlag LS, Briggs-Gowan MJ. Psychological and physical intimate partner violence and young children's mental health: The role of maternal posttraumatic stress symptoms and parenting behaviors. Child Abuse Negl. 2018;77:168-79. doi: 10.1016/j.chiabu.2018.01.012.

196. Greif GL, Drechsler M. Common issues for parents in a methadone maintenance group. J Subst Abuse Treat. 1993;10(4):339-43. doi: <http://dx.doi.org/10.1016/0740-5472%2893%2990018-W>.

197. Grekin R, Brock RL, O'Hara MW. The effects of trauma on perinatal depression: Examining trajectories of depression from pregnancy through 24 months postpartum in an at-risk population. J Affect Disord. 2017;218:269-76. doi: <https://dx.doi.org/10.1016/j.jad.2017.04.051>.

198. Guardino CM, Schetter CD. Coping during pregnancy: a systematic review and recommendations. Health Psychol Rev. 2014;8(1):70-94. doi: 10.1080/17437199.2012.752659.

199. Haggett BA. The lived experience of motherhood: A personal exploration. Dissertation Abstracts International: Section B: The Sciences and Engineering. 2013;74(6-B(E)).

200. Haight WL, Shim WS, Linn LM, Swinford L. Mothers' strategies for protecting children from batterers: the perspectives of battered women involved in child protective services. Child Welfare. 2007;86(4):41-62.

201. Haight WL, Carter-Black JD, Sheridan K. Mothers' experience of methamphetamine addiction: a case-based analysis of rural, midwestern women. Children and Youth Services Review. 2009;31(1):71-7.

202. Hall S. "It's going to stop in this generation": Women with a history of child abuse resolving to raise their children without abuse. Harvard Educational Review. 2011;81(1):24-49. doi: <http://dx.doi.org/10.17763/haer.81.1.m8202r3804uvmv68>.

203. Hall JM. Women survivors of childhood abuse: the impact of traumatic stress on education and work. Issues Ment Health Nurs. 2000;21(5):443-71.

204. Halperin O, Sarid O, Cwikel J. The influence of childbirth experiences on women's postpartum traumatic stress symptoms: A comparison between Israeli Jewish and Arab women. Midwifery. 2015;31(6):625-32. doi: <https://dx.doi.org/10.1016/j.midw.2015.02.011>.

205. Halsey M, Deegan S. Father and son: Two generations through prison. Punishm Soc. 2012;14(3):338-67. doi: 10.1177/1462474512442315.

206. Halvorsen L, Nerum H, Oian P, Sorlie T. Giving birth with rape in one's past: a qualitative study. Birth. 2013;40(3):182-91. doi: <https://dx.doi.org/10.1111/birt.12054>.

207. Hanlon C, Whitley R, Wondimagegn D, Alem A, Prince M. Between life and death: exploring the sociocultural context of antenatal mental distress in rural Ethiopia. Archives of Womens Mental Health. 2010;13(5):385-93. doi: 10.1007/s00737-010-0149-3.

208. Harner HM, Riley S. The impact of incarceration on women's mental health: responses from women in a maximum-security prison. Qual Health Res. 2013;23(1):26-42. doi: <https://dx.doi.org/10.1177/1049732312461452>.

209. Harris-McKoy DE, Woods SB, Brantley CW, Farineau HM. EXPERIENCES OF FAMILY THERAPISTS WORKING WITH FAMILIES IN A TRANSITIONAL HOMELESS COMMUNITY. Journal of Marital and Family Therapy. 2015;41(2):192-204. doi: 10.1111/jmft.12062.

210. Harvey ME, Pattison HM. Being there: a qualitative interview study with fathers present during the resuscitation of their baby at delivery. Arch Dis Child Fetal Neonatal Ed. 2012;97(6):F439-43. doi: <https://dx.doi.org/10.1136/archdischild-2011-301482>.

211. Hayes MO. The lived experience of mothering after prison: Boston College; 2007.

212. Henderson AD. Abused women's perceptions of their children's experiences. Canada's Mental Health. 1993;41(1):7-11.

213. Henriksen L, Grimsrud E, Schei B, Lukasse M, Bidens Study G. Factors related to a negative birth experience - A mixed methods study. Midwifery. 2017;51:33-9. doi: <https://dx.doi.org/10.1016/j.midw.2017.05.004>.

214. Herland MD, Helgeland IM. Negotiating motherhood: Women with troubled upbringings and their self-conceptions as mothers. Child & Family Social Work. 2017;22(1):47-56. doi: <http://dx.doi.org/10.1111/cfs.12193>.

215. Herland MD, Hauge M-I, Helgeland IM. Balancing fatherhood: experiences of fatherhood among men with a difficult past. Qualitative Social Work. 2015;14(2):242-58.

216. Heward-Belle S. Exploiting the 'good mother' as a tactic of coercive control: domestically violent men's assaults on women as mothers. Affilia. 2017;32(3):374-89.

217. Hill LE. Haudenosaunee grandmothers raising their grandchildren: Bears of our families and community. Dissertation Abstracts International Section A: Humanities and Social Sciences. 2013;73(10-A(E)).

218. Hilton TP, Trella DL. You just gotta do It, 'cause those are your kids: Survival parenting and rural homelessness. World Medical and Health Policy. 2014;6(4):446-82. doi: <http://dx.doi.org/10.1002/wmh3.115>.

219. Hinton L, Locock L, Knight M. Partner experiences of "near-miss" events in pregnancy and childbirth in the UK: a qualitative study.[Erratum appears in PLoS One. 2014;9(9):e108803]. PLoS ONE. 2014;9(4):e91735. doi: <https://dx.doi.org/10.1371/journal.pone.0091735>.

220. Hinton L, Locock L, Knight M. Support for mothers and their families after life-threatening illness in pregnancy and childbirth: a qualitative study in primary care. Br J Gen Pract. 2015;65(638):e563-9. doi: <https://dx.doi.org/10.3399/bjgp15X686461>.

221. Hodgdon M. Impact of voices curriculum on adolescent mothers. Dissertation Abstracts International: Section B: The Sciences and Engineering. 2018;78(10-B(E)).

222. Hogg S, Coster D, Brookes H. Baby Steps : perspectives of parents from a minority ethnic background. London: NSPCC, 2015.

223. Holka-Pokorska J, Jarema M, Stefanowicz A, Pirog-Balcerzak A, Wichniak A. Elective cesarean section on psychiatric indications - the phenomenon analysis, report of two cases and psychiatric clinical recommendations. Psychiatr Pol. 2016;50(2):357-73. doi: <https://dx.doi.org/10.12740/PP/43486>.

224. Holliday CN, Miller E, Decker MR, Burke JG, Documet PI, Borrero SB, et al. Racial Differences in Pregnancy Intention, Reproductive Coercion, and Partner Violence among Family Planning Clients: A Qualitative Exploration. Women's Health Issues. 2018;28(3):205-11. doi: 10.1016/j.whi.2018.02.003.

225. Holt S. Domestic abuse and child contact: Positioning children in the decision-making process. Child Care in Practice. 2011;17(4):327-46. doi: <http://dx.doi.org/10.1080/13575279.2011.596817>.

226. Holt S. Post-separation fathering and domestic abuse: Challenges and contradictions. Child Abuse Review. 2015;24(3):210-22. doi: <http://dx.doi.org/10.1002/car.2264>.

227. Holt S. Domestic Violence and the Paradox of Post-Separation Mothering. British Journal of Social Work. 2017;47(7):2049-67. doi: 10.1093/bjsw/bcw162.

228. Hooper C-A, Koprowska J. The vulnerabilities of children whose parents have been sexually abused in childhood: Towards a new framework. British Journal of Social Work. 2004;34(2):165-80. doi: <http://dx.doi.org/10.1093/bjsw/bch020>.

229. Hudson L, Beilke S, Many M. "If You Brave Enough to Live It, the Least I Can Do Is Listen". Zero to Three. 2016;36(5):4-11.

230. Hughes MJ, Rasmussen LA. The utility of motivational interviewing in domestic violence shelters: A qualitative exploration. Journal of Aggression, Maltreatment and Trauma. 2010;19(3):300-22. doi: <http://dx.doi.org/10.1080/10926771003705213>.

231. Hughes J, Chau S, Poff DC. “They're not my favourite people”: what mothers who have experienced intimate partner violence say about involvement in the child protection system. Children and Youth Services Review. 2011;33(7):1084-9.

232. Hurd RC. Sibling support systems in childhood after a parent dies. Omega: Journal of Death and Dying. 2002;45(4):299-320. doi: <http://dx.doi.org/10.2190/B92T-LPQR-1RP9-9562>.

233. Iles J, Pote H. Postnatal posttraumatic stress: A grounded theory model of first-time mothers' experiences. Journal of Reproductive and Infant Psychology. 2015;33(3):238-55. doi: <http://dx.doi.org/10.1080/02646838.2015.1030732>.

234. Insetta ER, Akers AY, Miller E, Yonas MA, Burke JG, Hintz L, et al. Intimate partner violence victims as mothers: their messages and strategies for communicating with children to break the cycle of violence. J Interpers Violence. 2015;30(4):703-24. doi: <https://dx.doi.org/10.1177/0886260514535264>.

235. Izaguirre A, Calvete E. Children who are exposed to intimate partner violence: Interviewing mothers to understand its impact on children. Child Abuse Negl. 2015;48:58-67. doi: <https://dx.doi.org/10.1016/j.chiabu.2015.05.002>.

236. Jack S. Mothers with post-traumatic stress disorder after traumatic childbirth struggled to survive and experienced nightmares, flashbacks, anger, anxiety, depression, and isolation. Evidence Based Nursing. 2005;8(2):59-.

237. Jack SM, Ford-Gilboe M, Davidov D, MacMillan HL, Team NIR. Identification and assessment of intimate partner violence in nurse home visitation. J Clin Nurs. 2017;26(15-16):2215-28. doi: <https://dx.doi.org/10.1111/jocn.13392>.

238. Jackson S, Kelly L, Leslie B. Parental participation in statutory child protection intervention in Scotland. British Journal of Social Work. 2017;47(5):1445-63.

239. Jacobs L, Jacobs J. 'Bad' mothers have alcohol use disorder: Moral panic or brief intervention? Gender & Behaviour. 2014;12(1):5971-9.

240. Jammeh A, Sundby J, Vangen S. Barriers to emergency obstetric care services in perinatal deaths in rural Gambia: A qualitative in-depth interview study. ISRN Obstetrics and Gynecology. 2011:981096. doi: <http://dx.doi.org/10.5402/2011/981096>.

241. Jewkes R, Penn-Kekana L, Rose-Junius H. ''If they rape me, I can't blame them": reflections on gender in the social context of child rape in South Africa and Namibia. Soc Sci Med. 2005;61(8):1809-20.

242. Johnson HD, Young DS. Addiction, abuse, and family relationships: Childhood experiences of five incarcerated African American women. J Ethn Subst Abuse. 2002;1(4):29-47. doi: <http://dx.doi.org/10.1300/J233v01n04_02>.

243. Johnston DA. Releasing the flood: a qualitative case study of one high-risk father's journey through the labor unit and neonatal intensive care unit. J Perinat Neonatal Nurs. 2014;28(4):319-31. doi: <https://dx.doi.org/10.1097/JPN.0000000000000064>.

244. Jones A, Vetere A. 'You just deal with it. You have to when you've got a child': A narrative analysis of mothers' accounts of how they coped, both during an abusive relationship and after leaving. Clin. 2017;22(1):74-89. doi: <https://dx.doi.org/10.1177/1359104515624131>.

245. Jones N. The experiences of African American women who were psychologically abused by their parents during childhood. Dissertation Abstracts International Section A: Humanities and Social Sciences. 2017;78(4-A(E)).

246. Kadish Y. Five women's recollections and reflections on being raised by a mother with psychosis. South African Journal of Psychology. 2015;45(4):480-94. doi: 10.1177/0081246315581565.

247. Kallan JC. "It's like a different kind of parenting": Constructions of good and bad parenting in neonatal intensive care. Dissertation Abstracts International Section A: Humanities and Social Sciences. 2014;75(1-A(E)).

248. Kamal L, Strand J, Jutengren G, Tidefors I. Perceptions and experiences of an attachment-based intervention for parents troubled by intimate partner violence. Clinical Social Work Journal. 2017;45(4):311–9.

249. Kamite Y. Prejudice and health anxiety about radiation exposure from second-generation atomic bomb survivors: Results from a qualitative interview study. Front Psychol. 2017;8:1462. doi: <http://dx.doi.org/10.3389/fpsyg.2017.01462>.

250. Kanku T. Attitudes, perceptions and understanding amongst teenagers regarding teenage pregnancy, sexuality and contraception in Taung. South African Family Practice. 2010;52(6):563-72.

251. Kantrowitz-Gordon I, Altman MR, Vandermause R. Prolonged Distress of Parents After Early Preterm Birth. Jognn. 2016;45(2):196-209. doi: 10.1016/j.jogn.2015.12.004.

252. Karver TS, Sorhaindo A, Wilson KS, Contreras X. Exploring intergenerational changes in perceptions of gender roles and sexuality among Indigenous women in Oaxaca. Cult Health Sex. 2016;18(8):845-59. doi: 10.1080/13691058.2016.1144790.

253. Kassam-Adams N, Rzucidlo S, Campbell M, Good G, Bonifacio E, Slouf K, et al. Nurses' Views and Current Practice of Trauma-Informed Pediatric Nursing Care. J Pediatr Nurs. 2015;30(3):478-84. doi: 10.1016/j.pedn.2014.11.008.

254. Katsumaru M, Esaki T, Ikeda M, Sakai M, Watanabe H. Reattachment therapy for adolescent anorexia nervosa considering about becoming good enough mother. Neuropsychiatrie de l'Enfance et de l'Adolescence. 2012;1):S156. doi: <http://dx.doi.org/10.1016/j.neurenf.2012.04.188>.

255. Katz JR. A collective case study of Native American nurses from the Plateau tribes: Gonzaga University; 2003.

256. Katz LS, Huffman C, Cojucar G. In Her Own Words: Semi-structured Interviews of Women Veterans Who Experienced Military Sexual Assault. Journal of Contemporary Psychotherapy. 2017;47(3):181-9. doi: <http://dx.doi.org/10.1007/s10879-016-9349-0>.

257. Katz E. Strengthening mother-child relationships as part of domestic violence recovery. Edinburgh: Centre for Research on Families and Relationships, 2014.

258. Katz E. Recovery-promoters:Ways in which children and mothers support one another's recoveries from domestic violence. British Journal of Social Work. 2015;45(Supp 1):153-69. doi: <http://dx.doi.org/10.1093/bjsw/bcv091>.

259. Katz E. Beyond the Physical Incident Model: How Children Living with Domestic Violence are Harmed By and Resist Regimes of Coercive Control. Child Abuse Review. 2016;25(1):46-59. doi: 10.1002/car.2422.

260. Kaye DK, Ekstrom AM, Johansson A, Bantebya G, Mirembe FM. Escaping the triple trap: coping strategies of pregnant adolescent survivors of domestic violence in Mulago hospital, Uganda. Scand J Public Health. 2007;35(2):180-6.

261. Kaye DK, Kakaire O, Nakimuli A, Mbalinda SN, Osinde MO, Kakande N. Survivors' understanding of vulnerability and resilience to maternal near-miss obstetric events in Uganda. Int J Gynaecol Obstet. 2014;127(3):265-8. doi: <https://dx.doi.org/10.1016/j.ijgo.2014.05.019>.

262. Kaye DK, Kakaire O, Nakimuli A, Osinde MO, Mbalinda SN, Kakande N. Lived experiences of women who developed uterine rupture following severe obstructed labor in Mulago hospital, Uganda. Reprod Health. 2014;11:31. doi: <https://dx.doi.org/10.1186/1742-4755-11-31>.

263. Kearney JA, Cushing E. A multi-modal pilot intervention with violence-exposed mothers in a child psychiatric trauma-focused treatment program. Issues Ment Health Nurs. 2012;33(8):544-52. doi: <https://dx.doi.org/10.3109/01612840.2012.688254>.

264. Keim MC, Fortney CA, Shultz EL, Winning A, Gerhardt CA, Baughcum A. Parent Distress and the Decision to Have Another Child After an Infant's Death in the NICU. J Obstet Gynecol Neonatal Nurs. 2017;46(3):446-55. doi: <https://dx.doi.org/10.1016/j.jogn.2017.01.009>.

265. Keiski P, Flinck A, Kaunonen M, Paavilainen E. Women’s experiences of perpetrating family violence: a qualitative study. Journal of Research in Nursing. 2016;21(5/6):417-29. doi: 10.1177/1744987116653439.

266. Kelleher L, Cleary M, Jackson D. Compulsory participation in a child protection and family enhancement program: mothers' experiences. Contemp Nurse. 2012;41(1):101-10. doi: <https://dx.doi.org/10.5172/conu.2012.41.1.101>.

267. Kelly Cardona R. Parenting women in gender-responsive substance use disorder treatment: A phenomenological inquiry. Dissertation Abstracts International Section A: Humanities and Social Sciences. 2016;76(7-A(E)).

268. Kenny KS, Barrington C, Green SL. "I felt for a long time like everything beautiful in me had been taken out": Women's suffering, remembering, and survival following the loss of child custody. Int J Drug Policy. 2015;26(11):1158-66. doi: <https://dx.doi.org/10.1016/j.drugpo.2015.05.024>.

269. Kidner PJ. A tale that can be told: Mother-child narratives of domestic abuse. Dissertation Abstracts International Section C: Worldwide. 2018;75(4-C).

270. Kidner MC, Flanders-Stepans MB. A model for the HELLP syndrome: the maternal experience. JOGNN: Journal of Obstetric, Gynecologic & Neonatal Nursing. 2004;33(1):44-53. doi: 10.1177/0884217503261131.

271. Killion CM. Poverty and procreation among women - An anthropologic study with implications for health care providers. J Nurse Midwifery. 1998;43(4):273-9. doi: 10.1016/s0091-2182(98)00013-5.

272. Kilroy SJ, Egan J, Maliszewska A, Sarma KM. "Systemic trauma": the impact on parents whose children have experienced sexual abuse. J Child Sex Abuse. 2014;23(5):481-503. doi: <https://dx.doi.org/10.1080/10538712.2014.920458>.

273. Kirkman M, Apicella C, Graham J, Hickey M, Hopper JL, Keogh L, et al. Meanings of abortion in context: accounts of abortion in the lives of women diagnosed with breast cancer. BMC Womens Health. 2017;17(1):26. doi: <https://dx.doi.org/10.1186/s12905-017-0383-1>.

274. Kitzinger C, Kitzinger S. Birth trauma: talking with women and the value of conversation analysis. British Journal of Midwifery. 2007;15(5):256-60.

275. Kitzinger S. Rediscovering the social model of childbirth. Birth. 2012;39(4):301-4. doi: <https://dx.doi.org/10.1111/birt.12005>.

276. Koren-Karie N, Oppenheim D, Getzler-Yosef R. Mothers Who Were Severely Abused During Childhood and Their Children Talk About Emotions: Co-Construction of Narratives in Light of Maternal Trauma. Infant Ment Health J. 2004;25(4):300-17. doi: <http://dx.doi.org/10.1002/imhj.20007>.

277. Koren-Karie N, Oppenheim D, Getzler-Yosef R. Shaping children's internal working models through mother-child dialogues: the importance of resolving past maternal trauma. Attach Hum Dev. 2008;10(4):465-83. doi: <https://dx.doi.org/10.1080/14616730802461482>.

278. Kovalesky A. Factors affecting mother-child visiting identified by women with histories of substance abuse and child custody loss. Child Welfare. 2001;80(6):749-68.

279. Kruger L-M, van Straaten K, Taylor L, Lourens M, Dukas C. The melancholy of murderous mothers: Depression and the medicalization of women's anger. Feminism & Psychology. 2014;24(4):461-78. doi: <http://dx.doi.org/10.1177/0959353514539653>.

280. Kruk E. Collateral damage: The lived experiences of divorced mothers without custody. Journal of Divorce & Remarriage. 2010;51(8):526-43. doi: <http://dx.doi.org/10.1080/10502556.2010.504103>.

281. Kulkarni S. Interpersonal violence at the crossroads between adolescence and adulthood - Learning about partner violence from young mothers. Violence Against Women. 2006;12(2):187-207. doi: 10.1177/1077801205280933.

282. Kyskan C. Evolution of the fragile fighter: A qualitative exploration of seven mothers in a substance abuse treatment program. Dissertation Abstracts International: Section B: The Sciences and Engineering. 2007;67(12-B):7380.

283. Lainsbury D. Child is father to the man: a survivor of childhood sexual abuse speaks out. Midwifery Today. 2009;(90):16-65.

284. Laird-Bloom AD. A phenomenological analysis of the lived experience of male partners of female childhood sexual abuse survivors. Dissertation Abstracts International: Section B: The Sciences and Engineering. 2002;62(9-B):4275.

285. Lake KL. Long-term effects on adult adjustment in intimate relationships that differ by gender: A qualitative investigation of adults who have witnessed domestic violence as children. Dissertation Abstracts International: Section B: The Sciences and Engineering. 2002;63(5-B):2590.

286. Lake RP. Recovery from childhood abuse: A cultural context. Dissertation Abstracts International: Section B: The Sciences and Engineering. 2004;65(3-B):1551.

287. LaMancuso K, Goldman RE, Nothnagle M. "Can I Ask That?": Perspectives on Perinatal Care After Resettlement Among Karen Refugee Women, Medical Providers, and Community-Based Doulas. J Immigr Minor Health. 2016;18(2):428-35. doi: <https://dx.doi.org/10.1007/s10903-015-0172-6>.

288. Lamb LC. A phenomenological examination into the lived experiences of maternal dissatisfaction. Dissertation Abstracts International Section A: Humanities and Social Sciences. 2010;70(10-A):4069.

289. Lanctôt N, Turcotte M. The 'good mother' struggles: obstacles to the attainment of motherhood ideals among adult women formerly placed in residential care. Child and Family Social Work. 2017;23(1):80-7.

290. Lapierre S. Striving to be 'good' mothers: abused women's experiences of mothering. Child Abuse Review. 2010;19(5):342-57.

291. Lear T. Women's perceptions of the birthing experience: An ever-changing phenomenon. Journal of Prenatal & Perinatal Psychology & Health. 2006;21(2):203-10.

292. Leeners B, Stiller R, Block E, Gorres G, Imthurn B, Rath W. Effect of childhood sexual abuse on gynecologic care as an adult. Psychosomatics. 2007;48(5):385-93. doi: 10.1176/appi.psy.48.5.385.

293. Leeners B, Gorres G, Block E, Hengartner MP. Birth experiences in adult women with a history of childhood sexual abuse. J Psychosom Res. 2016;83:27-32. doi: <https://dx.doi.org/10.1016/j.jpsychores.2016.02.006>.

294. Leon K, Jacobvitz DB, Hazen NL. Maternal Resolution of Loss and Abuse: Associations with Adjustment to the Transition to Parenthood. Infant Ment Health J. 2004;25(2):130-48. doi: <http://dx.doi.org/10.1002/imhj.10091>.

295. Lewinsohn R, Crankshaw T, Tomlinson M, Gibbs A, Butler L, Smit J. “This baby came up and then he said, “I give up!”: The interplay between unintended pregnancy, sexual partnership dynamics and social support and the impact on women's well-being in KwaZulu-Natal, South Africa. Midwifery. 2018;62:29-35. doi: 10.1016/j.midw.2018.03.001.

296. Limoges C. The lived family experiences of custodial grandparents raising abused or neglected grandchildren. Dissertation Abstracts International: Section B: The Sciences and Engineering. 2015;76(3-B(E)).

297. Lingen-Stallard A, Furber C, Lavender T. Testing HIV positive in pregnancy: A phenomenological study of women's experiences. Midwifery. 2016;35:31-8. doi: 10.1016/j.midw.2016.02.008.

298. Linton KF, Rueda HA. Experiences with pregnancy of adolescents with disabilities from the perspectives of the school social workers who serve them. Health Soc Work. 2014;39(2):92-100.

299. LoGiudice JA, Beck CT. The Lived Experience of Childbearing From Survivors of Sexual Abuse: "It Was the Best of Times, It Was the Worst of Times". J Midwifery Womens Health. 2016;61(4):474-81. doi: <https://dx.doi.org/10.1111/jmwh.12421>.

300. LoGiudice JA. Birth as Restorative. J Am Psychiatr Nurses Assoc. 2018;24(2):153-4. doi: 10.1177/1078390317734632.

301. LoVerso T. Experiences of motherhood that contribute to the recovery processes of adult survivors of child sexual abuse. Dissertation Abstracts International Section A: Humanities and Social Sciences. 2017;77(9-A(E)).

302. Low LK, Martin K, Sampselle C, Guthrie B, Oakley D. Adolescents' experiences of childbirth: Contrasts with adults. J Midwifery Women Health. 2003;48(3):192-8. doi: 10.1016/s1526-9523(03)00091-6.

303. Lupton DC. Mirrors, ghosts, and recollections: Exploring the intergenerational transmission of depression. Dissertation Abstracts International: Section B: The Sciences and Engineering. 2014;74(8-B(E)).

304. Lutenbacher M, Cohen A, Conner NM. Breaking the cycle of family violence: understanding the perceptions of battered women. J Pediatr Health Care. 2004;18(5):236-43.

305. Lutz KF. Abuse experiences, perceptions, and associated decisions during the childbearing cycle. West J Nurs Res. 2005;27(7):802-24; discussion 25-30.

306. Lyndon A, Malana J, Hedli LC, Sherman J, Lee HC. Thematic Analysis of Women's Perspectives on the Meaning of Safety During Hospital-Based Birth. Jognn. 2018;47(3):324-32. doi: 10.1016/j.jogn.2018.02.008.

307. Maker AH, Buttenheim M. Parenting difficulties in sexual-abuse survivors: A theoretical framework with dual psychodynamic and cognitive-behavioral strategies for intervention. Psychotherapy. 2000;37(2):159-70. doi: <http://dx.doi.org/10.1037/h0087796>.

308. Marina NS. The experiences of African American men who witnessed domestic violence growing up. Dissertation Abstracts International: Section B: The Sciences and Engineering. 2014;75(3-B(E)).

309. Markert F. The cultural revolution-A traumatic Chinese experience and subsequent transgenerational transmission: Some thoughts about inter-cultural interpretation. International Journal of Applied Psychoanalytic Studies. 2011;8(3):239-48. doi: <http://dx.doi.org/10.1002/aps.306>.

310. Martin JA, Elmer E. Battered children grown up: a follow-up study of individuals severely maltreated as children. Child Abuse Negl. 1992;16(1):75-87.

311. Martinez Burr P. Effects of intimate partner violence on maternal reflective functioning and attachment relationship in Hispanic/Latina women and their preschool-age children: A qualitative study. Dissertation Abstracts International: Section B: The Sciences and Engineering. 2015;76(2-B(E)).

312. Martsolf DS, Draucker CB. The legacy of childhood sexual abuse and family adversity. J Nurs Scholarsh. 2008;40(4):333-40. doi: <https://dx.doi.org/10.1111/j.1547-5069.2008.00247.x>.

313. Mason WA, Rice MJ, Records K. The lived experience of postpartum depression in a psychiatric population. Perspect Psychiatr Care. 2005;41(2):52-61. doi: 10.1111/j.1744-6163.2005.00011.x.

314. Mathews S, Jewkes R, Abrahams N. 'I HAD A HARD LIFE' Exploring Childhood Adversity in the Shaping of Masculinities among Men Who Killed an Intimate Partner in South Africa. British Journal of Criminology. 2011;51(6):960-77. doi: 10.1093/bjc/azr051.

315. Mbonye M, Nalukenge W, Nakamanya S, Nalusiba B, King R, Vandepitte J, et al. Gender inequity in the lives of women involved in sex work in Kampala, Uganda. J Int AIDS Soc. 2012;15:9. doi: 10.7448/ias.15.3.17365.

316. McComish JF, Greenberg R, Kent-Bryant J, Chruscial HL, Ager J, Hines F, et al. Evaluation of a grief group for women in residential substance abuse treatment. Subst Abus. 1999;20(1):45-58.

317. McConnell N, Barnard M, Taylor J. Caring Dads Safer Children: Families' perspectives on an intervention for maltreating fathers. Psychology of Violence. 2017;7(3):406-16. doi: <http://dx.doi.org/10.1037/vio0000105>.

318. McGaw VE, Reupert AE, Maybery D. Parenting with posttraumatic stress disorder: A veteran's experience. Traumatology. 2018;24(4):255-62. doi: <http://dx.doi.org/10.1037/trm0000154>.

319. McGhan C. Repeated acquisition of sexually transmitted infections: feelings, perceptions, and explanations of adolescent girls: University of Florida; 2005.

320. McGhee J, Waterhouse L. The Lady and the Pram: Women in Child Protection. British Journal of Social Work. 2017;47(6):1652-68. doi: 10.1093/bjsw/bcx100.

321. McMahon TJ, Winkel JD, Rounsaville BJ. Drug abuse and responsible fathering: a comparative study of men enrolled in methadone maintenance treatment. Addiction. 2008;103(2):269-83. doi: <https://dx.doi.org/10.1111/j.1360-0443.2007.02075.x>.

322. McWey LM, Pazdera AL, Vennum A, Wojciak AS. Intergenerational patterns of maltreatment in families at risk for foster care. Journal of Marital and Family Therapy. 2013;39(2):133-47. doi: <http://dx.doi.org/10.1111/j.1752-0606.2012.00289.x>.

323. Meadows-Oliver M. Homeless adolescent mothers: a metasynthesis of their life experiences. J Pediatr Nurs. 2006;21(5):340-9.

324. Meaney S, Lutomski JE, L OC, K OD, Greene RA. Women's experience of maternal morbidity: a qualitative analysis. BMC Pregnancy Childbirth. 2016;16(1):184. doi: <https://dx.doi.org/10.1186/s12884-016-0974-0>.

325. Meek R. The parenting possible selves of young fathers in prison. Psychology, Crime & Law. 2007;13(4):371-82. doi: <http://dx.doi.org/10.1080/10683160601060614>.

326. Mehta PK, Carter T, Vinoya C, Kangovi S, Srinivas SK. Understanding High Utilization of Unscheduled Care in Pregnant Women of Low Socioeconomic Status. Women's Health Issues. 2017;27(4):441-8. doi: 10.1016/j.whi.2017.01.007.

327. Menashe A, Possick C, Buchbinder E. Between the maternal and the professional: The impact of being a child welfare officer on motherhood. Child & Family Social Work. 2014;19(4):391-400. doi: <http://dx.doi.org/10.1111/cfs.12029>.

328. Mendel WE. Examining pathways between maternal childhood circumstances and self-reported pregnancy related stress among primiparous women. Dissertation Abstracts International Section A: Humanities and Social Sciences. 2016;77(2-A(E)).

329. Mendis K. Collecting data from mothers who have experienced childhood family violence with the use of a feminist methodology. Qualitative Social Work. 2009;8(3):377–90.

330. Meredith P, Wilson T, Branjerdporn G, Strong J, Desha L. "Not just a normal mum": a qualitative investigation of a support service for women who are pregnant subsequent to perinatal loss. Bmc Pregnancy and Childbirth. 2017;17:12. doi: 10.1186/s12884-016-1200-9.

331. Milch W. Intergenerational transmission of attachment-relationship: Following a mother and her twins via video tape. Pregnancy, birth to three years. Neuropsychiatrie de l'Enfance et de l'Adolescence. 2012;1):S20. doi: <http://dx.doi.org/10.1016/j.neurenf.2012.05.042>.

332. Miller LA. When it simply won't go away. J Perinat Neonatal Nurs. 2011;25(1):86-7. doi: <https://dx.doi.org/10.1097/JPN.0b013e31820759f0>.

333. Milligan R, Wingrove BK, Richards L, Rodan M, Monroe-Lord L, Jackson V, et al. Perceptions about prenatal care: views of urban vulnerable groups. BMC Public Health. 2002;2:25.

334. Mohler E, Resch F, Cierpka A, Cierpka M. The early appearance and intergenerational transmission of maternal traumatic experiences in the context of mother-infant interaction. Journal of Child Psychotherapy. 2001;27(3):257-71.

335. Monaghan-Blout SM. A different kind of parent: Resisting the intergenerational legacy of maltreatment. Dissertation Abstracts International: Section B: The Sciences and Engineering. 1999;60(2-B):0838.

336. Montgomery E. Voicing the silence: the maternity care experiences of women who were sexually abused in childhood. RCM Midwives. 2015;18:36.

337. Moore D, Ayers S. Virtual voices: social support and stigma in postnatal mental illness Internet forums. Psychol Health Med. 2017;22(5):546-51. doi: <https://dx.doi.org/10.1080/13548506.2016.1189580>.

338. Moran BA. The childbirth experience of abused women: University of Virginia; 2004.

339. Motley RR. Adult survivors of childhood sexual abuse: Characteristics of their mother-child relationships. Dissertation Abstracts International Section A: Humanities and Social Sciences. 1995;56(2-A):0464.

340. Moulthrop DL. Attitudes and expectancies of mothers who were abused as children. Dissertation Abstracts International. 1982;42(7-B):2996.

341. Muchena G. Men's experiences of partners' postnatal mental illness. Nurs Times. 2007;103(48):32-3.

342. Muldowney AM. Manifestation of the failure to thrive syndrome through the unconscious communication of unresolved loss in the mother/child dyad. Dissertation Abstracts International: Section B: The Sciences and Engineering. 1996;57(2-B):1505.

343. Murphy SA, Johnson L, Lohan J. Finding meaning in a child's violent death: A five-year propective analysis of parents' personal narratives and empirical data. Death Stud. 2003;27(5):381-404. doi: <http://dx.doi.org/10.1080/07481180302879>.

344. Murphy A. Daughters' perspectives on maternal substance abuse: Pledge to be a different kind of mother. A grounded theory study. Dissertation Abstracts International: Section B: The Sciences and Engineering. 2004;65(5-B):2640.

345. Murphy H, Strong J. Just another ordinary bad birth? A narrative analysis of first time mothers' traumatic birth experiences. Health Care Women Int. 2018;39(6):619-43. doi: 10.1080/07399332.2018.1442838.

346. Naples NA. ACTIVIST MOTHERING - CROSS-GENERATIONAL CONTINUITY IN THE COMMUNITY WORK OF WOMEN FROM LOW-INCOME URBAN NEIGHBORHOODS. Gend Soc. 1992;6(3):441-63. doi: 10.1177/089124392006003006.

347. Nash C, Morris J, Goodman B. A study describing mothers' opinions of the crying behaviour of infants under one year of age. Child Abuse Review. 2008;17:191-200.

348. Nellsch EM. The effect of childhood experiences, stress, and social support on parental attributions related to the dynamics of physical child abuse. Dissertation Abstracts International. 1992;53(1-B):549.

349. Nelson MK. Mothering others' children: The experiences of family day-care providers. Signs. 1990;15(3):586-605. doi: <http://dx.doi.org/10.1086/494611>.

350. Newby DH. Intergenerational caregiving: transition from grandparent to parent: BOSTON COLLEGE; 1993.

351. Newton BJ. An Aboriginal community's perceptions and experiences of child neglect in a rural town. Aust J Soc Iss. 2017;52(3):262-77. doi: 10.1002/ajs4.18.

352. Ney T, Stoltz J-A, Maloney M. Voice, power and discourse: Experiences of participants in family group conferences in the context of child protection. Journal of Social Work. 2013;13(2):184-202. doi: 10.1177/1468017311410514.

353. Nicholls K, Ayers S. Childbirth-related post-traumatic stress disorder in couples: a qualitative study. Br J Health Psychol. 2007;12(Pt 4):491-509.

354. Nicolai SS, Saus M. Acknowledging the past while looking to the future: conceptualizing indigenous child trauma. Child Welfare. 2013;92(4):55-74.

355. Nixon KL, Radtke HL, Tutty LM. “Every day it takes a piece of you away”: experiences of grief and loss among abused mothers involved with child protective services. Journal of Public Child Welfare. 2013;7(2):172-93.

356. Noble A. Birth Crisis. British Journal of Midwifery. 2007;15(4):239-.

357. North CS, Thompson SJ, Smith EM, Kyburz LM. Violence in the lives of homeless mothers in a substance abuse treatment program: A descriptive study. J Interpers Violence. 1996;11(2):234-49. doi: 10.1177/088626096011002007.

358. Nyberg K, Lindberg I, Ohrling K. Midwives' experience of encountering women with posttraumatic stress symptoms after childbirth. Sex Reprod Healthc. 2010;1(2):55-60. doi: <https://dx.doi.org/10.1016/j.srhc.2010.01.003>.

359. Onyango MA, Burkhardt G, Scott J, Rouhani S, Haider S, Greiner A, et al. A Qualitative Analysis of Disclosure Patterns among Women with Sexual Violence-Related Pregnancies in Eastern Democratic Republic of Congo. PLoS ONE. 2016;11(10):e0164631. doi: <https://dx.doi.org/10.1371/journal.pone.0164631>.

360. O'Reilly R, Peters K, Beale B, Jackson D. Women's experiences of recovery from childbirth: focus on pelvis problems that extend beyond the puerperium. J Clin Nurs. 2009;18(14):2013-9. doi: 10.1111/j.1365-2702.2008.02755.x.

361. O'Reilly R, Wilkes L, Luck L, Jackson D. Being Parents and Workers: Qualitative Insights from Child Protection Workers. Child Abuse Review. 2014;23(5):311-23. doi: 10.1002/car.2254.

362. Pajulo M, Pyykkonen N, Kalland M, Sinkkonen J, Helenius H, Punamaki R-L, et al. Substance-abusing mothers in residential treatment with their babies: Importance of pre- and postnatal maternal reflective functioning. Infant Ment Health J. 2012;33(1):70-81. doi: <http://dx.doi.org/10.1002/imhj.20342>.

363. Palacios JF. Sharing stories: understanding early childbearing among reservation-based Native American women: University of California, San Francisco; 2008.

364. Palacios J, Kennedy HP. Reflections of Native American teen mothers. J Obstet Gynecol Neonatal Nurs. 2010;39(4):425-34. doi: <https://dx.doi.org/10.1111/j.1552-6909.2010.01149.x>.

365. Paluzzi P, Kahn A. The impact of child maltreatment and family violence on the sexual, reproductive, and parenting behaviors of young men. Prevention Researcher. 2007;14:8-10.

366. Panchanadeswaran S, Jayasundara D. Experiences of drug use and parenting among women in substance abuse treatment: An exploratory study. Journal of Human Behavior in the Social Environment. 2012;22(8):971-87. doi: <http://dx.doi.org/10.1080/10911359.2012.707943>.

367. Paredes M, Leifer M, Kilbane T. Maternal variables related to sexually abused children's functioning. Child Abuse Negl. 2001;25(9):1159-76.

368. Paris R. "For the dream of being here, one sacrifices...": voices of immigrant mothers in a home visiting program. Am J Orthopsychiatry. 2008;78(2):141-51. doi: <https://dx.doi.org/10.1037/0002-9432.78.2.141>.

369. Paris R, Herriott A, Holt M, Gould K. Differential responsiveness to a parenting intervention for mothers in substance abuse treatment. Child Abuse Negl. 2015;50:206-17. doi: <https://dx.doi.org/10.1016/j.chiabu.2015.09.007>.

370. Pasalich DS, Cyr M, Zheng Y, McMahon RJ, Spieker SJ. Child abuse history in teen mothers and parent-child risk processes for offspring externalizing problems. Child Abuse Negl. 2016;56:89-98. doi: <https://dx.doi.org/10.1016/j.chiabu.2016.04.011>.

371. Peled E, Gil IB. The mothering perceptions of women abused by their partner. Violence Against Women. 2011;17(4):457-79. doi: <https://dx.doi.org/10.1177/1077801211404676>.

372. Perel G, Peled E. The fathering of violent men: constriction and yearning. Violence Against Women. 2008;14(4):457-82. doi: <https://dx.doi.org/10.1177/1077801208314846>.

373. Perry R, Murphy M, Haider S, Harwood B. “One Problem Became Another”: Disclosure of Rape-Related Pregnancy in the Abortion Care Setting. Women's Health Issues. 2015;25(5):470-5. doi: 10.1016/j.whi.2015.05.004.

374. Perry TE, Hassevoort L, Petrusak J. Care networks in play: Understanding death of a parent as a contributing factor to homelessness. Journal of Human Behavior in the Social Environment. 2017;27(7):656-68. doi: <http://dx.doi.org/10.1080/10911359.2017.1319316>.

375. Peter T. Hearing 'silent voices': Examining mother-daughter sexual abuse. Dissertation Abstracts International Section A: Humanities and Social Sciences. 2006;67(2-A):743.

376. Peter T. Speaking about the unspeakable: exploring the impact of mother-daughter sexual abuse. Violence Against Women. 2008;14(9):1033-53. doi: <https://dx.doi.org/10.1177/1077801208322057>.

377. Pettersen KT. A study of shame from sexual abuse within the context of a Norwegian incest center. J Child Sex Abuse. 2013;22(6):677-94. doi: <https://dx.doi.org/10.1080/10538712.2013.811139>.

378. Pieh-Holder KL, Callahan C, Young P. Qualitative needs assessment: healthcare experiences of underserved populations in Montgomery County, Virginia, USA. Rural Remote Health. 2012;12:1816.

379. Pierre JY. Transgenerational transmission of trauma in Haitian families. Dissertation Abstracts International: Section B: The Sciences and Engineering. 2017;78(5-B(E)).

380. Price-Robertson R. Child sexual abuse, masculinity and fatherhood. Journal of Family Studies. 2012;18(2-3):130-42. doi: <http://dx.doi.org/10.5172/jfs.2012.18.2-3.130>.

381. Priddis H, Dahlen H, Keedle H. The Perfect Storm of Trauma: The experiences of women who have experienced birth trauma and subsequently accessed residential parenting services in Australia...Abstracts for the Australian College of Midwives National Conference, 30 October - 2 November 2017, Adelaide, South Australia, Australia - Calling all Midwives: 'The truth is out there!'. Women & Birth. 2017;30:10-. doi: 10.1016/j.wombi.2017.08.027.

382. Priddis HS, Keedle H, Dahlen H. The Perfect Storm of Trauma: The experiences of women who have experienced birth trauma and subsequently accessed residential parenting services in Australia. Women & Birth. 2018;31(1):17-24. doi: 10.1016/j.wombi.2017.06.007.

383. Psaila K, Kruske S, Fowler C, Homer C, Schmied V. Smoothing out the transition of care between maternity and child and family health services: perspectives of child and family health nurses and midwives'. Bmc Pregnancy and Childbirth. 2014;14:13. doi: 10.1186/1471-2393-14-151.

384. Raj A, Sabarwal S, Decker M, Nair S, Jethva M, Krishnan S, et al. Abuse from In-Laws during Pregnancy and Post-Partum: Qualitative and Quantitative Findings from Low-income Mothers of Infants in Mumbai, India. Matern Child Health J. 2011;15(6):700-12. doi: 10.1007/s10995-010-0651-2.

385. Rajiva M. "Better lives": The transgenerational positioning of social mobility in the South Asian Canadian diaspora. Women's Studies International Forum. 2013;36:16-26. doi: <http://dx.doi.org/10.1016/j.wsif.2012.10.010>.

386. Rees S, Thorpe R, Tol W, Fonseca M, Silove D. Testing a cycle of family violence model in conflict-affected, low-income countries: a qualitative study from Timor-Leste. Soc Sci Med. 2015;130:284-91. doi: <https://dx.doi.org/10.1016/j.socscimed.2015.02.013>.

387. Reeves EA, Humphreys JC. Describing the healthcare experiences and strategies of women survivors of violence. J Clin Nurs. 2018;27(5-6):1170-82. doi: 10.1111/jocn.14152.

388. Remez A. Working with traumatized mothers and their babies in a residential drug rehabilitation facility. Journal of Infant, Child & Adolescent Psychotherapy. 2014;13(1):64-74. doi: <http://dx.doi.org/10.1080/15289168.2014.880292>.

389. Renker PR. "Keep a blank face. I need to tell you what has been happening to me.": Teens' stories of abuse and violence before and during pregnancy. MCN: The American Journal of Maternal/Child Nursing. 2002;27(2):109-16. doi: <http://dx.doi.org/10.1097/00005721-200203000-00011>.

390. Renner LM, Slack KS. Intimate partner violence and child maltreatment: understanding intra- and intergenerational connections. Child Abuse Negl. 2006;30(6):599-617.

391. Rice H, Warland J. Bearing witness: midwives experiences of witnessing traumatic birth. Midwifery. 2013;29(9):1056-63. doi: <https://dx.doi.org/10.1016/j.midw.2012.12.003>.

392. Richards J, Graham R, Embleton ND, Campbell C, Rankin J. Mothers' perspectives on the perinatal loss of a co-twin: a qualitative study. BMC Pregnancy Childbirth. 2015;15:143. doi: <https://dx.doi.org/10.1186/s12884-015-0579-z>.

393. Rigg EC, Schmied V, Peters K, Dahlen HG. Why do women choose an unregulated birth worker to birth at home in Australia: a qualitative study. Bmc Pregnancy and Childbirth. 2017;17:14. doi: 10.1186/s12884-017-1281-0.

394. Rizo C, Reynolds A, Macy R, Ermentrout D. Parenting and Safety Program for System-Involved Female Survivors of Intimate Partner Violence: A Qualitative Follow-up Study. Journal of Family Violence. 2016;31(7):833-48. doi: 10.1007/s10896-016-9833-z.

395. Rollans M, Schmied V, Kemp L, Meade T. Digging over that old ground: an Australian perspective of women's experience of psychosocial assessment and depression screening in pregnancy and following birth. BMC Womens Health. 2013;13:18. doi: <https://dx.doi.org/10.1186/1472-6874-13-18>.

396. Roman MW, Hall JM, Bolton KS. Nurturing natural resources: The ecology of interpersonal relationships in women who have thrived despite childhood maltreatment. ANS Adv Nurs Sci. 2008;31(3):184-97. doi: <http://dx.doi.org/10.1097/01.ANS.0000334282.96601.82>.

397. Rose A. Effects of childhood sexual abuse on childbirth: one woman's story. Birth. 1992;19(4):214-8.

398. Rose Z. The tree of life: a midwife's experience in the Philippines. British Journal of Midwifery. 2001;9(10):646-9.

399. Roseth I, Bongaardt R, Binder P-E. A case study of a mother's intertwining experiences with incest and postpartum depression. Int J Qual Stud Health Well-being. 2011;6(3):1-9. doi: <http://dx.doi.org/10.3402/qhw.v6i3.7244>.

400. Rossman B, Rea JG. The relation of parenting styles and inconsistencies to adaptive functioning for children in conflictual and violent families. Journal of Family Violence. 2005;20(5):261-77. doi: <http://dx.doi.org/10.1007/s10896-005-6603-8>.

401. Roth-Howe D. Wrestling with legacy: an intergenerational, cross-cultural response to the Holocaust. Smith College Studies in Social Work (Taylor & Francis Ltd). 2007;77(2/3):7-24.

402. Sachs B, Hall LA, Pietrukowicz MA. Moving beyond survival: coping behaviors of low-income single mothers. J Psychiatr Ment Health Nurs. 1995;2(4):207-15. doi: 10.1111/j.1365-2850.1995.tb00059.x.

403. Salberg J. The texture of traumatic attachment: presence and ghostly absence in transgenerational transmission. Psychoanal Q. 2015;84(1):21-46. doi: <https://dx.doi.org/10.1002/j.2167-4086.2015.00002.x>.

404. Salomonsson B. An infant's experience of postnatal depression. Towards a psychoanalytic model. Journal of Child Psychotherapy. 2013;39(2):137-55. doi: <http://dx.doi.org/10.1080/0075417X.2013.806052>.

405. Salter M, Breckenridge J. Women, trauma and substance abuse: Understanding the experiences of female survivors of childhood abuse in alcohol and drug treatment. International Journal of Social Welfare. 2014;23(2):165-73. doi: <http://dx.doi.org/10.1111/ijsw.12045>.

406. Salvi LM. Assimilating the voices of abuse: Towards building a theory of intergenerational transmission. Dissertation Abstracts International: Section B: The Sciences and Engineering. 2008;69(2-B):1364.

407. Santoro E, Stagni-Brenca E, Olivari MG, Confalonieri E, Di Blasio P. Childbirth Narratives of Women With Posttraumatic Stress Symptoms in the Postpartum Period. JOGNN: Journal of Obstetric, Gynecologic & Neonatal Nursing. 2018;47(3):333-41. doi: 10.1016/j.jogn.2018.02.009.

408. Saunders J, Fivush R. Reshaping Memories Through Conversations: Considering the Influence of Others on Historical Memories of Abuse. Applied Cognitive Psychology. 2015;29(6):789-90. doi: <http://dx.doi.org/10.1002/acp.3185>.

409. Sawyer A, Rabe H, Abbott J, Gyte G, Duley L, Ayers S, et al. Parents' experiences and satisfaction with care during the birth of their very preterm baby: a qualitative study. Bjog-an International Journal of Obstetrics and Gynaecology. 2013;120(5):637-43. doi: 10.1111/1471-0528.12104.

410. Schechter DS, Kaminer T, Grienenberger JF, Amat J. Fits and starts: A mother-infant case-study involving intergenerational violent trauma and pseudoseizures across three generations. Infant Ment Health J. 2003;24(5):510-22. doi: <http://dx.doi.org/10.1002/imhj.10070>.

411. Schechter DS. Witch-mother is which? The potential role of the analyst in facilitating authentic motherhood. International Forum of Psychoanalysis. 2017;26(1):10-21. doi: <http://dx.doi.org/10.1080/0803706X.2016.1195511>.

412. Schimmenti A. Unveiling the hidden self: Developmental trauma and pathological shame. Psychodynamic Practice: Individuals, Groups and Organisations. 2012;18(2):195-211. doi: <http://dx.doi.org/10.1080/14753634.2012.664873>.

413. Schlesinger G. A brief history of ghosts: Commentary on paper by Laurel Moldawsky Silber. Psychoanalytic Dialogues. 2012;22(1):129-38. doi: <http://dx.doi.org/10.1080/10481885.2012.646627>.

414. Schleske G. Imaginative and real child. On the impact of the phantasies of mothers on early development and the dynamic of child abuse. Zeitschrift fur Psychoanalytische Theorie und Praxis / Journal for Psychoanalytical Theory and Practice. 1999;14(4):438-63.

415. Schroll AM, Kjaergaard H, Midtgaard J. Encountering abuse in health care; lifetime experiences in postnatal women - a qualitative study. BMC Pregnancy Childbirth. 2013;13:74. doi: <https://dx.doi.org/10.1186/1471-2393-13-74>.

416. Schumacher B. 'I can't live without my child': Motherhood as a 'solution' to early trauma. British Journal of Psychotherapy. 2008;24(3):317-27. doi: <http://dx.doi.org/10.1111/j.1752-0118.2008.00088.x>.

417. Seamans CL, Rubin LJ, Stabb SD. Women domestic violence offenders: lessons of violence and survival. J Trauma Dissociation. 2007;8(2):47-68.

418. Searle J, Goldberg L, Aston M, Burrow S. Accessing new understandings of trauma-informed care with queer birthing women in a rural context. J Clin Nurs. 2017;26(21-22):3576-87. doi: <https://dx.doi.org/10.1111/jocn.13727>.

419. Setterberg S. The fantasmatic and imaginary child of the pregnant woman. Shanghai Archives of Psychiatry. 2017;29(3):161-70.

420. Shahram SZ, Bottorff JL, Oelke ND, Kurtz DL, Thomas V, Spittal PM, et al. Mapping the social determinants of substance use for pregnant-involved young Aboriginal women. Int J Qual Stud Health Well-being. 2017;12(1):1275155. doi: <https://dx.doi.org/10.1080/17482631.2016.1275155>.

421. Shahram SZ, Bottorff JL, Kurtz DL, Oelke ND, Thomas V, Spittal PM, et al. Understanding the Life Histories of Pregnant-Involved Young Aboriginal Women With Substance Use Experiences in Three Canadian Cities. Qual Health Res. 2017;27(2):249-59.

422. Sheen K, Spiby H, Slade P. The experience and impact of traumatic perinatal event experiences in midwives: A qualitative investigation. Int J Nurs Stud. 2016;53:61-72. doi: <https://dx.doi.org/10.1016/j.ijnurstu.2015.10.003>.

423. Sheen K, Spiby H, Slade P. What are the characteristics of perinatal events perceived to be traumatic by midwives? Midwifery. 2016;40:55-61. doi: <https://dx.doi.org/10.1016/j.midw.2016.06.007>.

424. Sherman MD, Gress Smith JL, Straits-Troster K, Larsen JL, Gewirtz A. Veterans' perceptions of the impact of PTSD on their parenting and children. Psychol Serv. 2016;13(4):401-10.

425. Shimoda K, Horiuchi S, Leshabari S, Shimpuku Y. Midwives' respect and disrespect of women during facility-based childbirth in urban Tanzania: a qualitative study. Reprod Health. 2018;15:13. doi: 10.1186/s12978-017-0447-6.

426. Siegel L. A mother learns to enjoy her baby: Parent-infant psychotherapy and art therapy in the treatment of intergenerational separation-individuation struggles. Infant Observation. 2011;14(1):61-74. doi: <http://dx.doi.org/10.1080/13698036.2011.552611>.

427. Sigurdardottir S, Halldorsdottir S, Bender SS. Deep and almost unbearable suffering: consequences of childhood sexual abuse for men's health and well-being. Scand J Caring Sci. 2012;26(4):688-97. doi: 10.1111/j.1471-6712.2012.00981.x.

428. Silva-Martínez E. "Allow Me to Speak": Stories of Courage Among Immigrant Latina Survivors of Intimate Partner Violence. Affilia: Journal of Women & Social Work. 2017;32(4):446-60. doi: 10.1177/0886109917721140.

429. Simkin P. Review of Survivor moms Women's stories of birthing, mothering and healing after sexual abuse. Birth: Issues in Perinatal Care. 2010;37(1):81-2. doi: <http://dx.doi.org/10.1111/j.1523-536X.2009.00384_1.x>.

430. Simpkins R. Pregnancy trauma. The effects of sexual abuse on childbearing: antenatal care. British Journal of Midwifery. 2006;14(3):162-3.

431. Skibniewski-Woods D. Mothering with a major mental illness. Community Pract. 2017;90(10):44-6.

432. Skinner AC. Power, control, and the "difficult" patient: hidden dimensions to caring for survivors of sexual abuse. J Midwifery Womens Health. 2010;55(2):181-2. doi: <https://dx.doi.org/10.1016/j.jmwh.2009.08.001>.

433. Skinner EM, Barnett B, Dietz HP. Psychological consequences of pelvic floor trauma following vaginal birth: a qualitative study from two Australian tertiary maternity units. Archives of Womens Mental Health. 2018;21(3):341-51. doi: 10.1007/s00737-017-0802-1.

434. Slesnick N, Guo X. Treatment desires and symptomatology among substance-abusing homeless mothers: what I want versus what I need. J Behav Health Serv Res. 2013;40(2):156-68. doi: <https://dx.doi.org/10.1007/s11414-012-9300-4>.

435. Smid M, Bourgois P, Auerswald CL. The challenge of pregnancy among homeless youth: reclaiming a lost opportunity. J Health Care Poor Underserved. 2010;21(2 Suppl):140-56. doi: <https://dx.doi.org/10.1353/hpu.0.0318>.

436. Smith CS. Substance abuse, chronic sorrow, and mothering loss: relapse triggers among female victims of child abuse. J Pediatr Nurs. 2009;24(5):401-12. doi: <https://dx.doi.org/10.1016/j.pedn.2007.11.003>.

437. Smith A, Krisman K, Strozier AL, Marley MA. Breaking Through the Bars: Exploring the Experiences of Addicted Incarcerated Parents Whose Children Are Cared For By Relatives. Families in Society. 2004;85(2):187-95. doi: <http://dx.doi.org/10.1606/1044-3894.329>.

438. SmithBattle L. Gaining ground from a family and cultural legacy: a teen mother's story of repairing the world. Fam Process. 2008;47(4):521-35.

439. Smith Lester VD, Kautz DD. Life Review. J Gerontol Nurs. 2017;43(2):65-7. doi: 10.3928/00989134-20161109-02.

440. Smith MK. A recovery story that heals. Arctic Anthropol. 2003;40(2):83-6.

441. Smith Stover C, Kahn M. Family of origin influences on the parenting of men with co-occurring substance abuse and intimate partner violence. Adv Dual Diagn. 2013;6(2):84-94.

442. B. S-M, N. S. “Don't leave me hanging”: homeless mothers' perceptions of service providers. Journal of Social Service Research. 2011;37(5):457-68.

443. Song SJ, De Jong J, O'Hara R, Koopman C. Children of former child soldiers and never-conscripted civilians: A preliminary intergenerational study in burundi. Journal of Aggression, Maltreatment and Trauma. 2013;22(7):757-72. doi: <http://dx.doi.org/10.1080/10926771.2013.813881>.

444. Song SJ, Tol W, de Jong J. Indero: intergenerational trauma and resilience between Burundian former child soldiers and their children. Fam Process. 2014;53(2):239-51. doi: <https://dx.doi.org/10.1111/famp.12071>.

445. Souza JP, Cecatti JG, Parpinelli MA, Krupa F, Osis MJD. An emerging 'maternal near-miss syndrome': narratives of women who almost died during pregnancy and childbirth. Birth: Issues in Perinatal Care. 2009;36(2):149-58. doi: 10.1111/j.1523-536X.2009.00313.x.

446. Spangaro JM, Zwi AB, Poulos RG. "Persist. persist.": A qualitative study of women's decisions to disclose and their perceptions of the impact of routine screening for intimate partner violence. Psychology of Violence. 2011;1(2):150-62. doi: <http://dx.doi.org/10.1037/a0023136>.

447. Spangaro J, Koziol-McLain J, Zwi A, Rutherford A, Frail MA, Ruane J. Deciding to tell: Qualitative configurational analysis of decisions to disclose experience of intimate partner violence in antenatal care. Soc Sci Med. 2016;154:45-53. doi: <https://dx.doi.org/10.1016/j.socscimed.2016.02.032>.

448. Springer-Kremser M, Leithner K, Fischer M, Loffler-Stastka H. Gender and perversion--what constitutes a "bad mother". Arch Women Ment Health. 2003;6(2):109-14.

449. Stanley N, Miller P, Richardson Foster H. Engaging with children's and parents' perspectives on domestic violence. Child & Family Social Work. 2012;17(2):192-201. doi: <http://dx.doi.org/10.1111/j.1365-2206.2012.00832.x>.

450. Stanley N, Oram S, Jakobowitz S, Westwood J, Borschmann R, Zimmerman C, et al. The health needs and healthcare experiences of young people trafficked into the UK. Child Abuse Negl. 2016;59:100-10. doi: <https://dx.doi.org/10.1016/j.chiabu.2016.08.001>.

451. Stapleton H, Murphy R, Kildea S. Lost in translation: staff and interpreters' experiences of the edinburgh postnatal depression scale with women from refugee backgrounds. Issues Ment Health Nurs. 2013;34(9):648-57. doi: <https://dx.doi.org/10.3109/01612840.2013.804895>.

452. Stern ZY. The experience of parenthood among children of Holocaust survivors: Reworking a traumatic legacy. Dissertation Abstracts International: Section B: The Sciences and Engineering. 1995;56(6-B):3465.

453. Stern T. The development of reflective functioning in a mother traumatized by past and present events: Facilitating change in the parent-infant relationship. Journal of Infant, Child & Adolescent Psychotherapy. 2014;13(1):24-36. doi: <http://dx.doi.org/10.1080/15289168.2014.880291>.

454. Stewart M, Dennis CL, Kariwo M, Kushner KE, Letourneau N, Makumbe K, et al. Challenges Faced by Refugee New Parents from Africa in Canada. Journal of Immigrant and Minority Health. 2015;17(4):1146-56. doi: 10.1007/s10903-014-0062-3.

455. Stidham AW. Survivors of sexual violence and altruism: designing a typology: Kent State University; 2009.

456. Stockl H, Gardner F. Women's perceptions of how pregnancy influences the context of intimate partner violence in Germany. Cult Health Sex. 2013;15(10):1206-20. doi: 10.1080/13691058.2013.813969.

457. Strong DD, Bean RA, Feinauer LL. Trauma, attachment and family therapy with grandfamilies: a model for treatment. Children and Youth Services Review. 2010;32(1):44-50.

458. Tait CL. Resituating the ethical gaze: government morality and the local worlds of impoverished Indigenous women. Int J Circumpolar Health. 2013;72(1). doi: <https://dx.doi.org/10.3402/ijch.v72i0.21207>.

459. Tapias M. Emotions and the intergenerational embodiment of social suffering in rural Bolivia. Med Anthropol Q. 2006;20(3):399-415.

460. Taylor J, Gibson B, Hurd K. Parental preschool choices and challenges when young children and their families experience homelessness. Children and Youth Services Review. 2015;56:68-75. doi: <http://dx.doi.org/10.1016/j.childyouth.2015.06.010>.

461. Telfeyan N. The attachment experiences of women with young children in women's substance abuse treatment: An exploratory study. Dissertation Abstracts International Section A: Humanities and Social Sciences. 2007;68(3-A):1163.

462. Thomas SP, Hall JM. Life trajectories of female child abuse survivors thriving in adulthood. Qual Health Res. 2008;18(2):149-66. doi: <https://dx.doi.org/10.1177/1049732307312201>.

463. Thomas SP, Bannister SC, Hall JM. Anger in the trajectory of healing from childhood maltreatment. Arch Psychiatr Nurs. 2012;26(3):169-80. Epub 2012/05/29. doi: 10.1016/j.apnu.2011.09.003.

464. Thomas KA, Mederos F, Rodriguez G. "It shakes you for the rest of your life": Low-income fathers' understanding of domestic violence and its impact on children. Psychology of Violence. 2018. doi: <http://dx.doi.org/10.1037/vio0000196>.

465. Tilley DS, Brackley M. Violent lives of women: critical points for intervention -- phase I focus groups. Perspect Psychiatr Care. 2004;40(4):157-70.

466. Tingberg B, Bredlov B, Ygge BM. Nurses' experience in clinical encounters with children experiencing abuse and their parents. J Clin Nurs. 2008;17(20):2718-24. doi: <https://dx.doi.org/10.1111/j.1365-2702.2008.02353.x>.

467. Titsworth KC. Moving beyond blame and shame: Rethinking mothering practices in an urban Appalachian community. Dissertation Abstracts International Section A: Humanities and Social Sciences. 2009;69(8-A):3045.

468. Tol WA, Reis R, Susanty D, de Jong JT. Communal violence and child psychosocial well-being: qualitative findings from Poso, Indonesia. Transcult Psychiatry. 2010;47(1):112-35. doi: <https://dx.doi.org/10.1177/1363461510364573>.

469. Torchalla I, Linden IA, Strehlau V, Neilson EK, Krausz M. "Like a lots happened with my whole childhood": violence, trauma, and addiction in pregnant and postpartum women from Vancouver's Downtown Eastside.[Erratum appears in Harm Reduct J. 2017 Sep 19;14 (1):65; PMID: 28927414]. Harm Reduct J. 2015;11:34. doi: <https://dx.doi.org/10.1186/1477-7517-11-34>.

470. Tracey N, Blake P, Warren B, Hardy H, Enfield S, Shein P. Will I be to my son my as father was to me? Narrative of a father with a premature baby. Journal of Child Psychotherapy. 1996;22(2):168-94.

471. Trad PV. The phenomenon of previewing and its contribution to the emerging sense of self. Psychoanal Rev. 1996;83(1):21-47.

472. Ueno M, Kayama M, Murashima S. How public health nurses understand mothers of abused and neglected children: the perception of 'Shindosa' in mothers. Japan Journal of Nursing Science. 2004;1(2):117-24.

473. Veale A, McKay S, Worthen M, Wessells MG. Participation as Principle and Tool in Social Reintegration: Young Mothers Formerly Associated with Armed Groups in Sierra Leone, Liberia, and Northern Uganda. Journal of Aggression, Maltreatment & Trauma. 2013;22(8):829-48. doi: 10.1080/10926771.2013.823635.

474. Veltkamp G, Brown P. The everyday risk work of Dutch child-healthcare professionals: inferring 'safe' and 'good' parenting through trust, as mediated by a lens of gender and class. Sociology of Health & Illness. 2017;39(8):1297-313. doi: 10.1111/1467-9566.12582.

475. Virokannas E. Identity categorization of motherhood in the context of drug abuse and child welfare services. Qualitative Social Work: Research and Practice. 2011;10(3):329-45. doi: <http://dx.doi.org/10.1177/1473325011408480>.

476. Volpe EM, Quinn CR, Resch K, Douglas V, Cerulli C. Assessing the feasibility and acceptability of Narrative Exposure Therapy to address IPV-related mental health in parenting and pregnant adolescents. Journal of Family Violence. 2017;32(4):439-52. doi: <http://dx.doi.org/10.1007/s10896-016-9818-y>.

477. Waldrop DLP. A qualitative study of grandparents raising grandchildren. Dissertation Abstracts International Section A: Humanities and Social Sciences. 2000;60(8-A):3140.

478. Walker ME. Because it is my heart: A qualitative case study of the influence of spirituality and creativity in the recovery of a mother and daughter from childhood incest trauma. Dissertation Abstracts International Section A: Humanities and Social Sciences. 2007;68(6-A):2340.

479. Walker RJ. 'If this isn't for my children, who is it for?' Exploring experiences of structural violence among migrant mothers who sell sex in Johannesburg. Families, Relationships and Societies. 2017;6(2):291-306.

480. Walters S, East L. The cycle of homelessness in the lives of young mothers: the diagnostic phase of an action research project. J Clin Nurs. 2001;10(2):171-9.

481. Wamoyi J, Wight D, Remes P. The structural influence of family and parenting on young people's sexual and reproductive health in rural northern Tanzania. Cult Health Sex. 2015;17(6):718-32. doi: 10.1080/13691058.2014.992044.

482. Wangerin GB. Maternal protective behaviors regarding sexual abuse in women with a history of childhood sexual abuse. Dissertation Abstracts International: Section B: The Sciences and Engineering. 1996;56(10-B):5424.

483. Wapinsky A. Opiate-dependent pregnant women's perceptions of their personal strengths, substance abuse and treatment histories: An appreciative inquiry approach. Dissertation Abstracts International: Section B: The Sciences and Engineering. 2016;77(3-B(E)).

484. Ward H, Skuse T, Munro ER. 'The best of times, the worst of times': young people's views of care and accommodation. Adoption & Fostering. 2005;29(1):8-17.

485. Watt MH, Dennis AC, Choi KW, Ciya N, Joska JA, Robertson C, et al. Impact of Sexual Trauma on HIV Care Engagement: Perspectives of Female Patients with Trauma Histories in Cape Town, South Africa. Aids and Behavior. 2017;21(11):3209-18. doi: 10.1007/s10461-016-1617-1.

486. Werner-Wilson RJ, Zimmerman TS, Whalen D. Resilient response to battering. Contemp Fam Ther. 2000;22(2):161-88. doi: 10.1023/a:1007777702757.

487. White G. You cope by breaking down in private: fathers and PTSD following childbirth. British Journal of Midwifery. 2007;15(1):39-45.

488. Wijnberg MH, Reding KM. Reclaiming a stress focus: The hassles of rural, poor single mothers. Fam Soc-J Contemp Hum Serv. 1999;80(5):506-15. doi: 10.1606/1044-3894.1480.

489. Willey VL. Changes in sexuality during pregnancy. Dissertation Abstracts International: Section B: The Sciences and Engineering. 2003;63(10-B):4930.

490. Williams JK, Hall JA. Stress and traumatic stress: how do past events influence current traumatic stress among mothers experiencing homelessness? Soc Work Res. 2009;33(4):199-207.

491. Williams AL, Merten MJ. Childhood adversity and development of self among mothers transitioning from homelessness to self-sufficiency. Journal of Social Service Research. 2015;41(3):398-412. doi: <http://dx.doi.org/10.1080/01488376.2015.1013171>.

492. Williamson V, Halligan SL, Coetzee B, Butler I, Tomlinson M, Skeen S, et al. Caregiver experiences of public services following child trauma exposure: A qualitative study. International Journal of Mental Health Systems. 2018;12:15. doi: <http://dx.doi.org/10.1186/s13033-018-0190-6>.

493. Wilson-Mitchell K, Bennett J, Stennett R. Psychological health and life experiences of pregnant adolescent mothers in Jamaica. Int J Environ Res Public Health. 2014;11(5):4729-44. doi: <https://dx.doi.org/10.3390/ijerph110504729>.

494. Woodhouse LD. Women with jagged edges: voices from a culture of substance abuse. Qual Health Res. 1992;2(3):262-81.

495. Wright MOD, Crawford E, Sebastian K. Positive resolution of childhood sexual abuse experiences: The role of coping, benefit-finding and meaning-making. Journal of Family Violence. 2007;22(7):597-608. doi: <http://dx.doi.org/10.1007/s10896-007-9111-1>.

496. Wuest J, Malcolm J, Merritt-Gray M. Daughters' obligation to care in the context of past abuse. Health Care Women Int. 2010;31(12):1047-67. doi: <https://dx.doi.org/10.1080/07399331003599563>.

497. Xie QW, Sun X, Chen M, Qiao DP, Chan KL. What prevents chinese parents from reporting possible cases of child sexual abuse to authority? A holistic-interactionistic approach. Child Abuse Negl. 2017;64:19-31. doi: <http://dx.doi.org/10.1016/j.chiabu.2016.12.006>.

498. Yaroslawitz SL, DeGrace BW, Sloop J, Arnold S, Hamilton TB. A study of family health in Chareidi second and third generation survivors of the Holocaust. Work. 2015;50(3):501-10. doi: <https://dx.doi.org/10.3233/WOR-141961>.

499. Yi CH, Lori J, Martyn K. Development of prenatal event history calendar for Black women. J Obstet Gynecol Neonatal Nurs. 2008;37(4):464-73. doi: <https://dx.doi.org/10.1111/j.1552-6909.2008.00255.x>.

500. Zanoni L, Warburton W, Bussey K, McMaugh A. Are all fathers in child protection families uncommitted, uninvolved and unable to change? Children and Youth Services Review. 2014;41:83-94. doi: <http://dx.doi.org/10.1016/j.childyouth.2014.03.014>.

501. Zanoni L, Warburton W, Bussey K, McMaugh A. Child protection fathers' experiences of childhood, intimate partner violence and parenting. Children and Youth Services Review. 2014;46:91-102. doi: <http://dx.doi.org/10.1016/j.childyouth.2014.08.009>.

502. Zeanah PD, Larrieu JA, Boris NW, Nagle GA. Nurse home visiting: Perspectives from nurses. Infant Ment Health J. 2006;27(1):41-54. doi: 10.1002/imhj.20079.

503. Zlotnick C, Robertson MJ, Wright MA. The impact of childhood foster care and other out-of-home placement on homeless women and their children. Child Abuse Negl. 1999;23(11):1057-68.
