# Supplementary material for: Healing The Past By Nurturing The Future: A qualitative systematic review and meta-synthesis of pregnancy, birth and early postpartum experiences and views of parents with a history of childhood maltreatment
Source: PLoS One. 2019 Dec 13;14(12):e0225441. doi: 10.1371/journal.pone.0225441 (PMC6910698; doi:10.1371/journal.pone.0225441)
Supplement: S8 Appendix — Provides a long version of draft qualitative findings with overinclusive list of supporting parent and study author quotes. (DOCX) [file pone.0225441.s008.docx]

## **S8 Appendix: Long draft version of qualitative findings with additional supporting parent and study author quotes**

## **Theme 1. *New beginnings*: Becoming a parent is an opportunity for ‘a fresh start’, to put the past behind them and move forward with hope for the future to create a new life for themselves and their child.**

This analytic theme incorporates three main descriptive subthemes; *new opportunities and motivation to change*; *hopes and dreams for the future*; and *wanting to parent differently*. Axial codes are highlighted in **bold**. Summary supporting quotes for parents (primary level data) are indented and provided in “*10 font italics*”, and review author quotes (secondary level data) are provided in “plain 10 font”, to differentiate between the primary and secondary sources. A more comprehensive set of supporting quotes for each theme are provided in S7 Appendix.

### 1.1 New opportunities and motivations for change

For many participants, pregnancy and the transition to parenthood was a turning point in their life trajectory, offering a **new opportunity or fresh start** for positive change and a “*normal*” life that was distinct from their past trauma [1-10]. Many parents viewed pregnancy and birth as a new beginning, a fresh start or a second chance for themselves, with some mothers describing a regenerative experience and a rebirth. This links to ‘*reweaving’ a future* and the *healing process of becoming a parent* (theme 7).

“*I was just happy, like oh my gosh! I didn’t think of it as my world was ending, you know, or life was ending, I thought of it, you know, as a new beginning for me and my child*.” [8]

“*It was almost a symptom of the trauma, like it was the only way I could feel like I was going to be able to go on and be better you know. I almost felt like it was the, one of the reasons why after like suffering abuse was to, I didn't really feel like, I don't know how to explain it, like I didn't see any other purpose in going on unless I had a child, so for me it was like almost like, well in a sense I would be able to start over by having this child*.” [7]

“Mary saw the pregnancy as a chance to escape the violence she experienced when she lived with her mother; she was also tired, she said, *‘tired of drinking and not living at home, and stuff’*. She wanted to change, and she said her partner helped her stop *‘kicking it’*, *‘hanging out with people and drinking and doing drugs’*.” [8]

The confirmation of pregnancy elicited a new sense of responsibility in expectant mothers to practice **self-care or take care of self for baby** and develop a healthier lifestyle [2, 3, 11].

“*I'm just trying to take better care of myself. So I started taking better care of myself, because I was very motivated to make sure she started out okay and that she had me there, and I had to be as close to full capacity as I could get, so I could be there for her*.” [3]

In some studies, pregnancy provided women the motivation to step away from risky behaviours including drug and alcohol use, and the opportunity for **maturing or changing behaviour for the baby**, such as “*settling down*” by finding a steady job, returning to school, and accessing services and stable housing [2, 6, 8].

### 1.2 Hopes and dreams for the future

Compared to participants’ past trauma experiences, pregnancy brought a new found **hopes or dreams for the future** [1, 5, 8-10, 12] and desires for parents. Feelings of hope and optimism were common among expectant mothers facing difficult circumstances (i.e. single, homeless or adolescent mothers) and were embedded in their motivation to create a **stable home** **life or family** [1, 2, 7, 8, 12] which differed from their own childhood. Parents also reported a **stable or safe relationship** being an important part of this [2].

“*I wanted stability. I want a safe environment. Not just a safe home, but a safe community... a safe environment... a safe, happy, healthy environment and not just but for him and me, but for our families as well.*” [2]

Expectant adolescent mothers **hoped their partner would be a good father** [8], and shared **hopes for their child** [4, 9], including around their appearance, personality and academic achievements. This links to *wanting to parent differently* (subtheme 1.3).

“*I imagined him like, I had this dream one night I had already had him and his older brother. They were all grown up and doing stuff, graduated from high school, went to college. Did what they were supposed to do. Got great jobs. Had a family and then you know, I was peaceful and you know I died in my sleep. It was peaceful to actually see that.*” [4]

Women with a history of sexual trauma hoped that their child would never experience sexual abuse.

*“I hope and pray things don’t happen to her the way that they did [for me] so that she will have a good life*.” [9]

In describing their hopes and desires, some parents expressed highly idealised notions of **a ‘perfect’ or idealised family** that was distinctly different from their own upbringing [1, 6]. Parents reflected on and ‘self-evaluated’ their parenting, with some suggesting an ideal family would have finely balanced and supportive relationships; a perfect understanding between mother and partner, and mother and child; a sense of stability and trust; and be free of violence [1, 6]. Pregnant women also highlighted the importance of **being a ‘good mother’** [1-3, 8], which was again related to their own unmet needs during childhood, and links to *changing roles and identities* (theme 2). Being a ‘good mother’ involved being present and focussed on the future, not the past and ‘moving on’ [13].

“*And I think a lot of that was motivated by what happened to me. And I spent a lot of time thinking about what it meant to be a mother. I spent a lot of time reading about it as best I could, to try to understand it before I had a daughter. And because I really wanted to be a good mother.*” [3]

Focusing on the future and being a ‘good mother’ were key to making positive life changes.

“*If you want to change your life you, you have to think about your child, about your happy family. And what a good mother you’re going to be. Concentrate on [the] positive. The past is past. I decided to live with my present, and just look into the future. And there is no bad in the future for me, I know.*” [1]

### 1.3 Wanting to parent differently

Enmeshed in parents’ hopes and dreams for the future was an intense **desire to not repeat the past** [2, 6, 8, 14, 15]. They **wanted to parent differently** [1, 2, 6, 7, 11, 15] and wanted their children to have a life unlike their own.

“*From the point when I started trying to conceive I was thinking about the baby. I was thinking how good is going to be his or her life. Not like mine*.” [1]

Mothers and fathers described turning their past abuse into a “*positive*” [15]. Their experiences taught them what not to do as they approach parenthood and instilled a desire to not repeat the past and break the cycle of abuse. However, parents described a lack of role models, and more about ‘what *not* to do’ rather than ‘what to do’*,* which links to challenges in **establishing healthy boundaries** (discussed below under *coping strategies*, subtheme 7.2) in their lives and their relationship with their child.

“*[My mum] put a lot things in perspective for me. I learned what not to do, who not to be, and how not to act and what not to do to my own kids, which I kind of am thankful for*.” [15]

“*I really want to raise my boys differently. Stop the cycle of abuse before it begins again*.” [6]

*“…I am very strict. I love them [talking about children from a former partner] to death and I do everything I can to spoil them alright. But I’ve been laid off twice and it’s difficult to make ends meet sometimes and sometimes I think that I have given them too much leeway on one aspect and then not enough leeway on another aspect and I’m trying to get back to a balancing point and let them be kids. However, when they act up, I have caught myself grabbing shoulders or arms…but I’m not going to physically abuse them.”[16]*

Parents also described challenges of feeling a **fear of repeating the past** [2, 4-6, 11, 14, 15], impacting on early attachment, which links to *safety* and *trusting others* (subtheme 6.1).

“*I had gotten pregnant with my second son, I was so afraid. I never sought therapy to sort myself out, but I was so afraid I was going to end up like my mother. And, or end up with somebody like my father or whatever. But, that just terrified me and the second time I got pregnant I thought about having an abortion cause I thought I was doing okay with one, but I didn’t want to have that added stress, you know?*” [11]

“*Sometimes when I am bathing her I wonder if my touch is appropriate, am I doing any unforeseen damage? I try to be as normal as possible. Even when I massage her after her bath I’m conscious of her genitals at all times and try to be careful not to touch them unnecessarily. I worry about teaching her about appropriate touch when she is older. I just worry like any mother would.*” [6]

Parents reflected on the type of abuse they had experienced (e.g. verbal abuse, physical abuse exposure to IPV) and resolved to never inflict that upon their own child [2, 8, 14, 15]. However, one study found some expectant mothers and fathers with a history of childhood physical abuse did intend to physically discipline their child (e.g. spanking) [15].

In several studies, parents talked about **wanting to be emotionally responsive** and caring with their child, and connect with their children as they grow up, often because they felt their own parents had been emotionally unavailable [2, 4, 8, 9, 17].

"*I remembered how I was not loved or wanted. I made a vow on the table with the placenta not even out, that I would be different. Things would be different. I will make a conscious effort to hug them. I don't think I remember ever once being hugged, no one ever said I love you. I'm going to be exactly opposite*." [17]

“*There was really nowhere that I felt I was safe and where I was loved just for me. Now I have my own my family, this is my chance to make a safe space not just for my daughter, but for me and her father, and to create memories that are happy and healthy... not sort of tainted*.” [2]

## **Theme 2. *Changing roles and identities*: Becoming a parent is a major life transition, influenced by perceptions of the parenting role.**

This analytic theme included several descriptive themes around: *mixed emotions in pregnancy and birth*; *striving to be a ‘good’ or ‘perfect’ parent*; *wanting to be ‘normal’*; and *knowledge and learning about parenting*.

### 2.1 Mixed emotions in pregnancy and birth

Mothers shared a range of **conflicting internal emotions about** **pregnancy** [1, 2, 4, 6-8, 18-20]. These experiences link with themes of new beginnings, relationships with self, and axial node of **lack of control** (discussed later). Many described feelings of excitement, elation and joy in **becoming pregnant** [2, 4, 8, 17, 21] although reactions were also often mixed. Conflicting emotions of **hopes and fears** [4] for the future, as well as excitement and uncertainty, worry, nervousness or fear were reported by expectant mothers as they considered the impending responsibility of parenthood [2, 4, 8, 17, 18], particularly for young or single mothers or women with an unplanned pregnancy, and for women with fertility concerns. Feelings of shame or embarrassment were common among pregnant adolescents [2, 4, 17]. Some women described the **impact of pregnancy on their current lifestyle** [2, 8] as a challenge.

Some women reflected on their past trauma experiences when describing their ambivalent feelings about becoming pregnant [1, 2, 6, 17].

“*When I found out I was pregnant... I was excited and then right away I said, ‘No. I can’t be. This isn’t right’. I do believe that my reaction relates back to being abused. I was abused when I was 14 and it put me back in that place. I felt ashamed and that it was my fault that it all happened. I felt like a little kid, like a young teenager who is pregnant and should not be. That’s pretty much what I felt like. Another big part of that was that my baby’s father was not sure whether he wanted to be a father at the time, so that did not help; that put a lot of stress on me*.” [2]

“*I was very anxious about what I could do to a baby because I was so mixed up and distraught on so many different levels. At some point, there was - it was almost like there was an answering consciousness - giving me a strong message that everything was going to be okay. I settled down and I started to think about whether it was a boy or a girl and started to engage with my pregnancy*.” [2]

One parent noted that the happiness she felt contributed to her ambivalence about her pregnancy.

"*I was actually pretty happy. It was the first time that I ever felt good about myself. It's hard to feel good. Me feeling good means that I have to be punished. Feeling good is not comfortable. It is extremely uncomfortable*." [17]

For many mothers, these feelings about becoming pregnant were strongly influenced by the quality of **relationships with their partner** (subtheme 3.4), as to whether the experience of becoming pregnant was described in a positive or negative way. Reactions of other family members to the pregnancy also had a significant impact on the experience, with reactions of adolescent family members being more mixed and parents describing feelings of **stigma and judgement** (subtheme 6.2).

**Postpartum adjustment** [6] was a particular challenge for many parents in this study, dealing with triggers and often with very limited support systems. This links to descriptive themes of *relationship with child/bonding* (3.2), *relationship with self/body* (3.3), *empowerment, choice and control* (5.1) *and distress symptoms* (7.1).

“*It [postpartum period] was such a difficult time for me. I just remember it being a really black place... almost like I was living in a fog. It was all a blur. I do remember that I had a really hard time. I just couldn’t cope with a demanding baby and still take care of myself. I just felt lost and had nothing left to give. I think that’s when it [feeling disconnected with her child] all started. I knew then that I wouldn’t be a good mom.*” [6]

### 2.2 Striving to be a ‘good’ or ‘perfect’ parent

This subtheme links to the descriptive subtheme of *hopes and dreams for the future (1.2)* previously discussed. The impact of women’s trauma history on their **negative self-belief** (discussed under *distress symptoms*, subtheme 7.1) and their perceptions of being able or ready to parent were evident from pre-conception and across the transition to parenthood. Many believed that they would not be a ‘good’ parent due to their trauma, or their body ‘holding the trauma’, contributing to their decision to terminate previous pregnancies [6] or **delaying pregnancy** [2, 3, 11, 22, 23]. Parents described a sense of ‘unworthiness’ which related to a sense of **fear** and **stigma or judgement,** discussed under *the world is unsafe* and *the external world* (subthemes 6.1 and 6.2).

“*I’d never had even crossed my mind at that point that I’d ever become a mother I just thought my mental health problems were too strong... I would be no good as a mother*.” [22]

“*I never wanted to be a mum... it happened anyway. I wasn’t going to be a mother because I just didn’t think I would be a very good Mum. I wasn’t very confident*.” [23]

“*I think part of the waiting was my subconscious knowing that there was no possible way I could raise a healthy child until I had dealt with my sexual abuse or at least had started to talk about it*.” [2]

One study [6] included a small number of survivors who were **childless by choice** because of their childhood sexual abuse. These women struggled with intimacy or fertility, yet also felt unable or unworthy of mothering. Their internal beliefs were amplified by other people’s perceptions that they were unable to be a ‘good’ mother or would be at risk of abusing their child given their abuse histories.

“*I believe this is true for so many of us, we believe that there is something about us that made that [abuse] happen and that we are really at high risk… Almost everybody could be talked into thinking you can’t possibly have children because you will do the same things to your kids that was done to you.*” [6]

Women also shared positive accounts of their changing identity. Becoming pregnant and the decision to keep the pregnancy gave some women a sense of **responsibility or purpose** [2-4, 6, 7, 13], “…*my purpose was to grow a baby and to be a mother so that was a huge shift*” [6]. As part of their new role, mothers reported a sense of responsibility associated with providing for their baby’s growth and emotional needs [6, 7, 24] but also had a heightened responsibility to take care of themselves for the sake of their child [2, 6], as previously discussed in subtheme 1.1.

“*If stay in bed and be depressed all day, then nobody is going to take care of this little girl. I just, you know, I have such a thing against abusers that I have to make sure that it doesn’t happen to my little girl. I have had to be a lot more responsible about how I think and about how I feel*.” [2]

Young single mothers also described their **determination** to rise above adversity and “*do what [they] needed to do*” to become the best mothers they could be [4]. Motherhood helped some women to **regain a sense of self-worth** and of belonging in the world [2, 8].

“*What’s so outrageous for me is that it’s the most normal thing in the world is to have children and a family. Like it’s so normal... but it’s a good normal. You get to understand, “Oh, that’s what my breasts are for”, “That’s why I have hips” ... it’s like all of the sexual connotations of women’s bodies - the curves and all of that - it’s so crude in a way. Because breasts are for breastfeeding, you know? Hips are for birthing a baby!*” [2]

Having felt trapped and fearful of their parents as a child, incest survivors were acutely aware of their own power over their child and particularly sensitive to their child’s needs and emotions [21]. This heightened sense of responsibility weighed heavily on some mothers and links to **fear of repeating the past** (previously discussed) and challenges establishing boundaries. In contrast, some mothers worried that their own past experiences would hamper their sensitivity as a parent [5].

There was considerable diversity in women’s perceptions of **being able or ready to parent** [4, 6-8], and therefore their parent identity. How prepared women felt as they approached motherhood was influenced by whether the pregnancy was planned or wanted, and whether this was their first or subsequent pregnancy. Their childhood experiences, mental wellbeing and stage of recovery were also significant.

“*I just didn’t think that I wanted kids, I didn’t know what to do with them, I didn’t know if I could love them because I didn’t know what that was.*” [7]

“*Because of the sexual dysfunction I thought first, how am I going to get pregnant and then secondly how can I be a functional parent if I have this baggage? So those two things drove me to intensely deal with that because I wanted to be a very able parent and I knew I had to do it ahead of time.*” [6]

“*I had to look at that really carefully, when I found out I was pregnant, when I was thinking about it, you know, can I be responsible for this other person? Can I give them, you know, hopefully give them the life they deserve, or to the best of my ability?*” [8]

Despite the challenges, some parents described conflicting **expectations versus reality** [7], and a vision for a **perfect or idealised family** (subtheme 1.2), based on concepts that they had understood from ‘normative discourses’ [1]. Their **identity** [2, 3, 6, 11, 22, 25] as a mother or pregnant woman was often positioned within the view of what it meant to be a good pregnant women or **good mother** (subtheme 1.2), and further defined by their abuse history. Parents described distress involved in **containing trauma and ‘being good’** [1, 11, 19], which is rooted in fear of **stigma or judgement** (subtheme 6.2) that if they were not (good), they would be viewed as “mad, bad, hysterical and overly emotional” [1]. Some women described hiding their ‘negative’ emotions or being a model patient to “*get through*” pregnancy [11].

“*I think I was conditioned right from an early age, so that programming’s always gonna be inside me and when someone’s either like threatening my personal like intimate spaces or hurting me, or telling me to do stuff and I’m like feeling threatened, I will do exactly what they say. I will be the best patient they can possibly have. I’ll be that star patient. But I’m not. I’m actually screaming inside. I’m like absolutely terrified. I’m expecting them to hurt me. I’m being good because I don’t want them to hurt me anymore*.” [25]

### 2.3 Wanting to be ‘normal’

Parents spoke about **wanting to be ‘normal’** [6, 11, 25] and described challenges with the **identity** of being a ‘trauma survivor’, which conflicted with an emerging identity as a mother and perceptions of a **perfect or idealised family** (subtheme 1.2). ‘Wanting to be normal’ links to parents’ explanations for discussing the importance of **containing trauma** (subtheme 2.2), **disclosure of abuse** and **normalising care**, discussed under *provider support* and *disclosure of abuse history* (subthemes 4.1 and 5.3).

“*I hate the idea of sometimes going round as if you've got a label on your head that says ‘I've been abused’. And you just think ‘I don’t want that’. I want people to treat me as normal.*” [25]

“*I don’t know what normal is, but you want to be normal... like everybody else.*” [11]

“*There are certain parts of me that are me because of what happened, but yet, I am a normal person. I can be a good mother, I can have relationships, I can do things and I can function. It’s not going to make me go off the deep end or do this or that. Survivors are “normal” female persons participating and functioning within society. This aligned person is the identity that seeks to accomplish a successful childbirth*.” [11]

### 2.4 Knowledge and learning about parenting

Mothers’ described their **lack of knowledge** [2, 11, 26]**,** as key to their perceived parenting ability (previously discussed) during this transitional period. Women discussed **learning about pregnancy and parenting**, or what not to do, through reading, television, observing other mothers’ interacting with their children, talking to close friends and family, self-analysis, and counselling [2, 3, 7, 15].

“*I felt like I learned how to be anything different than my parents by kind of like reading books, and kind of looking through other peoples’ lives in different little ways, like just being there in somebody else’s house, seeing a mother or grandmother just doing something that my mother would never do.*” [7]

Some women felt that their knowledge was limited due to their abusive family situation, leaving them completely unprepared and naïve about pregnancy, childbirth and the early postpartum [11, 26].

“*Because of the lack of relationship with my mother, I was afraid of the unknown. I didn’t know what to expect. I didn’t know what was happening to me. I didn’t know how to function through it, you know. Basically, I was at a loss there. I do think that had a lot to do with how I grew up*.” [11]

Expectant mothers in one study sought information about the potential impact of sexual abuse on pregnancy, labour and childbirth. These women prepared extensively during their pregnancy by reading and seeking professional advice and did not experience trauma symptoms [3].

Parents described how the above resources for **learning about pregnancy and parenting**, as well as **parent skill training** [5, 8], **access to information** [2, 27] and **reassurance** from others [11] helped them to develop parenting self-confidence and overcome some of the challenges described and achieve their aspirations of a better life for themselves and their child.

## **Theme 3. *Feeling connected*: The quality of relationships with self, baby and others has major impacts on the experiences of becoming a parent.**

This analytic theme includes descriptive subthemes around: *new experiences of love and joy*; *relationship with self/body*; *relationship with child/bonding*; *relationship with partner (and IPV)*; *relationship with family of origin*; and *other relationships and support*.

### 3.1 New experiences of love and joy

Giving birth and becoming a parent gave many mothers an overwhelming sense of **love or joy** [2, 4, 6-8, 21, 28] and a “*healthy love*” that they had not experienced before [2, 21]. Some mothers described needing to be loved, or wanting someone to love, as part of the reason they became pregnant, and that their baby’s love for them would compensate for their feelings of abandonment, inadequacy and emptiness [6, 8, 10, 21]. Cohen [21] suggests that this abuse history may make mothers more vulnerable to becoming enmeshed with their children, which can create challenges with **establishing healthy boundaries** (discussed under *coping strategies*, subtheme 7.2).

“*I guess, just basically, having a part of me that I know will not, will never leave me, that will love me for me, because it’s a part of me, she’s a part of me, I don’t wanna really say it, but, um, I guess I’ve had so many people come and go out of my life, I want someone to stay, forever, you know.*” [8]

“*When I found out I was pregnant I was on cloud nine, I was so happy. I said, I’m gonna have my little girl. I wanted a girl very badly because I wanted to give this little girl all the love and everything that I didn’t get. I wanted a little girl for me, so that when she grew up she’d be beautiful and loving, and we would be friends, a relationship I did not have with my mother. So in a way I wanted the little girl to kind of grow up as me*.” [21]

In contrast, one mother firmly stated that her parenting was not shaped by a desire to be loved, but rather by a desire to parent differently.

“*I didn’t go into it because I wanted to live vicariously through this little person, which I think to some degree my mother did, and I didn’t go into it expecting this is how I want to be loved, this is where I get my love from, and I think that that has made a huge difference in how I parent.*” [7]

Parents also described the **primacy of the baby’s needs above all else** [11].

“She relates about the labor and delivery experience, the pain and preferring one doctor over another. However, she relates no one incident sticking out because *‘I guess in my mind everything was for the well-being of my baby and that’s what I was concerned about’.*” [11]

### 3.2 Relationship with self/body

As previously discussed, many women in this review reported mixed feelings and experiences during pregnancy, birth and the early postpartum period, often related to their experiences of sexual abuse.

Many women had negative perceptions of their **body in pregnancy and birth** [2-4, 6, 11, 18, 22, 29], and how their bodies were profoundly changed because of having a child. Mothers described feeling that their body was damaged, inadequate, defective, untrustworthy or simply bad, which linked to feelings of **shame** and experiences of **triggers** (see *distress symptoms*, subtheme 7.1). Conversely, other women described a perception of their **body in pregnancy** **and birth** as powerful, capable and strong [2, 3, 6, 21, 22, 25]. These experiences related to a sense of *empowerment, choice and control* during birth (subtheme 5.1).

“*When I was pregnant I felt very proud, real proud of having a baby, I just enjoyed the whole thing. It made me feel like I am a woman. Like powerful. Yes. I really felt powerful*.” [21]

“*I didn’t wake my husband up for a couple of hours, I kind of lay around and walked around and had these contractions... it was so amazing to experience what my body was doing, because I didn’t need to do anything. I mean breathing helped... I had taken a few prenatal classes but I stopped going because it was so fear-based. It was like my body knew how to do it all by itself. It was so wonderful to just to let my body birth this baby.”* [2]

Some negative experiences of their body during pregnancy and birth were specifically related to trauma:

“*Evelyn described how everything felt “body focused” during pregnancy and how she felt uncomfortable and unsafe in her body and not in control of how it was going to change: “I just don’t ever I didn’t ever feel... really that... safe and comfortable in myself*.” [22]

Parents in several studies described the **baby as feeling foreign and invading their body** [2, 11, 25], which were related to feelings of a lack of control.

“*…When I thought about the baby being inside me, I would just get sick. Like I had to come to terms in myself and allow that thing to be in there, like giving permission. It sounds really weird and convoluted, but that was something that I went through very strongly and I think that the reason I knew that they were related is that I had so many nightmares during those times... I knew inside me that the baby was a baby and mine, but just where it was habituating was the issue for me. Just being in that private area.*” [11]

Negative body experiences also included **co-morbidity or illnesses** **in pregnancy or physical symptoms** [3, 4, 29].

Some women questioned whether they could become pregnant because of their **sexual traumatisation** [18, 22] and pain or reproductive dysfunction [2, 6]. Some women with a history of sexual abuse also spoke about their difficulties in having sexual intercourse and a fear of intimacy [3, 6, 18, 22].

“*I found it hard to sleep with him to get pregnant I found that quite um traumatic and the thoughts of it to start with I was sort of going through how I could get pregnant without having to have sex... I sort of forced myself in the end thinking that’s this is the only way.*” [22]

Some women who had been struggling with **fertility issues** [2, 3, 6-8] were surprised they had conceived. Some considered their bodies as “*spoiled*” by the abuse and reported a **conflict between being ‘spoiled’ versus wanting to be ‘pure’** [2, 8]. This links to concepts about a ‘**perfect’ or idealised family** (subtheme 1.2).

“*...and I never did once feel uncomfortable, but then I look back and I think, did I block it because I wanted this to be a good experience? And, I loved being a mom and I loved being pregnant. Maybe I didn’t allow myself to feel negative thoughts, I just wonder now that I look back if I just didn’t want anything to spoil it ‘cause I had felt like my life up to that point had been spoiled, tarnished, and I didn’t want anything to (voice breaks) harm my memory*.” [11]

### 3.3 Relationship with child and bonding

Parents described mixed and positive experiences of **bonding** [6, 7, 26] as well as challenges with **early attachment** [2, 6, 7, 11, 17, 18, 26] with their child. This linked with experiences of postnatal depression, **negative self-belief**, perceptions of a **perfect or idealised family** and **fear of repeating the past** (see subthemes 7.1, 1.2 and 1.3, respectively), which also link to concerns about **baby gender** (outlined below).

“*The first thing I felt when they put him on my stomach, I thought to myself, I feel clean, you know like all that bad stuff was washed away.*” [26]

“*When my son was born I was not able to bond... I just pushed him over to his father. I didn’t want anything to do him, but I wanted to be sure that he was safe and protected, in good hands... but it needed to be somebody else... not me. Because if it was me, then he... he could possibly be a pedophile, he could be like my father. That is why I told myself through my pregnancy that I was carrying a girl. There was no room in my mind for the baby to be a boy, and so when James came out, he had no name... My husband had to name him because I... I couldn’t even go there.”* [2]

Parents described positive experiences [22, 24] and challenges [2, 6, 7, 18, 21, 22, 24, 25, 27] of **breastfeeding.** Survivors of child sexual abuse also discussed positive experiences [6, 24] in relation to **bonding or attachment through breastfeeding,** however some parents noted intense feelings of discomfort [13]**.**

“*It’s the love. It’s the giving of my milk to him and sharing with him. I am the only one that can do that for him and it is so strong that love. To have this little baby attached to you makes me feel a really strong connection*.” [24]

“Having felt trapped during pregnancy, Sam had the same experience breastfeeding. She felt that: *...it’s like it’s happening again because you are being controlled by another person. And even though I really tried not to feel like that, it happened every single time um that I tried breast feeding. I felt that immense feeling of being controlled by someone else*.” [25]

For some, they felt their ability to breastfeed reflected on their ability as a parent:

“*She wouldn’t breastfeed, it was all what am I doing wrong, what is so wrong with me, why can’t she latch on, why can’t she attach to me, and that was a pattern that was repeated that was my mother and my dynamic.*” [7]

Some women discussed the effect **dissociation** had on their breastfeeding experience [27].

“*I think a part of my experience was that I didn’t really have a body. I was a head with legs. Or with feet. I was just kind of a walking turtle... I ignored everything in between... I remember the breastfeeding. I remember the frustration. But I can’t say I remember any feeling from it - physical sensation. I didn’t register any body sensation anyway. I was just - the breasts were functional for that time.*” [27]

The **baby gender** [9, 21] was a factor impacting on the **parent-child relationship** [21, 29].

“*When I was pregnant I always thought, well how will I feel if I had a girl. I really feared it. I feared that my husband would play with a girl life he plays with a boy, like he’d play with any other kid, but that I am going to feel like, is he playing correctly. I trust my husband but I don’t trust myself, and I know I would read things into it that aren’t there. Sometimes I really felt I could get crazy with that, with thoughts popping in and out, and I kind of dreaded it, having a girl, cause I didn’t want to drudge up all those other feelings, and I wished for a boy.*” [21]

“*I worried a lot. I’ve always been so anti-abortion, but when I found out it was going to be a girl, I wanted to give her away or just not have her. I was so scared I wouldn’t be able to protect her and that something would happen to her. It made it hard. It made it hard a lot, because I thought about [the sexual trauma] a lot when I was pregnant.*” [9]

### 3.4 Relationship with partner (including IPV)

Mothers described a **positive** **relationship with their partner** [2, 8-10, 15] and considered the **partner support** [3, 8] as critical during the parenting transition. However, **challenging partner relationships** were also frequently reported [1, 2, 4, 6-10, 17].

“*I met my baby’s dad and we knew instantly that we loved each other and that we wanted to be with each other. We talked about what we wanted out of life and decided that we both wanted to be parents. I took into account his family and how his parents are... how his family lived... I thought, ‘You are going to be a good dad’. When I decided to have a baby with him, it was me wanting to share my love with him. It was my way of saying, ‘I love you and I know that you are a good person’. It was a whole new beginning.*” [2]

“*Like he knows what I’ve been through, but it’s just confusing for him because, ‘Why are you acting like this now?*’ *And like I said, he’s already sceptical about this baby. And now I’m pushing him away more. So our relationship is going through hell in a hand basket.*” [1]

Many women described break-down of relationships during pregnancy, which study authors reflected could impact on the mothers expressing their wish for their child to look like them [4]. Several women described experiences of **IPV in pregnancy**, which can compound the complex trauma experience [14, 30]. However not all women wanted help from maternity care providers to address IPV [30]. One parent also reported challenges with their **partners’ family** [4].

“*He [the father of the baby] heard that I was pregnant and wanted to beat me up. He beat me so that I would lose the child but I didn’t lose it*.” [14]

### 3.5 Relationship with family of origin

Parents described mixed experiences in their relationships with their **family of origin** [3, 4, 6-8, 10, 14, 21] during pregnancy, birth and early postpartum. For some, becoming pregnant was a time for a ‘fresh start’ with this relationship, setting new boundaries and ‘letting go’ of the past [3], receiving practical and emotional support [4, 8, 14], and having an opportunity to reconnect and heal [8, 10].

“*Everything is changed since I’ve had a baby. For my family, now they all have to treat me as a responsible person—my entire family seeing me become a mother definitely has changed who they think I am in their minds. That’s the biggest way my relationships have changed; they have to re-evaluate who they think I am, and my relationship with them changes accordingly, because I haven’t really changed that much, I’m still the same person I was before I got pregnant and while I was pregnant, except I’ve had to start being more healthy, but essentially I’m the same person, just minus and plus a few things. But motherhood has meant a major change of status in their eyes; I had ‘irresponsible drug addict delinquent status’, and I cannot be that anymore*.” [8]

Other parents described challenges in their relationships with their **family of origin** [3, 6-8, 21], including learning to trust [3] and ongoing conflict [8]. Some of these challenges seemed to be associated specifically with adolescent pregnancy [8]. Forming a maternal identity was associated with internal conflicts as women set new boundaries with their families and with their child [2, 3, 6, 22].

“*And thev know, thev are verv clear, both of them, that they are not going to mess with me ever again. That there are definitely boundaries. There are definitely things that I'm willing to accept from them and things that I'm not willing to accept. And that behavior, I am not willing to accept.*” [3]

### 3.6 Other relationships and support

Parents described the importance of **supportive relationships** [2, 4, 6] and role models during pregnancy and early postpartum as well as having **support people** **during birth** [4, 28]. The **need for** **support** [6, 11, 17] was highlighted as an important factor in healing during this transition, with types of support detailed in theme 7.

“Bonnie’s choice to keep the baby was influenced by her support system: People would have been understanding and supportive either way, which helped a great deal because I don’t think I could have made that decision knowing that I didn’t have any support. That was very important. Often participants reported that having a support system in place was of critical importance. Carol asserted: *“He [husband] was beside me every step of the way... even when I pretended that I didn’t need him, he stayed with me. I don’t think I could have done it without him.” Carrie’s support system was a friend: I had a really special girlfriend that I could share everything with... so she was a life-saver when it came to my pregnancy. I don’t know how many times I called her crying my eyes out... she never judged me or said I was bad, she just came.*” [6]

**Lack of support during pregnancy** [2, 6, 9, 12, 17] and **early postpartum** [6, 26] was a particular challenge frequently highlighted by parents, with some describing intense feelings of isolation.

“*I didn’t have a lot of friends and I didn’t have a lot of support... I was alone with my son and I was sick. That was really tough; those nine months were really, really hard for me*.” [2]

This was particularly important for parents who may have had difficulty developing supportive relationships with others, including **friends** [6]. It raised conflicts and challenges for some parents, between their need for support and the instinct to push people away, or the experience of having trouble **letting people in** (see subtheme 4.1).

“*Whether that relationship was a close friend, a therapist, a doctor, a massage therapist, a therapy group or a spiritual community, the importance of connection and relationships was evident throughout the interviews. For these survivors of sexual abuse, being able to talk with someone about what seems unspeakable without that person "running out of the room screaming" as Alice said, could be the first concrete step towards healing*.” [3]

“*She rubbed my back during contractions and it was just what I needed. I didn’t even need to tell her to do it... she just knew that it was what I needed. I felt really safe having her there since she knows me and loves me no matter what*.” [6]

“Dee told of all her relatives who were there during her labor, *‘I had my boyfriend there, my mother there, and my boyfriend’s mother was there in the delivery room. My aunt came in while I was pushing, my sister came in, and my boyfriend’s sister came in with her friend.’* When asked specifically who provided the most support, however, she said, *‘Mostly my mother. I felt more comforted, you know, like she was gonna take care of me. And it was great cause I was holding onto both her hands.’*” [10]

## **Theme 4. *Compassionate care*: Kindness, empathy and sensitivity enables parents to build trust and feel valued and cared for.**

This analytic theme incorporated descriptive subthemes of: *provider support, communication and relationships*; *trauma-informed care and factors which factors which foster safety and enable care*; and *experiences of care during birth.*

### 4.1 Provider support, communication and relationships

Parents described positive **perinatal care experiences** [14], with **supportive providers** [2, 5, 18] as key to influencing their experience of perinatal care.

“*People who believe them. People who validate [what] they’re experiencing; who don’t compare them to other people who... help them take responsibility for their role but... don’t blame them for it; I think like, ya know, try to relate to your population*.” [5]

Some parents referred to care during pregnancy and birth as being ‘**normalising’** [22], which links to descriptive subtheme of *wanting to be normal* (subtheme 2.3).

“*It was very different to become this normal person going to normal hospital appointments um that normal people would do I know this is all very normal but um, it was it was quite a nice experience thinking I’m doing things that um people will do without mental health problems.*” [22]

However, parents described how it was difficult to **let others in** [2] and **trust in care providers** [2, 11, 18, 23, 25, 26, 30], which links to a perception of the *world is unsafe* (subtheme 6.1).

“*I would be very uncomfortable with a male doing an examination; simply because I was abused by a male and I don’t trust any of them, especially in the 30- to 40-year-old age group. If I had to hand her over for surgery, it would be incredibly traumatizing, I mean it’d be bad enough being a mother and having to hand her child over for surgery but having been a child who got abused and having my trust violated, it would be extra hard... to hand over another innocent child and trust... I trust you not to hurt her... especially if it was a male in that category of men I distrust... it is just difﬁcult*.” [23]

Parents described many challenges related to **lack of care, empathy or understanding** [2, 18, 25], **inadequate care** [2], and **poor provider communication** [2, 6, 11, 18, 22].

“*I was exhausted after the birth... I said, 'I feel ever so weepy’. ‘Oh, don't start that off!’ she said, 'I'm not going to be 5 minutes!’ I mean, she was a cow. She really was awful!*” [18]

These negative experiences related to parents’ feelings of **being disregarded or depersonalised** [2, 6, 11, 18, 23, 25]. These experiences reflect the importance of ‘compassionate care’, with women describing concerning experiences of lack of care and respect for their choices, particularly in light of the trauma experience of women.

“*...it was when doctors sutured you up... it was an SHO [senior house officer] who'd obviously been dragged out of bed… He didn't look at me once, didn't, didn't sort of get eye contact whatsoever... and I felt every single stitch he put in, every single, and I cried all the way through.*” [18]

“*I’m there with my mother, my doula and my husband, a nurse and a doctor in the room when they finally decided to do the C-Section. The nurse rips my gown up goes over to get the razor and the little thing of water and comes over and starts shaving me and I’m like, oh, hi mom and hi my husband, how uncomfortable for you both to be standing here and looking at that spot. I think that was like the only part that was to me kind of amusing, oh wow, they really just don’t care do they? They just whipped up my gown and just exposed me to my mom and my husband and my doula without even saying, ‘Okay, now we’re going to shave you.’ There’s never any words that make me uncomfortable because I think, god, at least ask before you throw that thing up over my head and shave my pubic area! No friends, no family, no one else gets to see down there. And so when they flipped the gown up and didn’t even tell me what they were doing and I’m trying to pull it down because I don’t know what she’s doing.*” [11]

“*I didn’t want to be strapped down. I didn’t want anything in my arms during my first pregnancy. I didn’t want to be on a table. I wanted to walk around. And I had this nurse from hell. She was just so tied into what you have to do and what the rules are. And, oh, she got me in that bed and she just put those needles on... in me and you’re gonna be in bed. And I just stared to panic all over again. And I kept telling her I want, I just want, I don’t want the monitors on me. I want to be up. I want to be able to get up to go to the bathroom and then they put the catheter... and you’re there... it’s like being a prisoner all over again*.” [11]

For some, these experiences led to preconception, pregnancy and childbirth **care being reminiscent of abuse** [22, 24, 25, 31] which was linked to **re-experiencing or being triggered in care setting** [2, 6, 18, 23, 25, 27, 31, 32]**.** Some of this relates to experiences of severe perineal and abdominal pain, and other mothers describe similarities due to the constraints and control during labour and birth. This included descriptions of care as **invasive and violating** [2, 6, 18, 23, 25, 26]. Coles [23] argues that, due to the challenges of **disclosure of abuse** (subtheme 5.3), perinatal care providers should adopt ‘universal precautions’ and provide sensitive trauma-informed care for all mothers.

“A particularly recurrent theme in Sally's interview was the manner in which she felt dehumanised by carers' -lack of compassion, which she referred to as the 'no care attitude'. This was strongly reminiscent of the attitude of her abuser, and clearly caused her a good deal of distress. Her words strongly resemble Claire's comment about her carers*: ‘...with him [stepfather] there was no care for who you were... He always reckoned he loved you but there was no care for who you were, which is why when there's like the midwives and the doctors that are just 'Oh, I've got to do my job' sort of attitude - it's that 'no care' the same as what he gave’.*” [18]

“Caitlyn clearly describes her feelings and distress during her repeated pelvic examinations, comparing her body to a toilet door, something that was barely noticed as the hospital staff passed through during the examinations.” [23]

“*I think I was angry about how I was treated at the hospital and it took me awhile to think through why I felt so uncomfortable in the hospital. It wasn’t until Emma was four or five months old that I finally realized, ‘They had no right to touch my body like that! They had no right to treat me like that!’* ” [2]

**Effective communication in the care setting** was identified by many parents in this review as critical to improving their perinatal care experience [3, 5, 18, 24, 30, 32, 33]. This was considered critical for enabling safety and **disclosure of abuse** (subtheme 5.3) [33]. Some parents provided examples where they were very directive about the care they needed during pregnancy and birth, but this required a fair amount of individual agency. Positive experiences of providers advocating on behalf of parents were also described [3].

“Jane highlighted the need for health practitioners to introduce themselves, give explanations, and ask permission when she described the problems she encountered with control being taken from her at home by the nurse who *‘waltzed into her house’,* labelled her with a mental health problem, and then proceeded to examine her breasts without permission*.*” [24]

“*I think the nature of the person, like the nurse I saw last week before my scan. She was talking to me and asking me how did I like living here and interesting herself in me as a person before she did any of the examination and explaining what she was going to do and why she did it. So, I suppose, being acknowledged that I'm a person there, rather than an object on a conveyor belt of vaginas that she's looking at*.” [18]

This included **respect, empathy and understanding** [6, 18], and providing **personalised individualised care** [11, 18, 24]. Parents described the importance of asking people for permission before touching them, being gentle and engaging with people as individuals, with compassion and understanding [18].

“*... and she [midwife] said, 'may I examine you?' And I let her examine me as well... She asked my permission first and said, 'this is what I can do' and I gave her my permission, and she went ahead and did that. She was very, very gentle, she was lovely.*” [18]

“*Like you could just feel like her attitude was just common sense, down to earth, trust your body and also I’m not going to do anything that you don’t want me to do, you tell me about how this has happened, where do you feel it and you could just feel that the way she was talking to me she was just totally respectful and she was like you are the expert here is some ways... you know, that kind of feeling. She made me feel safe... she made me feel like I’m not stupid here, she’s not just the expert here telling me don’t do this, do that, that kind of thing... but this is your body and I know that we are talking about your body and that the kind of thing that was great. Yeah, this is my body and it has been traumatized*.” [6]

“*As soon as he walked into the room there was eye contact made and he did a salutation, ‘how are you doing today’, that type of thing. He didn’t come in and fidget with paper or read your chart. He came in and looked directly at me and that made a world of difference*.” [11]

**Lack of continuity of care** and needing to repeat their story was noted as distressing by some parents [5]. **Continuity of care** [6, 18, 23, 26, 32, 34] was emphasized as an effective strategy for minimising these issues, and building relationships and trust with providers, which links to theme 6.

“*Developing a really good relationship, like when you trust the GP and where you see the same GP, that’s really good, being able to go overtime with a GP if you’re in crisis or a bit distressed... not having them constantly sort of check their clock and things. A GP who will sneak you into the queue because they’ve noticed that you’re crying, they’re concerned that you’re suicidal so they let you jump ahead of ten other people. Things like that, just going beyond the call... just little things like that, they are the things that you notice.*” [23]

### 4.2 Trauma-informed care and factors which foster safety and enable care

Parents discussed other challenges in relation to **postpartum access to care** [23], which relate to **stigma** (subtheme 6.2), **avoidance of care** (subtheme 7.1) and **others’ expectations** [6].

“*Basically I just didn’t go to see a doctor or take prenatal classes or anything like that. I really didn’t want to get all caught up in the pregnancy thing... I had enough going on already and I didn’t want to have to be all nice and cheery and tell everyone that everything was wonderful, because it wasn’t wonderful. I hated being pregnant but that’s just not something you say. People would think that I was a bad mom and I already felt that inside... I didn’t need to feel it from others.*” [6]

“Melissa does not return to her physician after giving birth nor regularly attends prenatal care. She states, *‘Because I get scared. I don’t know why. But, I never go back for ‘em. Like after I have my kids they say to come back for a check up. I never go back.’* ” [11]

“Accessing services can be difﬁcult for new mothers with young babies. The participants raised issues of getting organized with the new baby fed, clean, and at the doctor’s or nurse’s appointment on time. The participants went on to describe how when things were deteriorating and difﬁcult, it became increasingly difﬁcult to access services and support outside the home*.*” [23]

However, parents described many positive experiences and examples of ‘**trauma-informed’** **perinatal care** [2, 3, 5, 6, 11, 22, 23, 25, 27, 30].

“Penny described the importance of professionals understanding the impact of trauma to improve the care they provide to survivors*: ‘By understanding the long-term effects of trauma, understanding that there’s different types of trauma, and understanding that their [the health practitioner’s] body shape, their build, their characteristics might be more or less stressful to a person and that it’s nothing personal. Understanding that somebody who’s been traumatized 10 years ago, 20 years ago, 50 years ago may have no relevance to how far they’ve progressed. Asking their patients when they are comfortable, and asking their patients what they need. Asking questions early is more useful than when we are in crisis*.” [23]

Key elements that helped with fostering positive relationships and safety to counteract trauma-related shame and mistrust in others included **positive strengths-based approaches** [5], **continuity of care** (previously discussed), **multi-disciplinary care and collaboration**, including family-centred care [5] and **improving professional contact opportunities** [23] to ensure the frequent contacts with providers during the perinatal period are used to their full potential. Muzik et al [5] argued for the need for ‘hope affirming practices’ that supported parents to achieve their hopes and dreams for the future with their new child.

“Mothers indicated that the word trauma emphasized injury and damage rather than healing... Healing is a journey between ambivalence and hope. Services for trauma-exposed mothers should acknowledge the normal ambivalence surrounding seeking help, but should be designed around hope-afﬁrming practices. Women in our study identiﬁed several hope-promoting features, such as peer support, group interventions, and safe, comfortable, service integrated delivery sites. Finally, children and the motherhood role are chief motivators for hope, and therefore, child-friendly services are more likely to appeal to these families. Programs modelled upon these suggestions may be a powerful motivator to seek care, as well as a crucial anchor to engage in treatment long-term.” [5]

Two studies outlined a comprehensive range of factors included in trauma-informed perinatal care. Muzik presented survey findings to suggest that “many mothers (94%) welcomed the idea of access to a range of multi-disciplinary, holistic, healing and well-being services that meet their needs as trauma survivors, as women, and, particularly as parents” and outlined 10 important aspects for these services [5]. Seng presented a similar set of guidelines for providing trauma-informed perinatal care, which were considered relevant for all women at different stages of the healing continuum [30].

“Being aware of how a woman might be affected, even if she is triggered and then cannot articulate her concerns initially, can be of critical importance in soothing rather than exacerbating a posttraumatic reaction. This woman’s experience with preterm contractions illustrates what a difference this awareness on the part of the provider can make*: ‘…and by this time I could tell I was teetering on the brink of having everything rush back. And I was visibly shaking, my voice was very shaky, I sounded like a little kid when I talked. And she, the midwife, really picked right up on it, and she said before she examined me…she sat down and she explained exactly what was going on…she said the same thing six different ways until I physically…you could physically watch me relax. And then she said, ‘Why don’t we just take a peek and see what’s going on?’* ” [30]

### 4.3 Experiences of care during birth and breastfeeding

Parents described mixed positive experiences [2-4, 18] and challenges [2, 4, 22, 26] related to **birth**.

“*They had to tell me to let her go so that they could weigh her and everything. They was like, ‘You’ve been holding her for an hour now’. You know, but my first reaction was to put her on my chest... I was just like, ‘My baby!’ It was like, I don’t know, it was like a dream... I was just so happy to see her face.*” [4]

“*It was like I didn’t like the feeling of labor or any of that... I was like, staying in the hospital and all that, ugh I hated it!*” [4]

Positive experiences link to parents’ descriptions of the **healing process of birth** [3, 6], with birth seen as a transformative experience and a “turning point in their perceptions of their abilities and the possibilities for their lives.” [3]

“*It was just the most wonderful, magical experience of my whole life. I feel like things changed for me right there.*” [6]

**Support people** [4, 10] (discussed previously under ‘relationships and support’) were essential elements of a positive birth experience. However, some mothers described challenges with having a **birth partner** [2, 11, 18, 26], and feeling alone. These challenges included restricted visiting hours, [18], partners being absent and family not being supportive [4].

“*They were telling me what to do, and what I couldn't do and... I can remember saying that I felt you know, the pains were really bad because I went to the hospital and they sent my husband home because I was obviously in early labour and um... and I was completely on my own through the night, wandering around corridors... trying to keep quiet because people were trying to sleep*.” [18]

Positive birth experiences were also associated with positive feelings about the **body during birth**, **bonding**, and **empowerment or control** (subthemes 3.2 and 5.1). Several women talked about their **hopes for the subsequent birth** [2, 3], reflecting on previous experiences, and aiming for greater control during the process. Parents also experienced challenges during birth related to **traumatic birth experiences** [2, 4, 6], **stillbirth** [2] **and pregnancy loss** [2].

Many women also described **breastfeeding challenges in the care setting** [2, 6, 18, 24, 27, 34], particularly those who had experienced previous sexual abuse.

“*When I was breastfeeding, I was all exposed but they [nurses] didn’t seem to care. They were like, ‘we’ve got to get this baby to eat so we’re going to do whatever we have to, we need to make sure the baby eats.’ And they didn’t seem to care about the fact that they were pushing my body around*.” [6]

## **Theme 5. *Empowerment*: Control, choice and ‘having a voice’ are critical to fostering safety.**

This analytic theme incorporates descriptive subthemes of: *empowerment, choice and control; having a voice; and disclosure of abuse history.*

### 5.1 Empowerment, control and choice

**Lack of control** and feeling overwhelmed during pregnancy and birth was a strong theme discussed by child abuse survivors [2, 6, 11, 17, 18, 22, 23, 25, 26, 29, 31]. This included women feeling like they were passive rather than active participants in the birth process, lack of privacy and the ‘medicalisation’ of birth. The authors of one study [17] described how “Those who identified some sense of control were more positive and hopeful about their pregnancy. Those who felt they had little control over their circumstances experienced high levels of stress and used disassociation to help them cope with their fear. Two women felt they could not cope and required hospitalization”.

“*I felt totally disempowered. When I walked out, I was crying uncontrollably and yelling at the children. All these things that are a chain reaction to... her making me feel bad. She made me feel bad about myself as a mother, and about my children and their behavior. I think, as a patient, you have to start exploring different areas to actually connect them with your past abuse. Sometimes you do things you don’t even realize you do because of your past. It’s just the way you are used to dealing with people. Like the doctors and the health nurse, I felt like I couldn’t really say too much about it because I couldn’t just stand up and say no.*” [23]

“*I knew I was going to have to deliver this baby... and I knew there were going to be more examinations and things being taken out of my control again, because I didn't feel strong enough to say 'No, I don't I want you to do this', or explain the reasons why I would be behaving in certain ways*.” [18]

“Many participants associated touch with vulnerability and lack of choice and control. For example, Cathy states: *“I felt like I had no choice, it’s like whomever came in had a right to touch me. It’s a pretty vulnerable place to be in.”* Suzanna reports similar feelings of vulnerability*: God I was so vulnerable when she was coming... with all the people touching me and stuff... like being in all that pain and not being able to control the pain and being at the mercy of everybody else. I didn’t like them touching me so I just went away... like in my head, I just went away and watched from above*.” [6]

“*I couldn’t just like just walk away from the situation... I just kept on feeling like I was trapped with him [son]... I just felt like I’m going to be really really vulnerable, um, and kept having this image of me like on the bed, you know I can’t get out that situation I can’t... I have no control over it.*” [18, 22]

These feelings of lack of control were linked to feelings of **being disregarded or depersonalised** (subtheme 4.1), **vulnerability** [11, 25], **shame/humiliation** [18] and fear (discussed in theme 6). Some parents talked about a sense of inadequacy and childhood experiences of being abused and sometimes being not believed by people in positions of **authority** [2, 6, 18, 21, 23, 25-27], which impacted on their current experiences of perinatal care. and **care provider gender** were also highlighted as significant factors impacting on relationships with care providers and a sense of control. And while most mothers said they felt less vulnerable if the care provider gender was female [5, 6, 11, 18, 25, 30, 32], this was not universal, with one women expressing a sense of relief at ‘not having to explain anything to a male’. [11]

Unsurprisingly, parents emphasized **empowerment, choice and control** as a critical element of positive birth experiences [2, 3, 6, 26], and for helping to improve perinatal care experiences [3, 6, 11, 17, 18, 25, 26, 30, 32, 35].

“*Labor was probably my biggest success. I was present all of the time, natural, complete, and supported... certainly aware of the pain and the changes and all of that, but it was very empowering because of that ‘hey, I can do this’ feeling. It is maybe the first thing that I can do completely, be in charge of and that really was a drive for me. It turned out to be positive that I could focus so clearly on that. I guess my fear before was that I would feel overwhelmed that I would feel violated, that I would feel this was being done to me and out of control. Those were my fears around giving birth so by actively working around those issues, that I could turn them around and that I was able to do that. I could feel that I was being violated somehow but surrounding myself with a support network and realizing that they were all there honoring what I was doing it just helped*.” [6]

“*I was really excited… but at the same time, I was pretty frightened because I knew, you know, I wasn’t sure if all the exams and all that stuff was gonna trigger a lot of things. So we decided to go with a midwife, and we started off at one place and then went to another place… and [the midwife] assured us that she had dealt with this before… and we just really laid down… ‘This is what we want. This is what we’re gonna do. Can you provide this for us?’ And they were very receptive, and things progressed actually really well.*” [30]

Some parents described how **having a birth plan** [3, 6] or **home birth** [18] helped them to achieve more control. One parent described developing a birth plan with a trauma specialist [6].

“*I think the birth plan made it really positive and we discussed it with my doctor... about certain issues in the birth plan and then I would go over it myself and then talk with my mom and then during the prenatal classes... I loved my prenatal class, I thought it was great. I talked about it at prenatal class and this class was taught by a doula, which was great*.” [6]

“*I didn't want to be part of the conveyor belt system, so I decided I was going to have a home birth.*” [18]

### 5.2 Having a voice

Parents described challenges with **talking about trauma** [3, 25] and **birth experiences** [6] and the anger, frustration, resentment and suppressed feelings that could build from not talking.

“*Because I feel that I sort of retracted in to myself, it meant that I was easier to be ignored yeah? So then I feel invisible and that makes me feel really, really, really angry because I'm thinking ‘well, I'm here, you know, everyone else is getting a better deal than me, I'm still here but no one's noticing me.* [25]

“*I laughed when visitors came and I smiled and I put the right face on. But inside… (sniffs, four second silence). Inside I was – just silently screaming.*” [25]

**Asking about trauma** [2, 30, 33] (which links to disclosure of abuse, subtheme 5.3), **expressing trauma** [2, 3, 6, 9] and **communication** [7] were all seen as helpful by parents. However, there was no ‘one size fits all’ approach and there was a lot of variation in both the readiness and timing of parents for ‘telling their story’, and the way they wanted to do this.[3] White [33] outlined five themes for safe trauma enquiry: “(1) a clear definition of trauma, (2) clear purpose for inquiry, (3) reassurance that inquiry was routine, (4) confidentiality, and (5) mention of helpful resources other than psychiatric therapy.”

“Several of the women in this study described feeling empowered by the opportunity to share their experiences. Thus, although reflecting upon their childbearing experiences was difficult, it was also healing*. Lee: ‘It’s like I can talk about the past, the abuse and all that. I don’t feel anything but the truth... just numb... but when I talk about my daughter it just brings it all back. It’s so hard. This is good for me though... I think it helps me to heal’. Lisa: ‘It’s all starting to connect for me now... you know, the memories about my abuse and being a mom. It’s beginning to make sense for me why I am finding it so tough sometimes. It’s like I don’t know what’s normal or not and what’s right or wrong. I have always felt like I am half a person and now I have to connect with the other parts... the dark places that I have been unwilling to go, at least consciously. Being a mom has made me go there, because I have to... but it hasn’t been easy. I think talking about it is worthwhile though*.” [6]

“*I guess not keeping it bottled up inside of me probably made, played a major part in my life because I felt that whenever I did keep it bottled up, I felt like I’d went through a lot; and now that I’ve talked, I’m more open about talking about it, I feel it has changed my personality a lot. And, then like, I don’t feel that pressure there anymore as much as I did before when I didn’t say anything... I kept it bottled up for a long time.*” [9]

“Communication skills directly influenced the survivors experience by either leaving them feeling alone and without support*, ‘When I do try to talk to someone, I realize that I can’t talk to anyone. I have these questions, my sister doesn’t know either, and we don’t know where to go with the questions”* or understood and supported *“I remember when I just couldn’t get anywhere with my mom, or couldn’t, I felt like I couldn’t talk to anybody about anything, that surprisingly enough my dad was there”.* One participant expressed finding a way to communicate through writing. She stated that she *‘always preferred writing then talking’.* Finding a way to effectively communicate was indicated as being an important factor during the transition to parenthood because it influenced their ability to express, and seek assistance in meeting, their needs*.*” [7]

### 5.3 Disclosure of abuse history

**Disclosure of abuse** was perceived as positive in terms of enabling support and more sensitive care, as well as advocacy for women [3, 6]. But there were also many challenges to **disclosure of abuse** [2, 3, 6, 11, 18, 22, 25, 30], including not being asked [22] and **impact on family** [18], with a high degree of variability between parents. This relates to previous descriptions of women feeling they are invisible and silent while ‘screaming inside’. This silence creates challenges for perinatal care providers and could lead to parents feeling they are being disregarded and overlooked. Hence recommendations for ‘universal precautions’ [5] in the context of parents childhood experiences of maltreatment are salient.

“*Pregnancy is a time for purity. I want my pregnancy to be pure and not tainted with memories of a dirty past... even though it wasn’t my fault. I don’t want to be talking about horrible stuff like what happened to me as a child and I certainly don’t want to feel like I am going to do the same to my child. I just don’t think pregnancy is a good time to talk about it. I just didn’t even want to think about it then*.” [6]

These challenges were related to **fear** and **trust in care providers** (discussed later), **fear of losing the child** [22, 25] and **fear of not being believed** [22].

“Ella was also silenced, not feeling able to ask questions or disclose her abuse for fear of not being believed*: ‘You think no-one’s going to believe you’.* Ella was never asked whether she had experienced abuse and felt fearful that if she told someone then her children would be taken away*: ‘I never came forward because I thought they were going to take my baby away’.*” [22]

“*Unless we can take away this awful cliché that the abused become the abusers. Then, I don't know if anybody will ever be really free of fear enough to talk... [I was] terriﬁed that at some point, somebody was gonna ﬁnd out, that I'd been abused and that they were going to put this label on me and watch me. And look out for the signs that I was somehow dodgy.*” [25]

Other challenges parents raised in disclosing abuse included **not wanting to burden others** [providers] with abuse stories [25] and **wanting to appear normal** to providers [6, 25] and minimise the impact on themselves, also discussed above under *wanting to be normal* (subtheme 2.3).

“Women with traumatic childhood histories have spent their lives enveloped in secrecy and shame, and exposing the “secret” is not only profoundly unsettling but also risks having demands and judgments placed on them. Labelling signified being different, and this was perceived as threatening by these women, as they had worked throughout their lives to be accepted.” [6]

## **Theme 6. *Creating safety*: Parents perceive the ‘world as unsafe’ and use conscious strategies to build safe places and relationships to protect themselves and their baby.**

This analytic theme incorporates descriptive subthemes of: *the world is unsafe and strategies to protect themselves and their baby*; and *the external world around them*

### 6.1 The world is unsafe and strategies to protect themselves and their baby

Concerns about safety were a major challenge raised by parents in this review. Challenges were raised in discussions around **safety in** **disclosing their abuse history** [11, 30] and **safety or protecting themselves and their baby** [1, 3, 6, 9, 11, 21, 23]. These perceptions of the world as unsafe could lead to parents being overprotective of their child in **monitoring safety and** **hypervigilance** [2, 11, 23] and challenges with regards to **establishing healthy boundaries** (subtheme 7.2).

“*When she gave me the initial, you know, the history form… when I saw ‘Were you abused?’ I said no. There was no way I was going to tell her*... *At the very end of my seeing her…I needed to tell her so that if I were to get stumped in the middle of delivering [the baby] she would understand why’*. So telling or disclosing the history of abuse was not something that was performed easily. Instead, several encounters were often required before a sense of trust and safety was developed with the health care provider.” [30]

“*I realize that she is going to hurt. But God forbid that she should go through what I went through. I’m hoping that if I keep an eye on her, and watch the people that are around her, that that will be enough. I’m hoping that if I do what I’m supposed to do, that God will protect her from that. She can have other horrors, but just not, not that.*” [1]

“For Liz, it was wondering what people are thinking of doing when her baby’s nappy (diaper) was off*: ‘Just more watching people change her, being more curious, and just wondering what people are thinking when they were changing her and what are they going to do—just not letting them change her away from me either—I like to hover’.*” [23]

“*For the first few weeks, I was really anxious about, the mechanics of having a baby. For instance, I didn’t want to touch her genitalia because I wasn’t sure if it was sexual abuse. It was really hard for me to learn that washing her and changing her were okay; not just for me to do it but for her father. Even now, if he’s feeding her and she starts to cry a little bit, I have to go up and make sure that he’s not touching her... and I trust my baby’s father with my life*.” [2]

“*When I am alone in the room, I’m looking for signs. I think because my GP is a male and, of course, he has to touch me in very private places. That was hard; at the very start I was looking for signs, like when I was on the bed [examination couch], maybe his hip would touch my arm or his tummy would touch my arm and I would go... oh... what are you doing? Then I would realize now he’s not doing it abusively... he’s just trying to do his job. Things like that still affect you strongly, so you know that you’re on edge worrying. I don’t think we would ever get over it. I think we just need to learn how to live with it and that’s it.*” [23]

For some, protecting their baby involved managing relationships with their **family of origin** (subtheme 3.5):

“*We are going through the process - do we send a birth announcement to my parents. We would rather not. We are going to find out the gender and we are going to tell some of the relatives and I don't even want my parents to even know the gender or the name. But how do I prevent that? How do we do it without cutting myself off from other people in my family that really matter that I think it would benefit my children to have a relationship with?*” [3]

And some women even gave their children up for adoption in an effort to protect them from their own family:

“*The baby was a girl and I knew that I couldn’t take that baby home… because she was a girl, because she wouldn’t be safe because my daddy lived at home and he was on to my sister at this time. I just knew that I couldn’t bring that baby home... Most of us women gave up our kids for adoption... there was no support in the family, you were just fucked up for the rest of your life just because you were a dirty little slut*.” [6]

These concerns about safety were strongly related to challenges in **trusting others** [9, 18, 21], including **trust in care providers** and the need for **empowerment, choice and control** (subthemes 4.1 and 5.1).

“*I was scared of other people… many people thought I was shy, but it really was not that I was shy; I just did not trust anyone, and I really did not want to talk to anyone.*” [9]

“*One of my good friends I play volley ball with, comes over to the house a lot, and he’s a really good guy. One time I had to go somewhere and I couldn’t find a babysitter, and he was in the house and he goes, oh, I’ll watch her. And I said, no, and he goes, why not. And I said, I just don’t leave my daughter with a man. He thought it was a joke cause I said it in a joking way, but I told him I was serious. Even with my husband, until she was about nine months old I wouldn’t let her stay with him. I just wanted to protect her from anything. I guess part of it was thinking that he had never been around children before, and I was afraid that he’d do something on accident… I thought maybe he would abuse her. Finally I told him and I said, if you ever do anything to her I’ll kill you.*” [21]

**Creating an environment of trust** [7, 11] during the transition to parenthood and **building** **trusting relationships** **with care providers** [5, 32] were seen as important aspects of improving perinatal care, and relates to building relationships with providers.

“Creating an environment of trust means making it safe for the survivor through different mechanisms such as trusting the provider, trusting their bodies, being allowed time, being given options, choices, being asked being informed prior to, education and providers not making assumptions about the survivors. Establishing a relationship entails making eye contact, tone of voice, listening, hearing the survivor, appropriate (motherly) physical contact, respect, making a connection through conversation, individualizing care, and recognizing the survivor as a whole person. Part of this phenomenon involves creating an environment of trust.” [11]

“*I’ve been sexually abused, understand there are boundaries with a person like that ... besides for warning me, let me know, “Okay, I understand that you’ve been through this situation, I’m gonna do this, I’m not trying to make it uncomfortable for you.” Let the person like me, in my situation, know you’re not out to hurt me. That way I can gain that trust from that person and know that okay, it’s gonna be okay. And with each visit, not just sometimes, with each visit*.” [32]

“Each participant expressed that trust was an influential factor that affected their experience as they transitioned to parenthood. Although trust itself influenced the experience for each participant, it influenced each participant in a unique manner. Trust involved trusting themselves to not perpetuate the cycle, trusting that they had an adequate knowledge base from which to draw upon to be an effective parent, trusting their spouse to provide support and effectively parent, trusting their support systems, trusting that reaching out for support will result in a positive outcome, trusting that others will understand, and trusting that others can help. Trust can be placed on a continuum from being unable to trust, *‘I learned not to trust people so I didn’t really reach out when I really needed advice’*, to being able to trust, *‘eventually they had a worker come out that I felt like I could trust more, you know, and I think it is getting better’*. The ability to trust influenced the child maltreatment survivor’s capacity to connect with others and obtain support which ultimately influenced their ability.” [7]

**Regaining a sense of safety** [6, 22] was described as a positive aspect of pregnancy and birth by some parents.

“Some of the women in this study also felt that pregnancy was actually a way of keeping themselves safe. For example, Joleen commented, *‘no one hurts a pregnant woman’.* These thoughts were echoed by Cathy, who stated: *‘I felt like nobody would touch me because she is a pregnant woman—that kind of thing. Like nobody would attack you... you are treated with more kid gloves. I felt safe somehow and special’.*” [6]

“*I think I wanted to get pregnant because um… I wanted my abuser to leave me alone, so um and I wanted… I just wanted to feel safe. I thought I’m going to have to be pregnant all the time um to keep him away from me because that’s the only way.*” [22]

Parents also described a strong positive commitment to ensure **child safety and protection** [1, 2, 5, 7], which is related to **fear of repeating the past** (subtheme 1.3) and could lead to overprotection and challenges setting healthy boundaries.

“*There was really nowhere that I felt I was safe and where I was loved just for me. Now I have my own my family. This is my chance to make a safe space not just for my daughter, but for me and her father, and to create memories that are happy and healthy... not sort of tainted.*” [2]

“*[I want to learn] how to parent with healthy fear because I think that anyone who has had one of those traumatic events is going to have fear of it happening to their child and there has to be a line between protecting your child and overprotecting... learning how to let them live but still protect them and teach them*.” [5]

**Safety in perinatal care** [11, 23, 26] was identified as critical, and this is strongly related to analytic theme 5 of ‘empowerment, choice and control’ in care [11]. Parents also emphasised the importance of clinical **environments that foster safety** [5].

“An important aspect featured often was the need for safety, security and a sense of informality in contrast to traditional clinical sterility in order to promote wellness. When prompted to imagine the physical location of where desired services would be offered, women made comments such as *‘something homey’ and ‘not clinical’,* describing comfortable settings with sofas, cushions, and gardens; a safe place where they could bring their pets and children. *‘I think a place that’s nice and kind of cozy... that doesn’t necessarily feel like a doctor’s ofﬁce or a hospital so that you feel like you want to be there. I think that’s also important so that you might choose to go rather than sort of hide away... I think that’s important... a comfy atmosphere so that you really feel safe’*.” [5]

Richmond (2006) proposed mechanisms which demonstrated the link between showing regard for a person’s individuality and needs, and communicating respectfully and offering choice and control – which helped to foster trust and as sense of safety in care[11]. Richmond [11] also described how parents discerned and assessed safety, which had significant implications for disclosure:

“Discerning safety is a core category because participants spend much time assessing or discerning personal safety for the child victim, survivor and pregnant female. They are discerning for the whole personhood. Discerning is the process where survivors use the eye and intellect to determine safety during pregnancy and childbirth. Discerning reflects the pregnant survivors’ mental workings to process not only if the provider, but the circumstance is one in which they feel is safest for them. Discerning occurred without disclosure. …. Informing survivors of any forthcoming procedure and telling them exactly what is being done as it is being accomplished is regard for their personhood and a major safety concept. Experiencing no surprises to their person or in circumstances gives a foundation of trust, thus safety. Faith explains about her first pregnancy and how she felt safe during care even at a young age. *‘I appreciated that he always would tell me what he was going to do before he touched me or did anything and that helped a lot, even though it was very difficult’.* Another participant, Terri, speaks of feeling safe with a practitioner with breastfeeding. Terri states, *She’ll tell you, ‘Okay, well I am gonna touch you right here and this is what I’m going to do and this is what it’s gonna feel like. If you don’t feel this symptom, then we’ll try it’.*” [11]

Some parents chose isolation and care options like **home birth** (subtheme 5.1) to foster choice and control and a sense of safety.

“*My isolated situation in which I was living was protecting myself from the outside world because I knew that I could cope in a situation where I’m isolated because I’ve only got me to deal with then and I could sort of hide, I could go bush, I could do what I had to within my own isolation.*” [26]

**Protecting the child or ensuring safety** was also seen as an important enabler for healing [2, 7] and relates to **wanting to parent differently** (subtheme 1.3).

“During the transition to parenthood, child maltreatment survivors reported formulating goals. The goals and desires expressed by the child maltreatment survivors were significantly influenced by the child maltreatment survivor’s past experiences within their family of origin. Child maltreatment survivors who experienced a lack of protection from others formulated goals related to protecting their child, *‘I wanted to protect my kids even if it was from me’.* Each survivor’s goals were directly related to their past experiences of child maltreatment and had the same message, *‘I didn’t want to be like my parents*’. The goal of every participant was to become, or achieve an identity, that did not resemble the identity of the perpetrator parent(s).” [7]

This included developing strategies to protect their child from themselves, which relates to **fear of repeating the past** (subtheme 1.3):

*“*One participant would *‘keep it in my head like people are always watching me, I kind of keep in my head that maybe there is a nanny cam you know, in my mind I try to create this idea that I was being watched so that I wouldn’t hit him, and wouldn’t get frustrated with him’.* This participant found a creative way to protect her child.” [7]

### 6.2 The external world around them

Parents described many challenges in their **external environment** [8] that are likely to impact on their ongoing sense of **safety**, and highlight the need for provision of external support for this population group. Challenges include **housing instability** [4, 8, 14] and **financial** **challenges** [2, 8, 16], particularly for adolescent mothers.

“*When I find out that I was pregnant it came as a shock because I didn’t have anywhere to live, no place to stay. It was hard. No one wants a pregnant woman in their house so the time came when I had no choice and I went to the children protection services. I looked for my mum but didn’t find her. She had moved, that’s why I couldn’t find her. I went to the Children’s Protection Services and they sent me to the care institution.*” [14]

“Heidi had been living almost exclusively on the streets for the past 3 years, and she had run away several times in her early teens. With this pregnancy, she described making the rounds of the emergency night shelters for youth that are run by churches, interspersed with nights staying with friends or staying in the “squats,” abandoned houses taken over by homeless people at night. *‘It’s colder in the squats, and it smells. And in the shelters you have less chance of getting messed with from the cops, but in the squats you have more of a chance’.*” [8]

“Many of the participants had also made money as part of the informal or underground economy. Keshia had supported herself at 14 by dealing drugs. Brandy had let several people share her apartment, and she financed a portion of her drug use through hanging out with dealers and their girlfriends. Heidi described “*spanging*” or begging for spare change as a way to make money. She said many street kids spanged money to buy food, but being pregnant, she received twice as much change as most others did. She emphasized spanging was primarily for getting food when other sources of food were not available. There were a variety of sources she used, such as drop-in youth centers, free meal programs at various churches, and the shelters, but her choices were limited by lack of a place to store food. Both she and Brandy described “dumpster-diving,” that is, getting food that had been thrown away by restaurants at closing as a final desperate strategy when other resources were not available. Most of the participants said they had signed up for the food supplement program - Women, Infants and Children (WIC), but it was a challenge for those without refrigeration to store milk, eggs, and cheese. Heidi said that sometimes the WIC staff would give her several vouchers, one each for a small container of milk or package of cheese, so she could extend the food over the course of the month. Otherwise, she would get the food and share it among her street friends, and they in turn would share food with her when she needed it.[8]”

Perceptions of **stigma or judgement** [3, 4, 6-8, 18, 25], including **racism and discrimination** [8], were also described as an issue particularly affecting adolescent mothers. This impacted on challenges with **disclosure of abuse history** (subtheme 5.3) as well as choices and experiences of **becoming pregnant** (subtheme 2.1).

“Two of the women described experiencing a particularly acute sense of stigmatisation around the time that their memories surfaced: *‘I suddenly felt, ‘I can't go outside, everybody knows about me, they're all talking about me’. If I saw people grouped together they were talking about me. They knew it! But I didn't know what they knew, but I knew they knew it’. ‘I was very aware in the early stages that there was no way I would look at anybody. It was like... just couldn't handle that because I thought that they could see inside to what was going on in my mind’.*” [18]

**“**The judgemental attitude of some staff towards teenaged mothers appeared to have changed little in the intervening years: *Sister* C, *bless her, sitting at the desk... a* *big old dragon, didn't like unmarried mothers... didn't like young pregnant girls... you know, notes were slammed down on the desk, um, 'have you got your urine sample?'*” [18]

“Brandy struggled against both her mother’s perception of teen mothers as “*flaky*” and irresponsible, and her own observations of teen mothers who were drug-involved. She described her mother’s reaction when she first told her she was pregnant this way: *‘I could totally tell she was expecting me to be some like flaky, dumb, teenage mom and pawn the kid off on grandma, which you know, so many people do’*.” [8]

“*Ain’t nobody helping nobody black, you go to a white community, and they got stuff for kids, but you go to black, you go where I’m from, the kids just stuck with they doped-out momma, you know, ain’t no CPS came, but I’m living up here in Seattle and as soon as something go wrong with me, all these white people from CPS gonna come in and try to rush in and see what’s wrong with me and my baby*.” [8]

Parents described mixed experiences in relation to **education and employment**, with parents seeing this as a chance for a fresh start [2, 8], but also described challenges due to lack of education or employment [4, 8]. Again, this was particularly relevant for adolescent mothers who were at a critical education stage, and now having to juggle two important life transitions simultaneously.

“Nicole explained that after being put on bed rest due to her medical conditions (i.e., low placenta and symphysis pubis separation), she dropped out of school. Arianna and Jaylyn, too, dropped out of school during their pregnancy. Jaylyn tried to obtain employment, but was unsuccessful. Arianna, on the other hand, chose and was able to work during her pregnancy. Tiana was pregnant throughout her senior year in high school. She stayed in school, however, determined to *‘graduate on time like everyone else’*. She reported having taken off only one week following the delivery of her child, and was back to school the following week. Ciara, on the other hand, stopped working and enrolled in school during her pregnancy. She explained: *‘In the beginning I was working. And then I ended up getting laid, well not laid off, my hours got cut, so I just told them I was gonna quit and started looking into schools... I was working at the time, but I couldn’t work in the food industry because it made me nauseated so I looked into going to school… So I registered into school a few months later and that’s about it.*” [4]

Reflecting some of these challenges in the external environment, parents noted the importance of **practical support** [8] during the perinatal period. Some adolescent mothers described **foster care** [8, 14] as a place of safety and source of security, with access to regular meals, shelter, transport and bathing facilities.

“Fátima who already had been in the institution said that, *‘The shelter is good. You have a place where you can stay which is better than being on the road*.” [14]

## **Theme 7. *‘Reweaving’ a future*: Managing distress and healing while becoming a parent is a personal ongoing and complex process requiring strength, hope and support.**

Transition to parenthood marks a time in a survivor’s life when they themselves are increasingly vulnerable (distress in pregnancy, fear, disempowered in care) and new fears for the safety of their child are exposed. At the same time, pregnancy is often a catalyst for seeking help and an opportunity for healing, to moving forward and making sense, to recovery. This analytic theme incorporates four descriptive subthemes of: *Distress symptoms, including fear and lack of trust*; *coping strategies*; *factors that help recovery, growth and healing*; and *the healing journey though pregnancy, birth and parenting.*

### 7.1 Managing distress symptoms, including fear and lack of trust

While some parents described **symptom reduction** [3] during pregnancy, most parents described a wide range of **distress symptoms** in pregnancy, birth or early postpartum [1-4, 6, 8, 9, 17, 18, 25, 26, 29, 35]. Mothers who had experienced child sexual abuse described particularly distressing symptoms during birth, which midwives perceived were impacting on the process of birth [35].

“Mental health issues ranged from severe disturbances of mental health to no disturbances of mental health. Mental health issues influenced the child maltreatment survivor’s ability to process their experiences, bond with their children and others, reach out for and accept support and practical help, trust their parental skills, feel understood, feel adequate as a parent, and adequately respond to the challenges associated with becoming a parent.” [7]

“In common with other survivors of childhood sexual abuse, the women in this study used denial, repression, minimization, detachment, and dissociation, all of which are psychological defense mechanisms, to cope with sexual abuse at the time it occurred, and at other times throughout their lives when they were psychologically and physically vulnerable, such as during the childbearing phases. Once the baby was born and women entered the postpartum and mothering phases, these coping strategies often resulted in the child being viewed as “invisible” and, at times, subjected to maternal “emotional and physical absence.” For the majority of women in this study, this resulted in profound feelings of maternal guilt, which, in turn, fueled their need to self protect and withdraw. For some, this even resulted in situations of child apprehension. As the pregnancy progressed, the women in this study experienced greater feelings of vulnerability, which were generally heightened during labor and birth and continued through the postpartum and mothering phases.” [6]

Distressing symptoms were grounded in **fear during pregnancy** [2, 4, 6, 18], including **fear** **in care** [2, 6, 18, 25, 31], and reflected survival and self-protective responses utilised to deal with abuse as a vulnerable child. These fears were related to **fear of repeating the past (**subtheme 1.3)**, fear of not being believed**, and **fear of losing the child (**subtheme 5.3).

“*I was very frightened. I was very, very frightened and the thought of going through childbirth terrified me. The thought of having people examining me terrified me. Nobody asks you whether it's all right.*” [18]

“*I found out I was pregnant and I had this really sinking feeling, a really heavy fear.. .1 wasn’t happy at all. I was worrying that I couldn’t be a good mother and things were happening to me now that I had no control over. I didn’t want a girl for sure! I worried that I couldn’t protect her and I really didn’t want anything bad happening to her. It was like I was living in the past all of a sudden. I think I decided ahead of time that if I did have a girl, my dad would never see her. Into this pregnancy I also got really, really depressed*.” [6]

“*Cos all they do is come in, like mess, fiddle with you, do things to you and then they don’t really tell you what they’re doing…and then they disappear again and leave you and it’s almost like you’re sort of waiting for the door to open. You don’t know who is gonna come through, what’s gonna happen, ‘n it is very, very frightening*” [31]

Specific distress symptoms parents described included **shame or humiliation** **in care** [6, 18], **negative self-belief** [5, 6, 8, 9, 11, 18] and **guilt** [11, 23, 25], with some women apologising to their provider for their body and normal function during pregnancy and birth.

“Amanda's sense of humiliation arose out of the lack of respect for her privacy and dignity and the rough, uncaring attitude of the practitioners. The result, she said, was to objectify and dehumanise her, mirroring her experience of abuse: *‘I felt like a piece of meat and I felt just like I had when I was being abuse’*.” [18]

“*I didn’t want the doctor down there [perineum] looking at me... touching me... I felt so dirty just spread out like that for everyone to see... I didn’t want them to have anything to do with my body but they kept touching me and telling me to just breathe through it. I puked right then and there... just like I used to do when he was touching me. I was so ashamed. I tried to tell them but they just didn’t listen. I felt so vulnerable and I had no one to turn to*.” [6]

“*I feel like I'm… like I should be in trouble like I should be punished, that I am bad and it's very hard to sort of like defect from that feeling which is guilt.*” [25]

**Anxiety and depression** [2, 6, 8], including **postpartum depression** impacting on bonding and child development [6, 17, 21] were frequently reported by parents in this review.

Parents described **avoidance or denial** [6, 10, 11, 18, 29, 30], **avoidance of care** [6, 11], and **numbing** [11, 18] as challenges, but also used as a *coping strategy* (subtheme 7.2) and impacting on **access to care** (subtheme 4.2). Seng [30] described this as the first of three stages in the healing continuum:

Women who *‘were not ready to know’* described compartmentalizing the abuse away from their everyday thoughts and lives. They depicted their childhood sexual abuse and pregnancy as being *‘in two separate spheres…[having] ﬂeeting memories…working hard to keep them apart’*. Although the women did not fully acknowledge the abuse, it still affected their childbearing. One woman noted, *‘I knew early that I was not going to deliver vaginally. I knew in my head that I was not going there. So that piece I connected… I don’t know that I drew a real direct line because of how vulnerable I felt. I wasn’t probably ready to acknowledge that… So it was knowing and not knowing at the same time’*.” [30]

“Although signs and symptoms could have been discernible to a health care provider who *‘had it on their radar screen’*, the women had reasons for trying to maintain a fragile equilibrium by dissociating their awareness, maintaining a delicate balance of *‘knowing and not knowing at the same time’*. For example, these women felt there were losses associated with dealing with posttraumatic stress they did not want to incur: *‘I couldn’t really enjoy the pregnant princess scenario’,* and *‘…I was really looking forward to the cuddling time with the baby and breast feeding…I didn’t expect this whole other ugliness’,* and *‘I felt cheated’,* and *‘It just gets exhausting…these ﬂashbacks and stuff…and I just broke down crying…and I’m just sick of having to deal with it’*.” [30]

Women frequently reported **re-experiencing** **or triggers** **during care** (sub-theme 4.1) and **re-experiencing** [2, 3, 6, 9, 11, 21, 26, 29, 32, 35], **triggers** [6, 18, 26], **nightmares** [6, 11], **intrusive thoughts or nightmares** [2, 3, 18], and **delayed memory** [26, 31, 35] during the pregnancy, birth and breastfeeding. Women described these experiences occurring in a number of situations, not just during intimate procedures such as vaginal examinations. The ‘trigger’ was an individual, personal experience for each woman. These experiences were associated with feelings of rage and anger, and linked to not wanting to repeat the past, and moving towards forgiveness and acceptance, as reality is confronted.

“*I was scared. I could hear other women screaming, obviously they were screaming because they were labouring too. But I didn't scream, I just swallowed all the sobs and cries because that was the way ... I ... did, as a* *child, swallowed all the sobs, the cries, when I was being abused. I was afraid, I was in pain, um... I had* a *mask over my face and my husband kept trying to put it on to my face which was again, you know, hands over your mouth, when you were being abused as* a *child to stop you shouting for help. So the whole experience was like being thrust back as an adult but still feeling like that helpless child in the dark and being so afraid and alone*.” [18]

“...*but – it was horrible and I – how it makes you feel… is how it made you feel when you were a child. It really does – although you know it's different circumstances, you know you're gonna have a baby and you know, you tell yourself this all the time… deep down you just turn yourself back into that seven year old that, that was treated so horribly and had things forced upon them that you just… was so unpleasant and that's what, what it reminds you of, it really is (voice breaks).*” [25]

“Being confined by an epidural was one such trigger: *‘If I’m stuck on a bed, an’ I can’t get out, that is just like horrible it is, and then people coming in the room all the time and it, it triggers flashbacks’.* Even though epidurals had been requested by women, the unanticipated consequence transported women back to their abuse*.* Sam went on to say: *‘…and it makes me be the kid again (hesitatingly) an’ I’m like ‘oh no! It’s happening again!’*” [31]

“*I felt like I was stuck, like... they made me be there, and that’s what happened in the past, that person [the abuser] was on top of me making me be there. It wasn’t voluntary, at the hospital it felt like the same thing [the rape] was happening all over again*.” [32]

“It was often during this period of early motherhood that a number of the mothers began to have flashbacks of recollection of their past sexual abuse. Doreen’s statement about her flashbacks were typical to those of other mothers: *‘I had those flashbacks or memories all the time. It was like daily struggle to stay on top. There wasn’t five minutes a day when I didn’t think about it, and having my baby daughter was comforting. And now it’s less, but I still think about it’.*” [21]

“Emily experienced ﬂashbacks for the ﬁrst time during pregnancy. She described them as physical and affective memories that became particularly distressing during breastfeeding: *‘*…*every time [the infant] would latch on to nurse I would just sort of be hit with these uncomfortable… kind of nauseating… I call them ﬂashbacks, but it’s not like I’m reliving the incident, it’s just sort of the physical manifestation of the incident. It’s really hard to describe… it just feels awful. I feel like turning into a little black hole and disappearing*.” [29]

Parents described **dissociation** [3, 6, 18, 26, 27, 29, 30] and **passivity** [11] as a challenge. However, a number of parents also described also using dissociation as a ‘coping strategy’ during intimate perinatal care procedures or experiences.

“*I can do it at the switch of a*... *press of a button... I can go off and just not be aware at all*... *which is quite useful sometimes*... *but at the same time you don't hear what people are saying and you don't really take anything in because you're not really there.*” [18]

**“**Sharon also found that dissociation formed an effective coping strategy during her first birth experience (a home birth), which she described as *'positive'* and *'affirming'*: *‘... I think when I was in labour I just* ... *thought, 'It's not happening to* me', *and just completely switched off* ... *and just laboured really quickly’*.” [18]

“In contrast, Lisa described birth as especially challenging because, while dissociation was a tried and tested way for her to shut down painful moments in her life, and she experienced a disconnection from her body during her labor and birth, she also felt her heart was “in there.” Giving birth involved feeling deep emotion and love for her child, so dissociation was problematic: *From my thighs to my shoulders or below my shoulders is not mine, it never has been so go ahead and take it. I believe that’s what happened when I had a... something is sort of going on down there and everything wasn’t mine anymore... Too bad my heart was in there. It really sucks that my heart was in there with a... it was like I was fighting to have my own body back but just couldn’t. It seems like whatever I could do I have been ripped off, I mostly just shut down, I just do everything that I am supposed to do that came naturally but my heart was in that part of my body... in this frozen part of my body, in that part of the body that isn’t mine anymore... he was in there, and I couldn’t use it even though I knew I could deal with my heart, I couldn’t deal with the feelings... I was raped in a sense... not that I was physically raped, I was raped of the whole experience... 1 was ripped off... it was taken away from me because I brought up so much resistance to anything that happens to that part of my body that I couldn’t experience it because I didn’t know what was going on... I didn’t know the difference... I didn’t know that having this baby was any different than anything else going on with me from the waist down. Maybe if I knew and it was told to me continually through my pregnancy that this is because of your abuse, this is different because of it... then it’d be OK*.” [6]

Many parents described challenges with **substance use in pregnancy** [2, 8, 14, 29, 30, 32] as an **avoidant** **or numbing** strategy (discussed above) to try to help manage distressing symptoms. However, parents also described internal conflict and being worried about the impact on their baby, and also the impacts on pain management in labour for parents recovering from addiction.

“Some spoke of continued substance use during pregnancy to help them deal with their anxiety and flashbacks. A few stopped using illicit drugs and alcohol during pregnancy but turned to other avoidant coping strategies (e.g. food and tobacco) as a means of self-soothing” [32].

“Mary identified as an alcoholic, serious enough to get ‘*the shakes and stuff, and withdrawals*’ whenever she had tried to quit. She had continued to drink during her first pregnancy, but she stopped when she found out she was pregnant this time. *‘I kind of got tired of it. I was still drinking because it was just like something, you’re addicted, and even though I didn’t want to, it was like, I had to or something... I kinda switched addictions, though, I started smoking weed, and then, it was hard, it’s hard to stop smoking weed, but it’s, I quit drinking....But it’s not hard for me to not drink now, ‘cause I don’t even feel like drinking, and then I see people drinking and they look stupid to me’*.” [8]

“Claire said that her sobriety was important to plans for labour pain management because: *I am a recovering alcoholic, so I didn’t want to take any [narcotics or sedatives]… At that point I was ﬁve years sober, so I didn’t want to do any of that.*” [29]

### 7.2 Coping strategies

Parents described using a range of **coping strategies** [3, 6] for managing symptoms of distress and challenges during this transition. For many parents, pregnancy was seen as a time to begin healing which meant they were looking for coping strategies to support healing and change. This relates to subtheme 3.2, *relationships with self,* and developing strategies for managing their internal world.

“Pregnancy seemed to be an opportune time for the participants to begin the healing process since many of them were highly motivated to be healthy and to make a *‘fresh start’*. They wanted pregnancy to be ‘*pure’*, and this meant utilizing positive coping behaviors rather than self-abusive behaviors in order to deal with their emotional pain.” [6]

Coping strategies included **managing, containing or controlling trauma** [1] (which links to **protecting the child and ensuring safety**, and **being a good mother** (subthemes 6.1 and 1.2), as well as specific **coping strategies during** **vaginal examinations** [23] and **birth** [6, 11, 26], which included consciously using **dissociation** and having **control** (subtheme 7.1 and 5.1).

“Many women described an array of strategies they used to deliberately contain the trauma that continued to shape, and at times dominate, their lives. This process was evident in their voices as they shared their stories, and we were struck by the very deliberate, determined, and difficult work the process of containment entailed. The women exercised enormous creativity as they sought ways to live with their past, while carrying on with their lives in the present and imagined future. Strategies that reflected this process included blocking it out, deliberately blurring, reframing, and confronting the memories. As one participant who was a survivor of CSA said *‘You’re always thinking about it. ‘Oh, I have this trauma, blah, blah, blah’, so, you’re just stressing yourself, and not just you. There’s a little, like a little life, a little life inside you, so you don’t have to think about it. You have to block that feeling’*. Through the process of deliberately containing the trauma, the women felt able to protect themselves, their developing fetus, and others from whatever expression or outburst, fears, and anxieties that might emerge should they fail to contain them. Underlying this process were dominant discourses of what it meant to be a good (pregnant) woman, which made a display of anger or rage unacceptable. If they did not contain the trauma, they would be viewed as mad, bad, hysterical, and overly emotional.” [1]

“Despite her preparation and the strategies she had in place, she still struggled with required procedures when her ability to control the procedure was removed. *‘There was one bad experience during the IVF treatments when one of the nurses rushed me.* *Normally I keep control of the speed of vaginal examinations and tell them to stop. One of the nurses didn’t hear me, and I went into a trauma response. It’s pretty spooky because the ﬁrst thing that happens is, I lose eye contact, I can’t communicate verbally, I just completely shut down... the worst extent is total paralysis. I shake uncontrollably and then I am paralyzed, I don’t have any control over my whole body. My whole body is paralyzed; I can hear, but I can’t talk’.*” [23]

“Thus, Genieve worked all day, went home to take a bath, wash her long hair and got all of my things taken care of and came back and had the baby. Upon entering the labor unit she wanted to control her ability to walk through labor and not be in bed. *‘I really made sure, I was pretty adamant, but I was kind and I said, ‘You know, doctor said I could walk, so I have to walk.’ I just wanted to let everybody know I’ll do anything you say, but I have to walk. I just knew if I laid in bed, I wouldn’t be a very good patient, I knew that. I think now that I look back, it might have brought up memories’*.” [11]

Other coping strategies parents used included **establishing healthy boundaries or balance** [6], drawing on **spirituality or faith** [8] , and **self-help or care strategies** [3, 6, 8], including **reading** [2]. These strategies link to **learning about parenting** and **parenting ability** (subthemes 2.4 and 2.2).

“The major element that enabled these women to achieve a sense of balance was their coping strategies, which, critically, were stronger than the triggers associated with their abuse. *‘Physically having a child was a really profound experience for connecting with my inner child, it was profound. I thought I wasn’t prepared for that or I didn’t understand the magnitude until you actually give birth and you see this child and that sense of being overwhelmed. This it also helped me to honor that inner child and imagine what I was like as an infant type thing so it helped melt it together and you know again the innate wisdom that babies have like how smart they are about what they need and I was being able to live with a child who knows innately what they need and this has taught me so much, how they survive and how they thrive. I feel like I have finally made it. I feel safe. I feel balanced’*.” [6]

“*My spirituality is the center of my life, and everything that I do revolves around that and my own, my beliefs, and so my world view is very much, you know, goes hand in hand with my spiritual view... my spiritual beliefs are what have made it possible for me to make it as far as I have. You know, if I didn’t believe in something I probably would have killed myself years ago, because, this world’s a little bit too fucked up, life’s a little bit too crazy.*” [8]

“*Because it was just such a process of withdrawal from society, really, in a lot of ways, because I was only capable of very little, and large capacity to love and give, but with very little physical ability. And that's pretty scary. And so now, to be in this place is just unbelievable. I just started realizing. I'm teaching myself the guitar, teaching myself the recorder. I take care of my daughter 24 hours a day, I do all the cooking, and I cook whole foods, we don't eat anything really that's processed, other than ice cream and crackers. So I'm doing lots of things, and that's amazing. And like I can see now why other people make decisions. My perspective was so skewed back then. And not that I didn't try to see other people's perspectives, but it's different to be healthy and sort of look at adults and the way they live their lives and go, Okay, I can see why people do this and this. Just all kinds of things... It's just it's different. And it's different to feel healthy and feel like my body's normal: that I can produce a child, that she's o.k., and that I can have a normal life*.” [3]

“*Sometimes I just stay home and read literature... that kind of helps me heal*.” [2]

### 7.3 Factors that help recovery, growth and healing

In line with coping strategies, parents described many factors that they felt helped recovery and healing. **Making sense of trauma** [1, 3, 6, 7, 23] was identified by many parents as critical to recovery. This links to understanding and **learning about parenting**, and the transitional role of becoming a parent (theme 2). However, parents described internal conflict between being a ‘**survivor’ and ‘victim’** [11] and there was variability in parents **understandings of trauma** [30] that was related to different stages of healing.

“In order to move beyond being a survivor, women need to gain an increasing understanding of their own experience, make sense of the various ways in which they have been affected by the abuse, and recognise the various strategies they use/have used to cope. As the women in this study have shared, the process is difficult and each survivor faces serious struggles when trying to make sense of her experience in order to build on those “untouched” parts of herself and break free of the cycle of abuse. The women survivors in this study found and used what was helpful, and rejected what was not. Realizing that the abuse was not their fault was for many of the women a key to understanding their own responses to the abuse. They began to recognize that their behaviors, positive or otherwise, constituted strategies for coping with the abuse, and were not signs of fundamental flaws in their personalities.” [6]

“*Knowing myself and being able to connect what I went through as a kid, and process it, and feel it, and put it out there without judgment, has completely affected who I am as a parent*.” [7]

“At the time of pregnancy, some women had years of psychotherapy in which they became extremely knowledgeable and articulate about effects of abuse trauma. Others had only vague understanding of abuse effects (eg, ﬂeeting ﬂashbacks they were trying to ignore, memory of episodes without any affect) or of psychosocial problems (eg, being a “sex addict,” being suicidal starting at age 5). How much an individual woman knew about trauma generally and about the effects of her trauma history on her life inﬂuenced her ability to seek the health care she wanted. These 15 women varied in the extent to which they experienced abuse-related difﬁculties or posttraumatic stress symptoms during their pregnancies. However, what really seemed to matter was, how much they were aware that their trauma history might affect childbearing and whether they could bring this trauma knowledge to bear on what they were experiencing during pregnancy. For example, a woman who knew she dissociated when in stressful or painful situations seemed not to apply that self-knowledge when anticipating labor. The women’s knowledge of and understanding about the potential effects of their trauma histories on childbearing inﬂuenced many of their choices related to childbearing care, including the type of health care provider, setting for birth, and some lifestyle choices.” [30]

For many parents, becoming pregnant was a **catalyst to seek support** [3, 5] and many had sought **clinical therapy** [1, 5-7, 11, 32]. This links with theme 1, *new opportunities*, with some parents seeking to repair relationships and achieve a sense of acceptance of the past and forgiveness (discussed above).

*“*One woman told about her desire to see a counselor so she could do the work of healing before her baby was born: *I do want to see a counselor, because I want to make sure that I’m prepared for when the baby is born, and that I don’t have any hard feelings based on my past. I want to forget about it. Forgive and forget. You can’t move on. You don’t move on, you’re stuck and you’ll always be unhappy. And then you won’t be able to love yourself.*” [1]

However, some parents reported **ambivalence in seeking help** despite a desire for healing [5].

There were also mixed views about the value of **peer support** [5, 33], **parenting groups** [2, 5, 21, 34] and **trauma groups** [2, 6] for helping parents **making sense of trauma** and **developing relationships with others** (subtheme 3.6). Having a sense of ‘normality’ and an opportunity for **learning about parenting** with positive role models (which many parents described as lacking in their previous lives) were identified as important aspects of peer support and parenting groups. While having people who understood what parents had been through were an important aspect of trauma groups, there were also mixed feelings about the value of these for some parents.

“When asked which resources were most important to mention, several women highlighted peer support services. Peers were felt to be more accepting and empathic listeners than physicians, and interacting with a peer would not involve another power-discordant interaction such as counseling or psychiatric therapy.” [33]

“*One thing that helps me, at least for now, is that the mothers I met in the Lamaze group, we all keep in contact. We are always talking about, my son did this, or, did your son do that, and we talk about different issues of parenting. And I watch special TV programs. So, a combination of all these things are helping me to get a monitor as to what normalcy is. What I am constantly afraid of is not being able to tell normal development, even normal emotions.*” [21]

“*I don’t go to lecture or parents groups, and I wouldn’t. Should I spank my child or shouldn’t I. Well, it just has to be my decision, not a group or something. But if a teacher or somebody else thought there was something wrong with my daughter, I’d say, let’s discuss it and see what can be done about it. But I probably will feel, maybe I’ve done something wrong. I’d think, I spend all these years with her and I haven’t taught her something important.* Their reported sensitivity to criticism and their expressed fear that their inadequacy will be exposed, tended to isolate the mothers, thus inhibited the potential for social support and validation. Sometimes it worked to the detriment of the child.” [21]

“*I know it’s insane, it’s like how many groups do you have to talk to before you actually ‘qualify’, it’s what I call a victim Olympics.*” [6]

“Talking with other women or with sympathetic therapists provided welcome relief and was a source of strength, because in this context the women were believed and accepted, and received some practical advice. *‘Right from the beginning I attended mothers’ groups and parenting crisis groups and Project Parent… I really tried to find out what was normal, what wasn’t acceptable but you know it was really difficult because everybody projected a different story—there was no book’. ‘In the area that I live they have a program called “Healthy Babies, Healthy Children” and they have these home visitors and it is basically an experienced mom that comes around and just sits and talks with you for a while. She was very helpful just letting me know that it’s normal and just sitting and talking with me’*.” [6]

Parents also reported seeing value in emergent or **non-clinical therapies** [3, 5], including **art therapy** [32] and **bodywork** [3], which illustrates the challenges parents have feeling safe while being touched [6]**.**

“As they imagined healing from traumas that cause ongoing distress, 25% of the women identiﬁed activities that helped them feel better about their bodies, such as yoga, exercise, dancing, nutrition, salon services and, perhaps surprisingly, massage. Although at least one woman mentioned that they felt uncomfortable with physical touch, she also imagined that massage had the potential to be both healing and relaxing, if provided in a safe environment... ‘*Something that makes your body feel good and clean – just treating your body – the whole body’*.” [5]

“She stated that when she feels stressed or anxious she “*draws her feelings’*.” [32]

“Fay explained how bodywork was an invaluable part of her healing process. *‘But I did it because I needed to, and I was paying for acupuncture, and I was paying for therapy, and I took all of my money and funneled it back into me. I was making a lot of money but it was going all back into me, and I was getting massage...But it took me a long time to be able to let somebody touch me. The first time I had a massage I was really freaking out... But it was really helpful. And then, it just took me a while to get used to it. And acupuncture was even scarier in some ways, and some ways not. And everything was sort of a gradation-it was all part of the healing process and I couldn't really see it at the tim*e*’*.” [3]

Other factors that parents noted as important included **allowing time** [11], **acceptance or forgiveness** [3] (which links to relationships), and **helping others** [3, 8], which was a motivation in participating in the research for some, and links to **recovery** and **making sense of trauma** (discussed above).

“*I’ve tried reading on it, but when I read on it I get depressed. I’ve tried therapy, I couldn’t do it. So, I just try to do a little bit at a time and just not let it... not let it overwhelm me. Not let it dictate what I do or the decisions that I make so much*.” [11]

“*But I just made the decision that I was just going to accept it. It's a part of my life. I can't do anything about that, but I can deal with it and move on. And I basically just dealt with it in the fact that - Accepting that it has happened, accepting what it has done to me, how it's changed me.*” [3]

“Crystal and Jack wanted to become foster parents to provide respite foster care. In her third interview, Brandy talked about wanting to help out other young women like herself, once she got her own life together and stable. Keshia also wanted to take in foster children, but had an additional, grander plan; she wanted to create a camp for low-income youth.” [8]

### 7.4 The healing journey though pregnancy, birth and parenting

Many parents in this review described the experience of **healing through becoming a parent** [2, 3, 5, 6, 8-10, 22, 27] as a positive life changing journey. This links to the descriptive subtheme of *new experiences of love or joy* (subtheme 3.1), and while this brought up feelings of internal conflict and memories of their own childhood, these were largely overwhelmed by positive feelings of love for their child. This also links to theme 1, *new beginnings* and theme 2, *changing roles and identities*.

“*I wouldn’t be without any of them now and they’re an absolute god send to me they keep me alive.” “It is a life changing experience you know having a baby really changes your life luckily for me it has done for the positive um… it was a journey a good one I wouldn’t change it for the world*.” [22]

“*Now that I ’m a mother, I’m a lot stronger. I am able to see rational, normal boundaries for my daughter and because of that, i t’s a lot easier to identify rational, sane, healthy boundaries for me. I am her mother, her protector. For a long time I thought that I was intrinsically flawed, that I came into this world somehow broken or damaged or something, and that was what drew the abuse to me. I have been sexually abused by a few people in my lifetime and irrespective of my outward appearances, of what I said or how hard I worked, there was something about me dirty and broken that I needed to hide. But when I had Emma, I realized that we all come into this world perfect, and that the flaw wasn’t in me, it is in my abusers. It has been really difficult giving up this long-held belief that there was something wrong with me. I still... I mean there’s still pieces of it that I haven’t gotten rid of... but having Emma has allowed me to see myself differently.*” [2]

“*For me, I know that things are getting bad when all of a sudden this little voice in the back of my brain says 'Well, if it gets too bad you can always kill yourself." I have never tried it. I don't consider myself to be suicidal. But it's like for me that's a barometer. It comes from nowhere. It's like that's okay because you can always kill yourself. Would I ever do it? No. Would I ever hurt my husband like that? No. Absolutely not. But it's the barometer. And now I think that voice is absolutely dead as far as a barometer. Because as much as I would never do it to my husband, still there was always like "If you really had to, though kind of feeling. With a daughter, I would never in a million years do that to her. That voice is gone. So whether ifs truly gone, or whether it will pop up as my barometer still, I don't know*.” [3]

“*All of my efforts to heal myself have made this a really important thing for me, you know, all of the energy that I’ve spent to become who I am, and do what I need to do, have made this be the inevitable conclusion*.” [8]

Parents described a healing process of **recovery** [9, 32] and **post-traumatic growth** [9, 30]. Again, these link strongly to themes 1 and 2, with a sense of ‘being resilient and strong’.

“*It took some years to get over and get some help and talking to support... now I can say I feel like I really am [over it] because I can freely speak about it, and I am not very emotional about it or depressed. I’m able to speak out and not hold or withdraw anything from anybody about it... Just ‘cause this happened in my childhood don’t mean I gotta carry it over to adulthood motherhood.*” [32]

“Despite reporting negative consequences and effects, all ten participants recounted a personal process by which they were able to move forward from their sexual trauma experiences. Based on their reports of the positive effects that the sexual trauma had, four sub-themes emerged: gaining a new perspective on life, ﬁnding an inner strength, using a support system, and letting it out.”[9]

“*…it does have a positive impact, because I had to stop and look at myself and be like, that’s not what I want to have to deal with every day. So, it made me stop and think about what I was doing with my life, and it made me change it*.” [9]

“All ten participants shared that because of their sexual trauma, they began to see and experience themselves as stronger beings. One participant shared: *‘I think it made me stronger… ’cause if I can get through that, I can get through anything, honestly’*. Another woman recounted how her newfound strength brought positive meaning to her previous rape experience: *‘It’s made me stronger as a person, so I think that was the reason (it happened). Just to prove to myself that I’m a strong person. If I can make it through that, I can make it through anything’.* Although participants reported that the past sexual trauma experience will always be a negative element or part of their life, they all described striving not to let it overtake them.” [9]

“*I went to college and I had a very promiscuous life… I just was not good to myself… so [pregnancy] comes along, and I quit all the things I was doing… I quit drinking beer, I quit smoking cigarettes, I quit smoking pot, I just…healed myself*.” [30]

Seng [30] described the healing journey as a continuum across three stages: 1) women far along in recovery, 2) women who were not safe, and 3) women who were not ready to “know”:

“The role of health care providers depends on where on the continuum the woman falls in each of these three assessment areas. It is not so much “who” the woman is that should drive the provider’s response, but “where” she is in the life span process of recovering from the negative impact of past or current abuse trauma. Where the woman was in her recovery from trauma then signiﬁcantly affected both what her goals were and how she negotiated the many changes and challenges of the childbearing year.” One woman talked about having to change to a midwife after 6 months because “*the doctor was kind of cold, not personable at all, and those feelings [emotional memory of being abused, shame, vulnerability, nakedness] would come back to me in his ofﬁce, and I found myself crying at every visit*….” She moved from not really understanding the impact of the childhood sexual abuse on her (starting off like the women in this *“knowing and not knowin*g” state) to confronting what was happening and arranging the care she needed with a midwife and a therapist. She described this begrudging but accomplished shift, “…*I kind of knew in some way it was affecting me, but I just couldn’t connect the dots ever…but when I got pregnant it all just came out, came clear, and it was hard, and I’m grateful…and I think it’s going to help me grow past it and deal with it…but pregnancy is enough to deal with.”* [30]

Several parents in this review described the **healing process of birth** (subtheme 4.3), however many other women reported challenges with triggers and other distressing aspects of the birth experience (subtheme 7.1).

“While Fay anticipated difficulty and complication as she had seen in so many areas of her life, she felt transformed by this birth, as though it had made her complete*. I remember laying there right before I started laboring, like really laboring, and I was such a different person. I was still like half a person. When I look back I see half a person, just not there. I was sick last week-not last week-the 'flu everyone had, really sick, and my stomach was hurting, and all I could think of was Please God, don't send me back there, please don't make me that person I was. I can't do it. You can't like turn the light on completely and let me have life, then take it away. Not again.*” [3]

“*It was just the most wonderful, magical experience of my whole life. I feel like things changed for me right there.*” [6]

Many parents emphasised the importance of **resilience or being strong** [4, 6, 32], and reporting a sense of **survival fostering resilience and agency** [2, 21]. This may relate to the need to draw on internal resources, as external support may be perceived as insufficient or create other challenges, due to issues with **relationships** (theme 3) [6].

“*It [childhood sexual abuse and its effects] is never-ending, it lowers your self esteem, your sense of identity, your playfulness, and your whole childhood is taken away from you... it’s like this constant reminder. Recovery is not possible, but I can learn from this and heal from this.*” [6]

“*Not everybody that gets abused ends up on skid row. We can make choices. We can... I am a very single minded woman and I think that part of that comes from the abuse. I did really well in school and did really well in anything that I have endeavored to do because I concentrate. Getting over sexual abuse is a life long process. There isn’t sort of an end point... the road to healing is life long. I think that maybe you were a victim when it happened but you know, you’re not a victim now. And even survivor... I have trouble with being called a survivor because that sounds like you are just treading water. I’d like to be called ‘the Victor’*.” [6]

“*I had to realize my own strength... and that’s a big part of the journey... One of the best learning experiences I have had is becoming a mother. I have learned what I can handle, what I can deal with, how to better relate with other people through that whole state of exhaustion and you can still handle life. It’s also been one of the greatest challenges that I have ever had to face which at first, yeah it’s like a panic that you don’t ever get away from but then as you realize what you can do, and have to do, then you become a stronger person for it as well. It was when I started having a real sense of confidence that my history started, that I started remembering things. So whether I became stronger as a person myself to feel like I could handle memories of that kind or that sort of thing.*” [6]

“*We raised ourselves. The fact that I had children to raise... I mean I think that’s the best thing that ever happened to me. Not only did I have to do it all myself but I had other people depending on me... That was amazing because when you have people depending on you there’s nothing you can’t do. You have to do it and that’s what gave me the gumption. I mean I look at my other siblings who didn’t have to raise other children, they did not... they weren’t able to... they didn’t succeed. They didn’t have to keep a piece of themselves out of the drama and trauma to go forward, to create a life for others. So they were one hundred percent buried in it. I see that now with other survivors. Those who had to look after others... there is a part of their self that just didn’t have time to get sick... I had to perform. I had to be able to make a lot of money to bring home food to put my brothers through school... to get the winter clothing for my little sister... to get her teeth fixed... Yeah. I was a great compartmentalizer because I had to be.*” [2]

“*I do know that certain people, certain relationships, certain situations - both good and bad - have made an impact on my life. My past is what helps me get through today. Everything that I have experienced - everything that I have been, uhm, subjected to - has helped me in one way or another. I got through it and I learned from it. So I try to look at, certain situations and things that happened and ask myself what did I learn from it? How did it change me? How did I get through that?... I have had a lot of obstacles and challenges in my life. I have been to hell and back... and have lived through things that maybe some people could not. I was lucky enough to get through those difficulties with the help of my family, my friends, and my own personal strength... Nobody else can do it for you*.” [2]

## **Summary of reflections on provider views in light of parents’ views**

Two studies also reported providers’ views. Saewyc [8] reported views of providers for children ‘ageing out’ of the out of home care system. There were no additional relevant insights for this review, however the views of providers did appear a little more pessimistic, than parents, about the future for pregnant adolescents ageing out of care.

Garratt [18] reported views of midwives who were also child sexual abuse survivors. These midwives highlight **barriers to being present in a professional role** and challenges when attending services for birth in **being seen as a worker and not a parent.**

“*It's just the level of the work, you know, you get thrown at you when you're down there [labour ward] so you end up looking after 2 or 3 women... a woman comes in perhaps quite frightened and... needing some support and I felt able to give it for a certain amount of time and then I've been called away because something else is happening somewhere else and by the time I've gone back, they've just lost it really and I don't feel I can get them back on... and you know, even if I did, it probably wouldn't last that long before I had to go again...*”

Midwife survivors had a unique insight into the perinatal care system, and were acutely aware of how perinatal **care can be reminiscent of abuse** and that **conventional practice is not trauma-informed**.

“*I find it almost impossible to work on labour ward. I work on a bank contract so I can work where I want to, and I avoid delivery suite like the plague really, because I just don't want to be involved in that... ritualised abuse really. You know, when I think how birth can be and when I think how birth is for the majority of women now, I just don't want to be involved in that at all.*”

“Sometimes, however, the pressure on them to conform proved overwhelming and gave rise to situations in which the midwives felt that they had acted abusively: *‘... in the early days... I constantly felt I was doing things I shouldn't have been doing. I can remember doing an episiotomy when I didn't want to but I'd got a member of staff behind me handing me the lignocaine and then the scissors’.*”

“Several of the interviewees did not find it easy to confront authority. Consequently, they superficially surrendered to the system whilst quietly working to support women behind closed labour ward doors. Sharon, who had qualified relatively recently, explained that she used her junior status and inexperience in order to avoid compliance with hospital policies when she felt they contradicted the women's wishes. Jo, a student at the time of her interview, would encourage the woman to follow her body's cues only when her mentor was out of the room. She was reluctant to take a confrontational stance because of her inability to challenge authority; consequently, she found a non­confrontational strategy to achieve her ends. Clearly, Jo felt it was not possible to represent the woman's interests at the same time as meeting the demands of the system and was therefore obliged to practise 'undercover' midwifery.”

“*I do get criticism sometimes and whatever, for not doing vaginal examinations as standard. You know when... you work in this big teaching hospital and you admit someone and you sort of come out the room and everyone looks at you because they want to know for the staffing and the workload and the blah, blah, blah, whether the woman's in labour or not and I say, 'Well, I don't know!’*”

“*I battled on, and fought for these women, yet working in this environment was destroying me.*”

Midwife survivors used their experiences as a positive force in guiding their approach to practice. This included giving women **choice and control**, **good communication,** **treating women as individuals** and **acting as advocates on women's behalf**, reinforcing findings from parents in this review.

“*I think that's the biggest thing that's come out of it [being a survivor of CSA] really... that I want them to have some power and I want them to feel good about themselves and their body and their experiences.*”

“Several of the midwives also described their willingness to be flexible in their approach to how and where to perform vaginal examinations. Some expressed the opinion that lying flat on a bed might be distressing for survivors of CSA and would therefore suggest alternatives that might be more acceptable. *‘... and I will also talk through it as I'm doing it - 'Is this comfortable? Is there a particular place where it would be better, for you to have this done?' So that if it's lying· flat on their back, which is the position that most people would have been abused in, then I will offer - you know - I will offer for them to tell me where's best for them to have it done. Standing, maybe sitting even because you can do a VE efficiently, maybe not as well as, but effectively to get a reasonable amount of information, which um... which is important. And if, if a woman has a technique of taking her through that, I will ask her about it - 'If you've had to have this done before, is there any way that you have found better in coping to have this done - i.e. holding my hand so that I'm only using one hand, or do you like me to look at you while talking?' So I take a lot of time about talking how to do this procedure before we even start.*”

“The trappings of medicalised birth which have become largely invisible to practitioners, may represent a powerful threat to women who have suffered sexual abuse: *‘A woman seeing a tube of KY gel might just freak her out. Especially if you were a child being abused and the abuser couldn't penetrate. Seeing a tube of like... or Vaseline, is a complete no-no.*”

**“**There was much discussion on explaining procedures and talking women through vaginal examinations. However, Kerry's was the lone voice that sounded a note of caution with the indiscriminate use of this approach: *Then, whilst I'm actually doing it I will ask her if she wants me to talk to her while I'm doing it to tell her what I can feel or 'would you prefer me not to?' because some abusers talk through what they're doing. And that might be distressing. My father used to do that to me*.”

“When asked how care for women might be improved, one suggested *'not to treat everyone*... *as*... *a protocol.*”

“Vickie explained that she always gave a little 'spiel' to women on admission: *There are lots of things in a hospital that we do as* a *matter of routine, but you don't have to have any of it and if you say to me that don't want it then you don't have to have it. It's as simple as that.***”**

**References**

1. Berman, H., et al., *Laboring to mother in the context of past trauma: the transition to motherhood.* Qual Health Res, 2014. **24**(9): p. 1253-64.

2. Lasiuk, G.C., *The lived experience of pregnancy and birthing of women with histories of childhood sexual abuse*. 2007, University of Alberta (Canada). p. 247 p-247 p.

3. Lee, S.R.C., *Survivors of childhood sexual abuse and the childbearing year.* Dissertation Abstracts International. Section B: The Sciences and Engineering, 2001. **62**(4-B): p. 2064.

4. McCoy, J.J., *Maternal perceptions and pregnancy experiences of former foster youth with histories of sexual abuse.* Dissertation Abstracts International. Section B: The Sciences and Engineering, 2015. **76**(4-B(E)).

5. Muzik, M., et al., *Perspectives on trauma-informed care from mothers with a history of childhood maltreatment: A qualitative study.* Child Abuse Negl, 2013. **37**(12): p. 1215-24.

6. Palmer, B.C., *The childbearing experience of women who are childhood sexual abuse survivors*. 2005, University of British Columbia: Canada. p. 361 p-361 p.

7. Roberts, R.E., *The survivors of child maltreatment description of the process of becoming a parent: A grounded theory study.* Dissertation Abstracts International. Section B: The Sciences and Engineering, 2011. **72**(6-B): p. 3765.

8. Saewyc, E.M., *Meanings of pregnancy and motherhood among out-of-home pregnant adolescents.* Dissertation Abstracts International. Section B: The Sciences and Engineering, 2000. **60**(11-B): p. 5437.

9. Schwerdtfeger, K.L. and K.S. Wampler, *Sexual trauma and pregnancy: A qualitative exploration of women's dual life experience.* Contemp Fam Ther, 2009. **31**(2): p. 100-122.

10. Williams, C. and S.W. Vines, *Broken past, fragile future: personal stories of high-risk adolescent mothers.* Journal of the Society of Pediatric Nurses, 1999. **4**(1): p. 15-23.

11. Richmond, K.K., *Being whole: Aligning personhoods to achieve successful childbirth with a history of childhood sexual abuse during perinatal services*. 2006, University of San Diego: United States of America. p. 110 p-110 p.

12. Kennedy, A.C., et al., *Risk chains over the life course among homeless urban adolescent mothers: altering their trajectories through formal support.* Child Youth Serv Rev, 2010. **32**(12): p. 1740-1749.

13. Coles, J., *Qualitative study of breastfeeding after childhood sexual assault.* J Hum Lact, 2009. **25**(3): p. 317-24.

14. Miura, P.O., L. Tardivo, and D.M.S. Barrientos, *Helplessness experienced by adolescent mothers and pregnant adolescents sheltered in institutions.* Cien Saude Colet, 2018. **23**(5): p. 1601-1610.

15. Swartz, N.E., D.J. Mercier, and M.A. Curran, *Influences of childhood abuse on parenting perspectives of pregnant cohabitors.* Journal of Family Violence, 2012. **27**(6): p. 597-606.

16. Swartz, N.E., D.J. Mercier, and M.A. Curran, *Influences of childhood abuse on parenting perspectives of pregnant cohabitors.* J Fam Violence, 2012. **27**(6): p. 597-606.

17. O'Brien, D.W., *A qualitative study of parenting by incest survivors.* Dissertation Abstracts International Section A: Humanities and Social Sciences, 1999. **59**(7-A): p. 2721.

18. Garratt, E.F., *The childbearing experiences of survivors of childhood sexual abuse.* Dissertation Abstracts International. Section C: Worldwide, 2018. **75**(4-C).

19. Montgomery, E., C. Pope, and J. Rogers, *The re-enactment of childhood sexual abuse in maternity care: A qualitative study.* BMC Pregnancy Childbirth, 2015. **15**: p. 194.

20. O'Brien, D.W., *A qualitative study of parenting by incest survivors.* Dissertation Abstracts International. Section A: Humanities and Social Sciences, 1999. **59**(7-A): p. 2721.

21. Cohen, T., *Experiences of motherhood among women who were victims of childhood incest.* Dissertation Abstracts International. Section B: Sciences and Engineering, 1987. **48**(4-B): p. 1148.

22. Byrne, J., C. Smart, and G. Watson, *"I felt like i was being abused all over again": How survivors of child sexual abuse make sense of the perinatal period through their narratives.* J Child Sex Abus, 2017. **26**(4): p. 465-486.

23. Coles, J. and K. Jones, *"Universal Precautions": Perinatal touch and examination after childhood sexual abuse.* Birth, 2009. **36**(3): p. 230-6.

24. Coles, J., *Qualitative study of breastfeeding after childhood sexual assault.* Journal of Human Lactation, 2009. **25**(3): p. 317-24.

25. Montgomery, E., C. Pope, and J. Rogers, *A feminist narrative study of the maternity care experiences of women who were sexually abused in childhood.* Midwifery, 2015. **31**(1): p. 54-60.

26. Parratt, J., *The experience of childbirth for survivors of incest.* Midwifery, 1994. **10**(1): p. 26-39.

27. Wood, K. and P. Van Esterik, *Infant feeding experiences of women who were sexually abused in childhood.* Can Fam Physician, 2010. **56**(4): p. e136-41.

28. Williams, C. and S.W. Vines, *Broken past, fragile future: Personal stories of high-risk adolescent mothers.* J Soc Pediatr Nurs, 1999. **4**(1): p. 15-23.

29. Seng, J.S., et al., *Abuse-related post-traumatic stress during the childbearing year.* J Adv Nurs, 2004. **46**(6): p. 604-13.

30. Seng, J.S., et al., *Abuse-related posttraumatic stress and desired maternity care practices: Women's perspectives.* J Midwifery Womens Health, 2002. **47**(5): p. 360-70.

31. Montgomery, E., C. Pope, and J. Rogers, *The re-enactment of childhood sexual abuse in maternity care: a qualitative study.* BMC Pregnancy & Childbirth, 2015. **15**: p. 194.

32. Roller, C.G., *Moving beyond the pain: Women's responses to the perinatal period after childhood sexual abuse.* J Midwifery Womens Health, 2011. **56**(5): p. 488-93.

33. White, A., M. Danis, and J. Gillece, *Abuse survivor perspectives on trauma inquiry in obstetrical practice.* Arch Womens Ment Health, 2016. **19**(2): p. 423-7.

34. Datta, J., et al., *Challenges faced by young mothers with a care history and views of stakeholders about the potential for Group Family Nurse Partnership to support their needs.* Child Soc, 2017. **31**(463-474).

35. Rhodes, N. and S. Hutchinson, *Labor experiences of childhood sexual abuse survivors.* Birth, 1994. **21**(4): p. 213-20.
